# Diverse LXG toxin and antitoxin systems specifically mediate intraspecies competition in *Bacillus subtilis* biofilms

**DOI:** 10.1371/journal.pgen.1009682

**Published:** 2021-07-19

**Authors:** Kazuo Kobayashi

**Affiliations:** Division of Biological Science, Department of Science and Technology, Nara Institute of Science & Technology, Ikoma, Nara, Japan; Danmarks Tekniske Universitet, DENMARK

## Abstract

Biofilms are multispecies communities, in which bacteria constantly compete with one another for resources and niches. Bacteria produce many antibiotics and toxins for competition. However, since biofilm cells exhibit increased tolerance to antimicrobials, their roles in biofilms remain controversial. Here, we showed that *Bacillus subtilis* produces multiple diverse polymorphic toxins, called LXG toxins, that contain N-terminal LXG delivery domains and diverse C-terminal toxin domains. Each *B*. *subtilis* strain possesses a distinct set of LXG toxin–antitoxin genes, the number and variation of which is sufficient to distinguish each strain. The *B*. *subtilis* strain NCIB3610 possesses six LXG toxin–antitoxin operons on its chromosome, and five of the toxins functioned as DNase. In competition assays, deletion mutants of any of the six LXG toxin–antitoxin operons were outcompeted by the wild-type strain. This phenotype was suppressed when the antitoxins were ectopically expressed in the deletion mutants. The fitness defect of the mutants was only observed in solid media that supported biofilm formation. Biofilm matrix polymers, exopolysaccharides and TasA protein polymers were required for LXG toxin function. These results indicate that LXG toxin-antitoxin systems specifically mediate intercellular competition between *B*. *subtilis* strains in biofilms. Mutual antagonism between some LXG toxin producers drove the spatial segregation of two strains in a biofilm, indicating that LXG toxins not only mediate competition in biofilms, but may also help to avoid warfare between strains in biofilms. LXG toxins from strain NCIB3610 were effective against some natural isolates, and thus LXG toxin–antitoxin systems have ecological impact. *B*. *subtilis* possesses another polymorphic toxin, WapA. WapA had toxic effects under planktonic growth conditions but not under biofilm conditions because exopolysaccharides and TasA protein polymers inhibited WapA function. These results indicate that *B*. *subtilis* uses two types of polymorphic toxins for competition, depending on the growth mode.

## Author summary

Biofilms are surface-associated multispecies communities, in which bacteria are protected by self-produced extracellular polymeric substances. In biofilms, bacteria constantly engage in intra- and interspecies competition. To minimize exploitation by competitors, bacteria produce a variety of antibiotics and toxins for competition. However, since biofilm cells exhibit increased tolerance to antimicrobials, the function of antibiotics and toxins in biofilms remains controversial. Therefore, the mechanisms underlying bacterial competition in biofilms remain to be investigated. We found that the soil bacterium *B*. *subtilis* produces polymorphic toxins, termed LXG toxins. LXG toxins are highly diversified among *B*. *subtilis* strains, and each *B*. *subtilis* strain possesses three to nine different LXG toxins. LXG toxins specifically mediate intraspecies competition in biofilms. Competition between some LXG toxin producers resulted in the spatial segregation of strains in biofilms, indicating that LXG toxins not only mediate competition, but also help to minimize warfare in biofilms. LXG toxins were effective against natural isolates of *B*. *subtilis*, suggesting that LXG toxin–antitoxin systems have ecological impact. Our results provide new insights into how bacteria survive competition in biofilms.

## Introduction

Like many life-forms, bacteria are social organisms that must constantly compete or cooperate with each other. These interactions play critical roles in surface-associated bacterial communities, termed biofilms. In biofilms, millions or billions of cells grow while adhering to neighboring cells or a surface, and these biofilm cells are encased in a matrix of extracellular polymeric substances [1], which protects biofilm cells from antibiotics, toxins, and the host immune system [2,3]. Environmental biofilms are usually composed of multiple bacterial species, which can allow for better nutrient utilization, degradation of toxic compounds, or resilience in the face of harsh environments [4]. Multispecies biofilms are believed to develop through a series of intra- and interspecies interactions. In these interactions, bacteria distance from or outcompete unfavorable competitors and promote beneficial neighbors to increase their fitness. Unfavorable competitors are often the same or closely related species because they share genetic traits and preferred niches [1,5]. Another kind of unfavorable competitors are “freeloading cheaters”, which consume extracellular products such as polymeric substances, enzymes, metabolites, and signaling molecules without contributing themselves [5]. One mechanism to exclude related competitors is kin discrimination, in which bacteria discriminate kin from nonkin and preferentially associate with close kin to form a cooperative group [5]. The exclusion of related competitors is also a preventive measure against potential cheaters, since related strains are better at exploiting extracellular products due to their genetic relatedness. However, competition and kin discrimination mechanisms in biofilms remain to be elucidated.

Bacteria have evolved diverse antibiotics and toxins to antagonize competitors and discriminate kin [6–11]. Among these, polymorphic toxins are widespread among bacteria and serve as weapons, especially for interbacterial competition between related strains [9–13]. Polymorphic toxins are multidomain proteins, which have common domain architecture, including N-terminal trafficking domains, various central regions, and C-terminal polymorphic toxin domains [12–14]. The N-terminal trafficking domains interact with chaperones or components of specialized secretion systems and govern the transport of toxins or toxin domains into recipient cells [12–14]. The C-terminal toxin domains have markedly been diversified and are indeed classified into over 150 distinct domains with diverse activities [12,13]. These toxins are also called contact-dependent inhibition (CDI) toxins because these toxins exert their effects when toxin producers physically make contact with other cells. Upon cell–cell contact, these toxins are delivered from toxin producers to adjacent cells across multiple cell envelopes by specialized secretion systems [14]. For example, type VI secretion systems, which are conserved in many Gram-negative bacteria, act as molecular syringes that directly inject toxins into adjacent cells using the energy of ATP hydrolysis [15,16]. The injected toxins suppress or kill recipient cells that do not express cognate antitoxins. Polymorphic toxin genes usually form operons with downstream antitoxin genes, which protect toxin producers from their own toxins. Antitoxins are also as diverse as toxin domains, and each antitoxin specifically neutralizes its cognate toxin by binding to the toxin domain [17]. The extensive diversification of toxin–antitoxin pairs allows each strain to have a unique set of toxin–antitoxin operons and thereby to discriminate between self and nonself or between kin and nonkin. Thus, bacterial strains, even though they are from the same species, exhibit incompatibility with each other if they have different polymorphic toxin–antitoxin operons. These polymorphic toxins may function effectively in biofilms, in which cells grow in direct contact with one another. However, although polymorphic toxin systems have been well-studied in many bacteria, especially in members of Proteobacteria, most studies have been conducted under planktonic growth conditions, not under biofilm conditions. To our knowledge, only a few polymorphic toxins were shown to influence biofilm communities through competition [11,18,19].

The Gram-positive soil bacterium *B*. *subtilis* forms robust biofilms, such as complexly structured colonies on solid media that support biofilm formation [20]. *B*. *subtilis* strains secrete various types of antibiotics and toxins, some of which were detected in biofilms [7,21,22]. These antimicrobials include nonribosomal peptide and polyketide antibiotics, protein-derived peptide toxins, CDI toxins, and lantibiotics [7,23–26]. The composition of antibiotic and toxin synthesis genes in *B*. *subtilis* genomes varies from strain to strain, and each *B*. *subtilis* strain often produces a distinct set of antimicrobials [7]. Previous studies under non-biofilm conditions indicate that *B*. *subtilis* uses a combination of multiple antimicrobials, rather than cell-surface receptors, to discriminate kin from nonkin strains [8,27,28]. Thus, *B*. *subtilis* employs an exclusive kin-discrimination system, and *B*. *subtilis* strains that produce different sets of antimicrobials exclude each other. Although this mechanism may operate in biofilms, cells in biofilms are protected by a biofilm matrix and therefore exhibit increased tolerance to antimicrobials [2,3]. In biofilms, the diffusion of antimicrobials is slower and limited by high cell density and biofilm matrix polymers, and charged antimicrobials are absorbed by biofilm matrix polymers [29–34]. Some antimicrobials cannot access their receptors on the cell surface in biofilms [35,36]. The delivery of some CDI toxins is physically inhibited by biofilm matrix polymers [36–38]. Thus, not all antibiotics and toxins produced by *B*. *subtilis* can work in biofilms. In fact, we previously showed that YIT and SDP peptide toxins of *B*. *subtilis* exhibited different properties in biofilms, although these toxins are synthesized by homologous operons, *yitPOM* and *sdpABC*, respectively [26]. Specifically, the YIT toxin exerted its toxicity in a biofilm-specific manner, whereas SDP toxin activity was inhibited in biofilms. Based on these observations, we predict that *B*. *subtilis* may produce special antibiotics and toxins that can function as biofilm-specific competition mechanisms. However, no such antibiotics and toxins have been reported.

**Fig 1:**
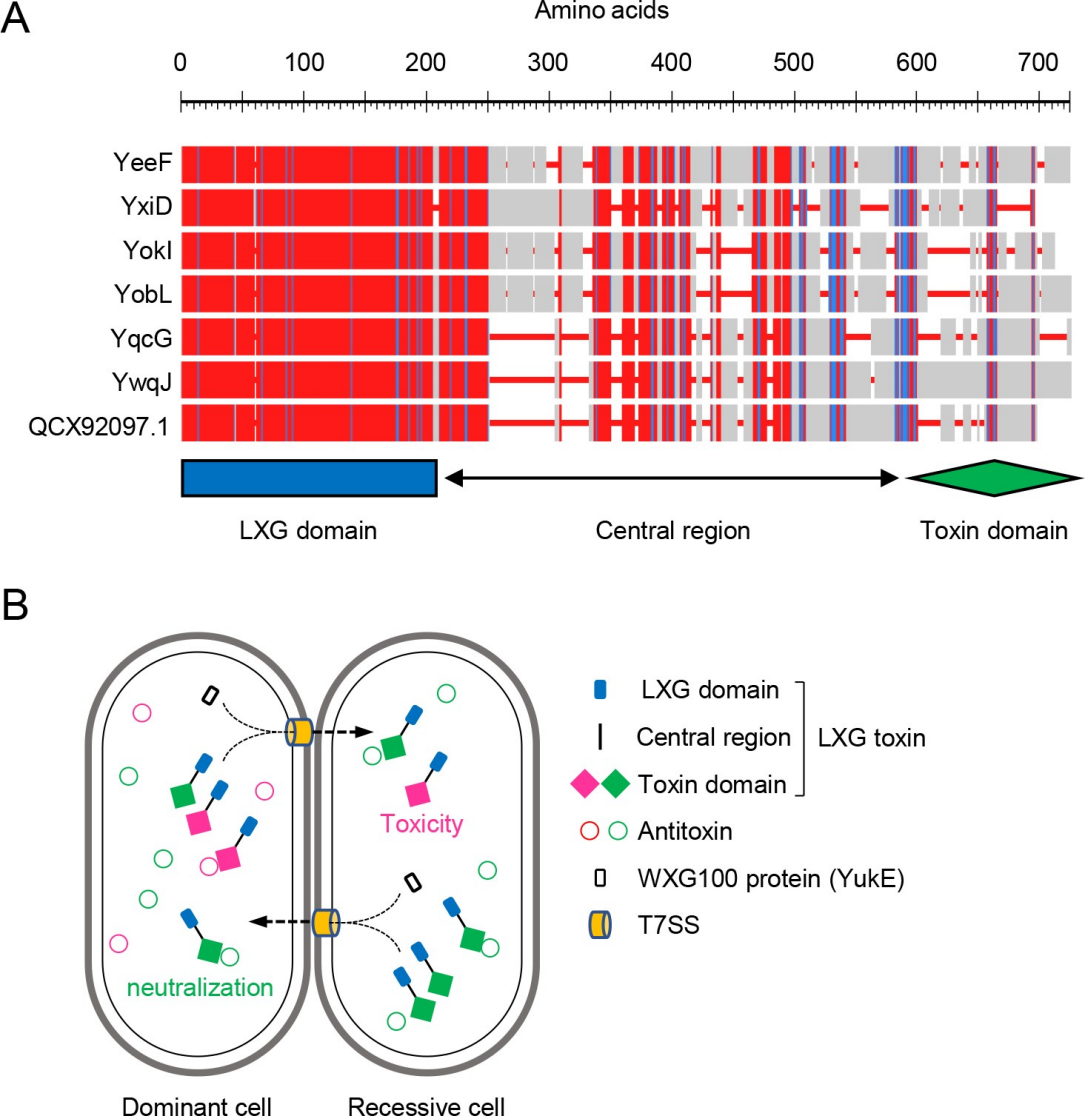
LXG toxins-antitoxin systems in *B*. *subtilis*. (A) Multiple alignment of LXG toxins from *B*. *subtilis* strain 3610. Alignment was constructed using NCBI COBALT (https://www.ncbi.nlm.nih.gov/tools/cobalt/re_cobalt.cgi) with default settings. Red indicates highly conserved positions and blue indicates lower conservation. Red lines are gaps in alignment. The location of the LXG domain, the central region, and the toxin domain are shown below the alignment. (B) Intercellular competition mediated by LXG toxin-antitoxin systems. LXG toxins are neutralized by cognate antitoxins in toxin producers. The WXG100 protein promotes the T7SS-dependent delivery of LXG toxins from producers to recipients. If recipients do not express cognate antitoxins, delivered LXG toxins exert toxic effects. Cognate toxin domains and antitoxins are shown by the same color.

Transcription of the *yitPOM* operon (encoding the YIT toxin) is induced by the two-component regulatory system DegS-DegU during biofilm formation [26]. In addition to the *yitPOM* operon, previous transcriptome analyses showed that DegS-DegU induces several antibiotic and toxin genes [39–42]. We hypothesize that some of these toxins may function in biofilms like the YIT toxin and serve as biofilm-specific competition mechanisms. Among them, the *yeeF-yezG*, *yqcG-yqcF*, *ywqJ-ywqK*, and *yxiD-yxxE* pairs all encode putative polymorphic toxins and antitoxins. Although some of these toxin-antitoxin pairs were analyzed in *Escherichia coli* [43,44], their biological functions in *B*. *subtilis* remain unclear. These toxins belong to the LXG toxin family, which shares N-terminal LXG domains, variable central regions, and diverse C-terminal toxin domains (Fig 1A). LXG toxins are widely distributed in Firmicutes [45]. In *Streptococcus intermedius*, LXG toxins functioned as CDI toxins and were delivered to adjacent cells by the type VII secretion system (T7SS), killing recipient cells [45]. T7SSs are also widely distributed in Firmicutes [46]. Although they have structural diversity, T7SSs commonly contain a membrane-bound ATPase of the FtsK/SpoIIIC family, which forms a secretion pore and probably energizes protein secretion [46]. Conserved T7SS substrates are small proteins (approximately 100 amino acid residues in length) of the WXG100 family [46]. WXG100 proteins adopt a four-helix bundle structure, and form homo- and heterodimers, which are secreted by T7SS [46,47]. The signal sequence for T7 secretion was identified at the C-terminus of the WXG100 protein [47]. The WXG100 domain is structurally similar to the LXG domain and was proposed to mediate the secretion of LXG toxins through T7SSs [17] (Fig 1B). Indeed, WXG100 proteins bound to LXG domains and promoted export of LXG toxins through T7SS in *Streptococcus intermedius* [45]. However, since a needle-like structure that could delivers toxins directly into recipient cells has not been identified in T7SSs, it remains unclear how T7SSs deliver LXG toxins to recipients. In *B*. *subtilis*, T7SS is encoded by the *yukE* operon, in which the first and fourth genes encode a WXG100 family protein and a FtsK/SpoIIIC family protein, respectively. Like putative LXG toxin genes, transcription of the *yukE* operon is activated by the two-component regulatory system DegS-DegU [48,49]. We therefore hypothesize that putative LXG toxins and T7SS together may play a role in interbacterial competition in biofilms.

Here we address this hypothesis and investigate the function of the LXG toxin–antitoxin systems in *B*. *subtilis*. We show that *B*. *subtilis* strains have multiple diverse LXG toxin systems that are sufficient for the discrimination of self from nonself. In addition, LXG toxins specifically mediated intraspecies competition in biofilms. We also show that *B*. *subtilis* uses different types of polymorphic toxins, dependent on the mode of growth.

## Results

### Distribution of LXG toxins and cognate antitoxins in *B*. *subtilis* strains

The *B*. *subtilis* undomesticated strain NCIB 3610 (hereafter referred to as 3610 or the wild-type strain) has been widely used in biofilm studies. This strain has seven potential LXG toxin genes; six (*yeeF*, *yobL*, *yokI*, *yqcG*, *ywqJ*, and *yxiD*) are on its chromosome, and one (QCX92097.1) is on the endogenous large plasmid pBS32. Three chromosomal genes, *yobL*, *yokI*, and *yqcG*, are on the prophage-like element 6, the SPβ prophage, and the phage-derived *sigK*-intervening (*skin*) element, respectively. These seven toxins, which are 531 to 669 amino acids in length, have N-terminal LXG domains (Pfam, PF04740; 202 amino acids) (Fig 1A), although the LXG domain sequence of YxiD is less similar to those of the other toxins (S1A Fig). These toxins have different C-terminal toxin domains. Their downstream genes encode putative antitoxins, which do not share significant similarity [43,44]. These observations suggest that each toxin–antitoxin pair may function independently.

If LXG toxins and antitoxins mediate intraspecies competition, each *B*. *subtilis* strain may have different LXG toxin–antitoxin operons. To test this hypothesis, we analyzed the distribution of LXG toxin genes in 12 fully sequenced *B*. *subtili*s strains in addition to strain 3610. These comprised a variety of *B*. *subtilis* strains including four *B*. *subtilis* subspecies, *subtilis*, *natto*, *spizizenii*, and *globigii* (S2 Fig). To identify LXG toxin homologs in these strains, we conducted NCBI BLASTp search using the combined sequence of two distantly related LXG toxins, YeeF and YxiD, as a bait. In this search, we collected proteins with LXG domains and potential toxin domains, but excluded proteins with only LXG domains, truncated LXG domains, or no potential toxin domains. As a result, we identified 59 LXG toxin homologs in 12 strains, in addition to seven LXG toxins in strain 3610. Each of the 13 strains, including strain 3610, had three to nine LXG toxin homologs, with the majority of strains having five homologs. Phylogenetic and gene organization analyses revealed that these 66 LXG toxins were classified into 17 groups (S3 Fig and Table 1). Each group of proteins had highly similar LXG domains and central regions. Six groups were further classified into 20 subgroups based on differences in toxin domains. For example, ten YeeF homologs, encoded at the same position on each genome, were divided into four subgroups. Each protein in these subgroups had subgroup-specific substitutions at the C-terminus of the toxin domain but possessed a common DNA–RNA non-specific endonuclease motif (S4A Fig). YobL and YokI homologs, two subgroups of the YobL group, had very similar LXG domains and central regions, but completely different toxin domain sequences (S4B Fig). Likewise, each subgroup of the YqcG, YwqJ, and YxiD groups had different toxin domains (S4C and S4D Fig). Although the LXG toxin proteins in the YxiD and G groups were classified into different groups, these proteins probably have a common origin, because they were encoded at the same genomic position, and had almost the same N-terminal LXG domain sequence (S4E Fig). A similar observation was made for the YobL and C groups, in which proteins had quite similar N-terminal LXG domains (S4B Fig). Classification also revealed that *B*. *subtilis* strains had many LXG toxin proteins that were not found in strain 3610, which also formed several groups. In summary, 66 potential LXG toxins could be divided into 31 groups and subgroups (Table 1 and S1 Table). A motif search using the Pfam database revealed that toxin domains of 18 groups and subgroups contained known toxin motifs, 14 of which were nuclease motifs (S1 Table).

**Table 1:** Classification and distribution of LXG toxin and antitoxin genes in *B*. *subtilis* strains^a)^.

groups	YeeF				YobL		C	YqcG			YwqJ						YxiD		G			H	I	J	K	L	M	N	O	P	Q
**subgroups**	**1**	**2**	**3**	**4**	**1**	**2**		**1**	**2**	**3**	**1**	**2**	**3**	**4**	**5**	**6**	**1**	**2**	**1**	**2**	**3**										
*B.subtilis* subsp. *globigii* ATCC 49760					●											●										●					
*B.subtilis* subsp. *spizizenii* TU-B-10									●						●				**◯**			●					●				
*B.subtilis* subsp. *spizizenii* str. W23				●											●			●													
*B.subtilis* subsp. *subtilis* str. RO-NN-1			●				●		●					●						●											●
*B.subtilis* subsp. *natto* BEST195		●			●		●			●		●							**◯**				●	▲							
*B.subtilis* subsp. *subtilis* str. BAB-1			●		●																●								●	●	
*B.subtilis* HJ5			●		●							●									●								●		
*B.subtilis* XF-1			●		●								**◯**								**◯**								●		
*B.subtilis* TO-A	●				●			●					●				●								●						
*B.subtilis* subsp. *subtilis* str. NCIB 3610	●				●	●		●			●						●						●								
*B.subtilis* subsp. *subtilis* str. OH 131.1														●			●										●				
*B.subtilis* BSn5								●					●														●	●			
*B.subtilis* subsp. *subtilis* str. BSP1			●		●								●											●						●	

^a)^ ●, LXG toxin and antitoxin genes; ▲, 2 copies of LXG toxin and antitoxin genes; ◯, orphan LXG toxin genes. See S1 Table for detailed results.

We next investigated whether cognate antitoxins were as diverse as LXG toxins. Cognate antitoxins are usually encoded immediately downstream of toxin genes. In fact, many genes downstream of LXG toxins encoded proteins that contained typical antitoxin motifs, such as Pfam PF14567, PF15601, and PF18624 (S1 Table), although group G LXG toxin genes did not have downstream antitoxin genes. Phylogenetic analysis revealed that downstream antitoxins could be classified into 30 groups (S5 Fig). This classification pattern was fully consistent with that of LXG toxins; that is, antitoxins formed phylogenetic groups that corresponded to groups or subgroups of LXG toxins. Thus, with the exception of orphan group G LXG toxins, LXG toxins and antitoxins in 13 *B*. *subtilis* strains likely formed 30 specificity pairs (Table 1 and S1 Table). 13 *B*. *subtilis* strains possessed three to nine different LXG toxin–antitoxin pairs, which allowed for discrimination between these strains. The widespread distribution of diverse LXG toxins and antitoxins suggest that LXG toxin–antitoxin systems may play a role in intraspecies competition in *B*. *subtilis*.

Note that the number of LXG toxin groups and subgroups increases as the number of included *B*. *subtilis* strains increases. For example, we identified 823 LXG toxins by NCBI BLASTp search against *B*. *subtilis* (taxid, 1423), which includes 85 *B*. *subtilis* strains. Among these toxins, 79 were YeeF homologs, which could be classified into ten subgroups based on differences in toxin domains (S6 Fig).

### Expression of LXG toxins

**Fig 2:**
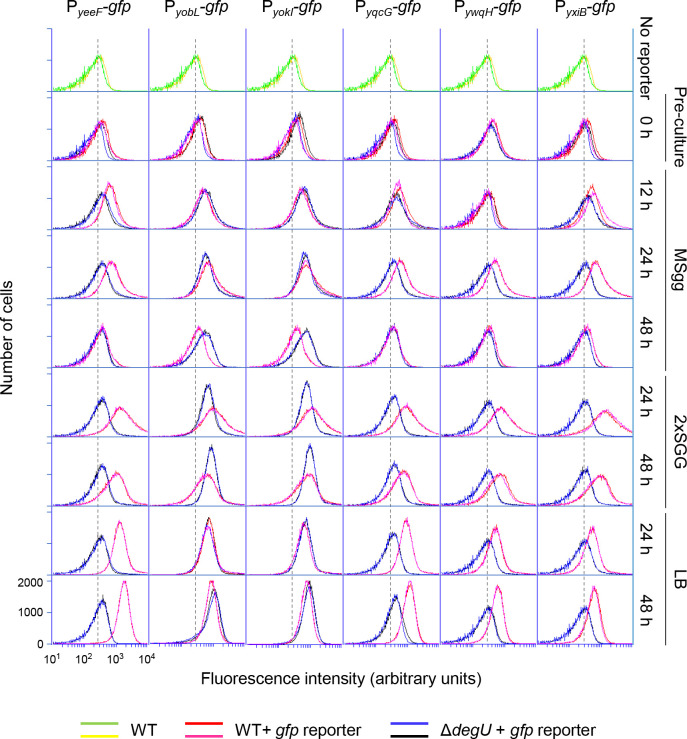
Expression of LXG toxin–antitoxin operons. Strains harboring promoter-*gfp* reporters were grown to an OD_600_ of 0.7 to 0.8 in liquid LB (preculture, 0 h). The cultures were diluted to an OD_600_ of 0.5, and 2 μl of the dilutions were spotted on three solid media, MSgg, 2×SGG, and LB. After 12 h, 24 h, or 48 h of incubation at 30°C, expression of *gfp* reporters in colonies were measured by flow cytometry. Strain 3610 (with no *gfp* reporter) was used as a negative control. Two sets of data are shown for each strain. All histograms are shown on the same scale, and values for the *x* and *y* axes are shown only in the lower left histogram. The peak positions of fluorescence from strain 3610 are indicated as a background reference by dotted lines.

To test whether LXG toxin-antitoxin systems are functional in *B*. *subtilis*, we analyzed the expression of six LXG toxin–antitoxin operons encoded on the chromosome of strain 3610. We fused the promoter regions of these operons to the promoterless green fluorescent protein (GFP) reporter and introduced the resultant promoter-*gfp* reporters into the 3610 chromosome. Expression of the *gfp* reporters in individual cells was measured by flow cytometry, in which the wild-type (with no *gfp* reporter) was used as a negative control. Wild-type and *gfp*-reporter strains were grown with vigorous shaking to an OD_600_ of 0.7–0.8 in the rich complex medium LB, which does not promote biofilm formation. At this stage, six reporters were not expressed, as these strains only exhibited as much fluorescence as the wild-type (Fig 2 preculture). These cultures were then spotted on three solid media, biofilm formation-promoting minimal medium MSgg [20], biofilm formation-promoting complex medium 2×SGG [50], and LB, and the expression of the *gfp* reporters in colonies was analyzed over 48 h. On MSgg medium, expression of the six reporters was induced at 12 h and 24 h and decreased at 48 h (Fig 2). On the rich medium 2×SGG, the six reporter strains expressed GFP at higher levels than on MSgg at 24 h, and exhibited extremely broad histogram peaks, indicating that a fraction of cells expressed GFP at very high levels. The expression of the reporters decreased at 48 h, as observed with MSgg. On another rich medium, LB, the six reporter strains also expressed more GFP than on MSgg. However, unlike on 2×SGG, the strains displayed sharp homogenous histogram peaks. These results indicate that *B*. *subtilis* expresses six LXG toxin–antitoxin operons on rich and minimal media likely in early stationary phase, and that some cells express these operons at quite high levels under nutrient-rich, biofilm-promoting conditions.

Since previous studies suggest that the *yeeF*, *yqcG*, *ywqH*, and *yxiB* operons are positively regulated by the two-component regulatory system DegS-DegU [39–42], we examined the effect of deleting *degU* on the reporters. The Δ*degU* mutation prevented expression of the P_*yeeF*_*-gfp*, P_*yqcG*_*-gfp*, P_*ywqH*_*-gfp*, and P_*yxiB*_*-gfp* reporters in three media, but not the P_*yobL*_*-gfp* or P_*yokI*_*-gfp* reporters (Fig 2). Thus, the six LXG toxin–antitoxin operons can be divided into two classes, DegSU dependent and DegSU independent.

### *In vivo* activity of LXG toxins and antitoxins

**Fig 3:**
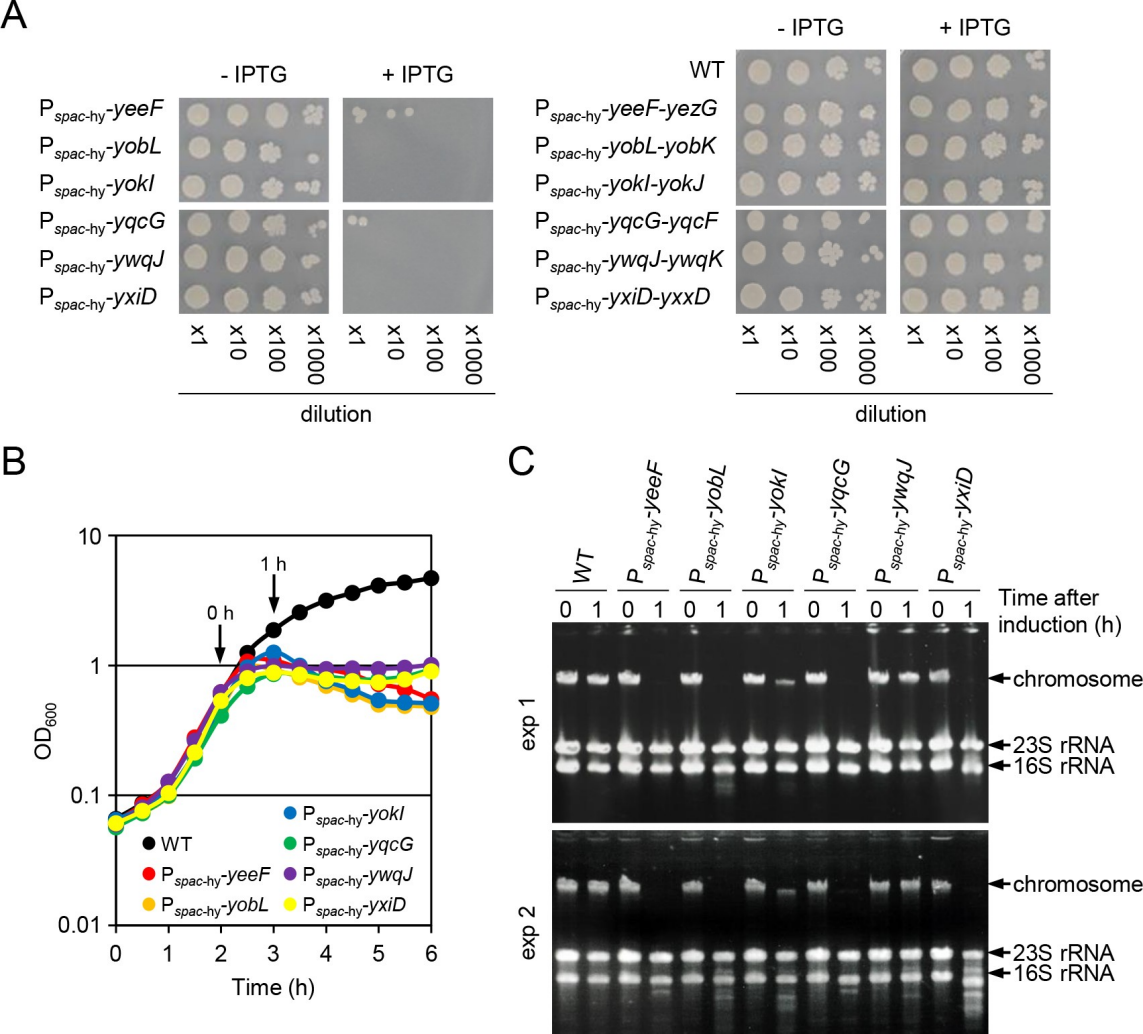
*In vivo* activity of LXG toxins and antitoxins. (A) Induction of LXG toxins prevents colony formation. Overnight cultures of P_*spac*-hy_-LXG toxin gene strains (left panels) and P_*spac*-hy_-LXG toxin-antitoxin operon strains (right panels) were serially diluted, and 2 μl of the dilutions was spotted onto LB solid media with or without 1 mM IPTG. The wild-type strain was used as a reference. The plates were then incubated at 37°C for 18 h. (B) Representative growth curves of wild-type (WT) and P_*spac*-hy_-LXG toxin gene strains. Strains were grown at 37°C in LB with vigorous shaking, and optical density at OD_600_ was monitored over time. IPTG (final concentration 1 mM) was added at the indicated time point (0 h). (C) The DNA and RNA content of cells before and after induction of LXG toxins. Cells were collected at 0 h and 1 h shown in panel B, samples of total DNA and RNA were isolated, and the samples from 0.03 OD cells were then analyzed by agarose gel electrophoresis. The positions of chromosome DNA and rRNAs are indicated by arrows. Results of two independent experiments are shown.

To determine whether six LXG toxin-antitoxin systems have biological activity in *B*. *subtilis*, we placed LXG toxin genes only or LXG toxin and antitoxin operons, under the control of the IPTG-inducible, LacI-repressible *spac*-hy promoter [51] at the *amyE* locus of the chromosome. The induction of any of the six LXG toxins strongly inhibited colony formation (Fig 3A left), whereas the induction of any of the six LXG toxin and antitoxin operons did not inhibit colony formation (Fig 3A right). These results show that these six pairs function as effective toxin-antitoxin systems. We tried to detect toxin activity. In shaking culture, induction of any of the six toxins resulted in immediate cessation of growth but did not lead to drastic cell lysis (Fig 3B). Since YeeF, YobL, YokI, and YqcG toxins contain nuclease motifs (S1 Table), we isolated chromosomal DNA and ribosomal RNA (rRNA) from cells before and 1 h after induction of LXG toxins. Induction of YeeF, YobL, YokI, YqcG, and YxiD caused a great decrease in chromosomal DNA (Fig 3C). To measure cellular DNA content, we stained cells with the DNA binding fluorescent dye, propidium iodide. Flow cytometry analysis showed that induction of YeeF, YobL, YokI, YqcG, and YxiD produced anucleate cells (S7 Fig). Although the C-terminal toxin domains of YobL, YokI, and YqcG functioned as RNases when expressed in *Escherichia coli* [43,44], these results indicate that YeeF, YobL, YokI, YqcG, and YxiD toxins function as DNases in *B*. *subtilis*. By contrast, induction of YwqJ had no effect on the chromosome or rRNA despite its marked effect on growth (Fig 3B and 3C). YwqJ, which has an unknown deaminase-like motif, may act on small molecules.

### LXG toxins mediate intercellular competition

**Fig 4:**
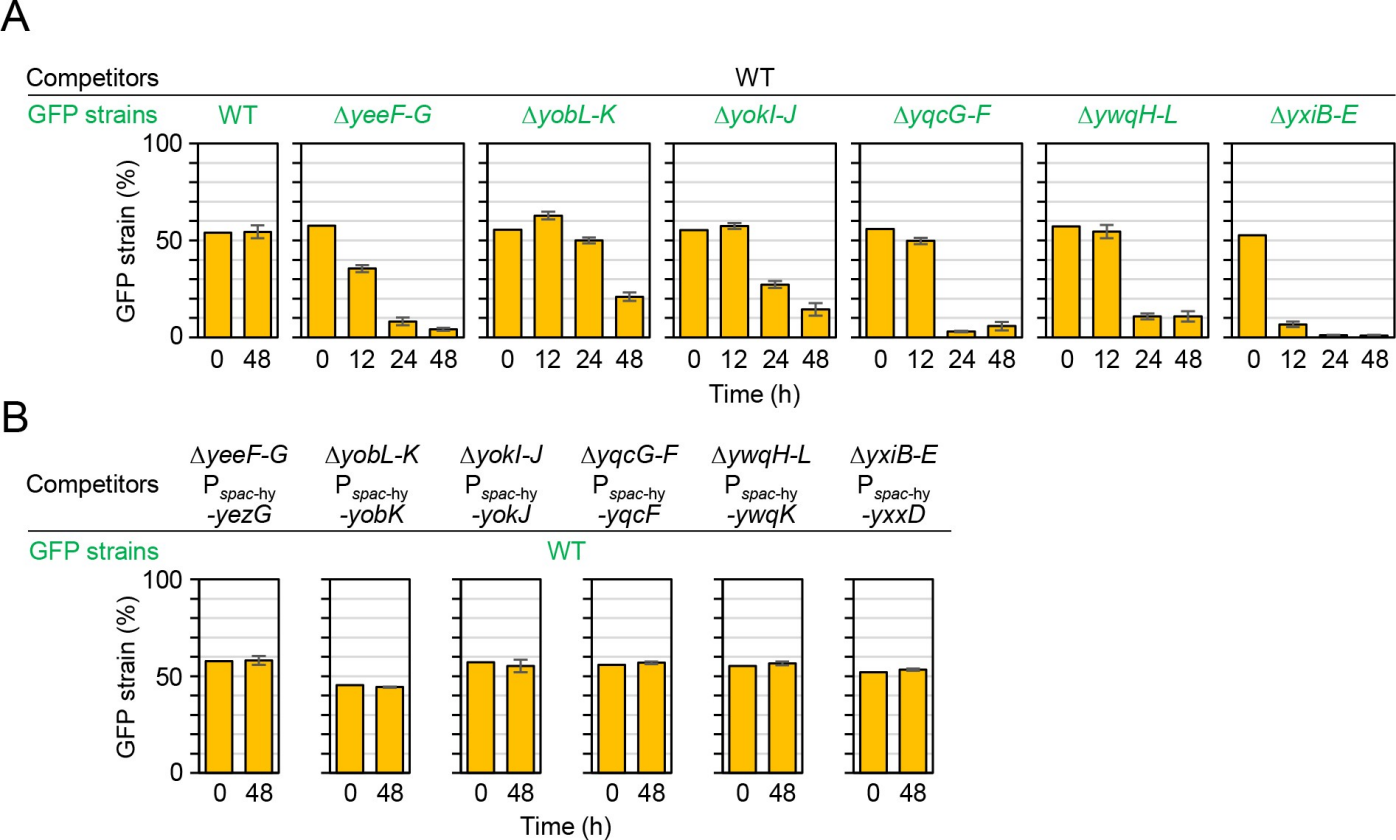
LXG toxins and antitoxins mediate intercellular competition. (A) Time-course analysis of competition. The wild-type strain and LXG toxin–antitoxin deletion mutants carrying a constitutively expressed *gfp* reporter (GFP strains) were grown to an OD_600_ of 0.7–0.8. and precisely diluted cultures (OD_600_ of 0.5) of the wild-type and one of the mutants were mixed at a 1:1 ratio. Exact proportions of the GFP strains in the mixtures were determined by flow cytometry and used as time 0 samples. The mixtures were spotted onto solid MSgg medium, and the inoculated plates were then incubated at 30°C. The proportion of GFP-reporter strains within colonies at each time point was determined by flow cytometry using three independent colonies. Percentages are presented as mean ± standard deviation (*n* = 3). (B) Inducing antitoxins restored competitiveness to toxin–antitoxin deletion mutants. Antitoxin genes were ectopically induced from the IPTG-dependent *spac*-hy promoter at the *amy* locus of mutant chromosomes. The indicated strains were co-cultured on MSgg supplemented with 0.1 mM IPTG. Percentages are presented as mean ± standard deviation (*n* = 3).

To test whether the six LXG toxin–antitoxin operons mediate intercellular competition, we constructed corresponding deletion mutants. The deletion mutants lacked toxin and antitoxin genes, or entire operons including toxin and antitoxin genes, namely, Δ*yeeF-yezG* (hereafter Δ*yeeF*–*G*), Δ*yobL-yobK* (Δ*yobL*–*K*), Δ*yokI-yokJ* (Δ*yokI*–*J*), Δ*yqeF-yqcG* (Δ*yqeF*–*G*), Δ*ywqH-ywqI-ywqJ-ywqK-ywqL (*Δ*ywqH*–*L*), and Δ*yxiB-yxiC-yxiD-yxxD-yxxE* (Δ*yxiB*–*E*). Deletion of these toxin and antitoxin genes had little or no effect on growth, because these mutants grew and developed colony biofilms (wrinkled colonies) in a manner comparable to the wild-type strain (S8 Fig). We carried out one-to-one competition assays between the deletion mutants and the wild-type strain. If LXG toxins function as intercellular toxins, then those produced by the wild-type strain should kill or suppress the deletion mutants that cannot produce the required antitoxin. To distinguish wild-type from mutant cells, we introduced a constitutively expressed *gfp* reporter into the chromosomes of the mutants. The wild-type strain and the GFP-labeled mutants were grown with vigorous shaking to an OD_600_ of 0.7–0.8 in LB. At this stage, cells did not express LXG toxin–antitoxin operons, as shown in Fig 2. Then diluted cultures (OD_600_ of 0.5) were mixed at a 1:1 ratio of wild-type strain to deletion mutant and spotted on solid MSgg medium. The initial cell density was 1.8 × 10^5^ cells per spot (diameter, 2~3 mm), on average (*n* = 8) (S1 Dataset). After inoculation, the proportion of GFP-labeled mutant cells in colonies was analyzed over 48 h by flow cytometry. The wild-type strain outcompeted all six mutants by 48 h at different speeds (Fig 4A). Specifically, the proportion of Δ*yxiB*–*E* mutant cells in colonies decreased to <10% by 12 h, whereas the proportion of Δ*yeeF*–*K*, Δ*yqcG*–*F*, and Δ*ywqH*–*L* mutants decreased to ~10% by 24 h. The proportion of Δ*yobL*–*K* and Δ*yokI*–*J* mutants gradually decreased until 48 h. No such change in proportion was observed in the co-culture of the wild-type and the wild-type with the *gfp* reporter. The fitness defect of these mutants was rescued by ectopically expressing antitoxins from the IPTG-inducible *spac*-hy promoter on the chromosomes (Fig 4B). These results demonstrate that six LXG toxin-antitoxin systems mediate intercellular competition.

### T7SS is required for LXG toxin delivery

**Fig 5:**
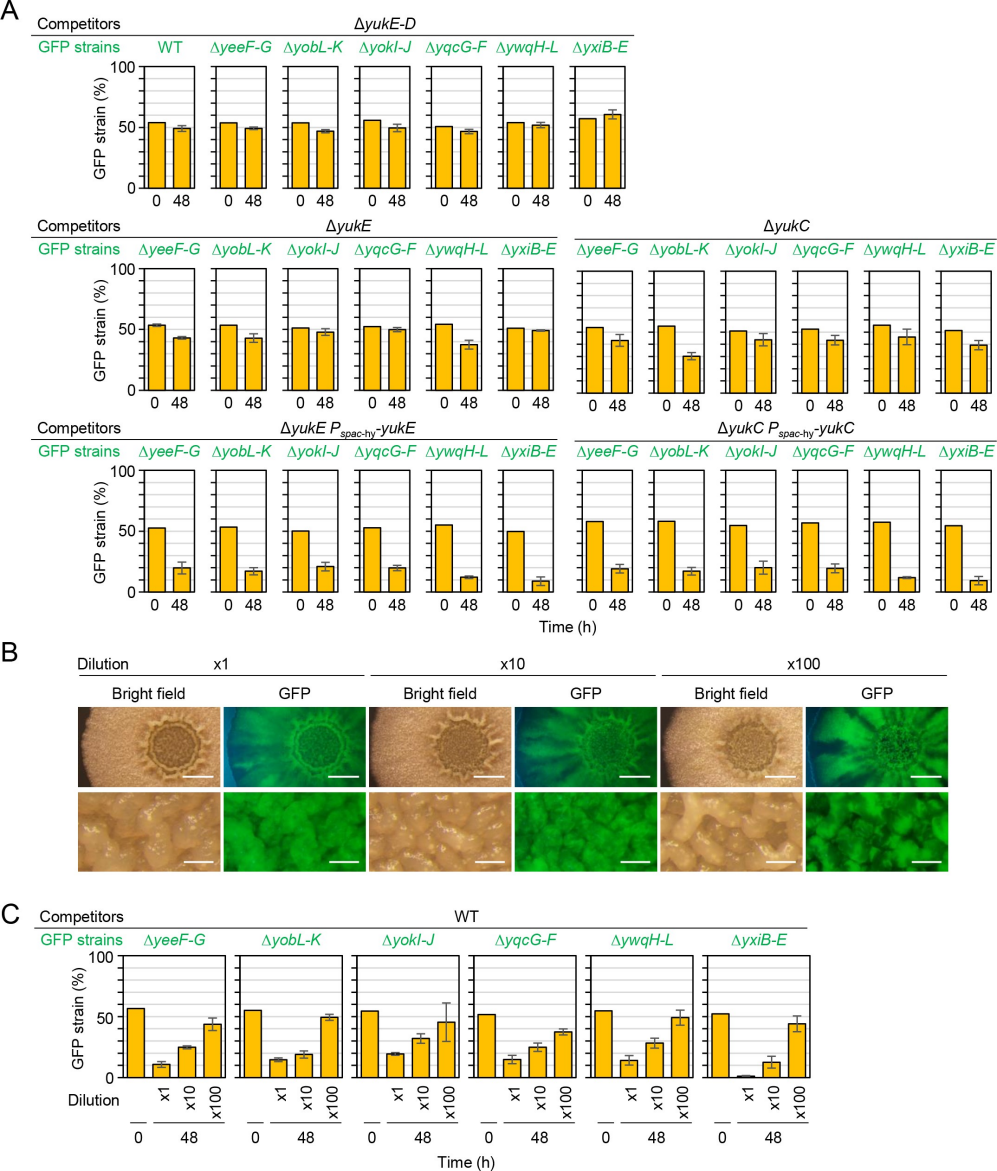
T7SS is required for LXG toxin delivery. (A) Competition assays between LXG toxin–antitoxin deletion mutants and T7SS mutants. Strains were co-cultured for 48 h on MSgg. Competition assays using Δ*yukE* P_*spac*-hy_-*yukE* and Δ*yukC* P_*spac*-hy_-*yukC* mutants were done on MSgg supplemented with 0.1 mM IPTG. (B) Reducing the initial cell density induced spatially segregated growth. Cultures (OD_600_ of 0.5) of the wild-type with or without the *gfp* reporter were mixed at a 1:1 ratio. The mixtures were diluted 1 to 100-fold, and 2 μl of the diluted mixtures were spotted on MSgg solid medium. After 48 h, colonies were observed with a stereomicroscope. Top views are colony images, and bottom views are enlarged images of the colony center. Scale bars, 2 mm (top), and 0.2 mm (bottom). (C) Cluster formation prevented LXG toxin function. Competition assays were conducted using diluted inoculation mixtures. Percentages are presented as mean ± standard deviation (*n* = 3).

In *S*. *intermedius*, LXG toxins functioned as contact-dependent inhibition (CDI) toxins [45]. *S*. *intermedius* has three LXG toxin genes, each of which forms an operon with cognate antitoxin and WXG100 protein genes [45]. WXG100 proteins bound to cognate LXG toxins and promoted their export through the type VII secretion system (T7SS) [45]. In *B*. *subtilis*, LXG toxin-antitoxin operons do not contain WXG100 protein genes. The WXG100 protein (YukE) and T7SS are encoded by the seven-gene operon *yukEDCB-yueBCD* in *B*. *subtilis* [48]. If WXG100 and T7SS are required for LXG toxin delivery in *B*. *subtilis*, then a deletion mutant of the entire *yukE* operon (Δ*yukE*–*D*) should not cause LXG toxin-dependent toxicity. One-to-one competition assays showed that, unlike the wild-type, the Δ*yukE*–*D* did not outcompete any LXG toxin–antitoxin deletion mutant (Fig 5A top). The Δ*yukE*–*D* mutant itself had no fitness defect, as it performed as well as the wild-type in competition assays. These results indicate that the Δ*yukE*–*D* mutation prevents toxin delivery without affecting antitoxin production. To distinguish effects of WXG100 and T7SS on toxin delivery, we constructed markerless in-frame deletion mutants of *yukE* and *yukC* and tested their competitiveness. One-to-one competition assays showed that both the WXG100 mutant Δ*yukE* and the T7SS mutant Δ*yukC* did not outcompete any LXG toxin–antitoxin deletion mutant (Fig 5A middle). Ectopic expression of *yukE* or *yukC* complemented corresponding deletion mutations as the complemented strains outcompeted LXG toxin–antitoxin deletion mutants (Fig 5A bottom). These results indicate that six LXG toxins are delivered to target bacterial cells in a YukE (WXG100 protein) and T7SS-dependent fashion in *B*. *subtilis*. Delivery of three LXG toxins were promoted by different WXG100 proteins in *S*. *intermedius* [45], whereas delivery of six LXG toxins were probably promoted by a single WXG100 protein, YukE in *B*. *subtilis*. This difference is probably because unlike LXG toxins of *B*. *subtilis*, LXG domains of *S*. *intermedius* LXG toxins are less similar to each other (S1B Fig). Expression of the *yukE* operon is positively regulated by the DegS-DegU two-component system [49]. Although two LXG toxin–antitoxin operons were expressed independently of DegS-DegU, these observations suggest that all six LXG toxin–antitoxin systems are directly or indirectly regulated by DegS-DegU.

LXG toxins are expected to function as CDI toxins and to be delivered into the periplasm or cytoplasm of recipient cells [45]. Unlike diffusible toxins, CDI toxins require cell–cell contact to exert their effect [52]. However, some CDI toxin producers cannot kill or suppress competitors if competitors form clusters before encountering toxin producers [53,54]. This phenomenon, known as herd protection, is caused by the accumulation of dead cells at interstrain boundaries. Accumulated dead cells form physical barriers and prevents CDI toxin producers from contacting new competitor cells. To determine whether LXG toxins function as CDI toxins, we tested the effect of cluster formation on the efficacy of LXG toxins. First, we tested whether reducing initial cell density allowed two strains to form separate clusters on solid medium before encountering one another, leading to spatially segregated growth in the resultant colonies. When a 1:1 mixture of the wild-type and the wild-type with the *gfp* reporter was inoculated on MSgg without dilution, the entire colony exhibited GFP fluorescence (Fig 5B 1× top), indicating that the two strains grew together. The contrast between GFP-high and GFP-low regions became clearer as the inoculation mixtures were diluted. Diluting the inoculation mixture 100-fold allowed the two strains to clearly form strong and weak GFP-fluorescent sections radially in peripheral areas of the colony, which expanded outward (Fig 5B 100× top). Enlarged images showed that strong and weak GFP-fluorescent sections also had a patchy distribution in wrinkle structures in central regions of the colony, which expanded upward (Fig 5B 100× bottom). Thus, reducing initial cell density drove spatially segregated growth in colonies. We then carried out competition assays using 10-fold and 100-fold diluted mixtures of the wild-type and LXG toxin–antitoxin deletion mutants. Reducing initial cell density significantly weakened the effect of LXG toxins, especially at the 100-fold dilution, in which the six LXG toxin–antitoxin deletion mutants did not exhibit significant fitness defects compared with the wild-type (Fig 5C). These results indicate that herd protection is effective against LXG toxins, and thus LXG toxins probably function as CDI toxins.

### LXG toxins mediate competition specifically in biofilms

**Fig 6:**
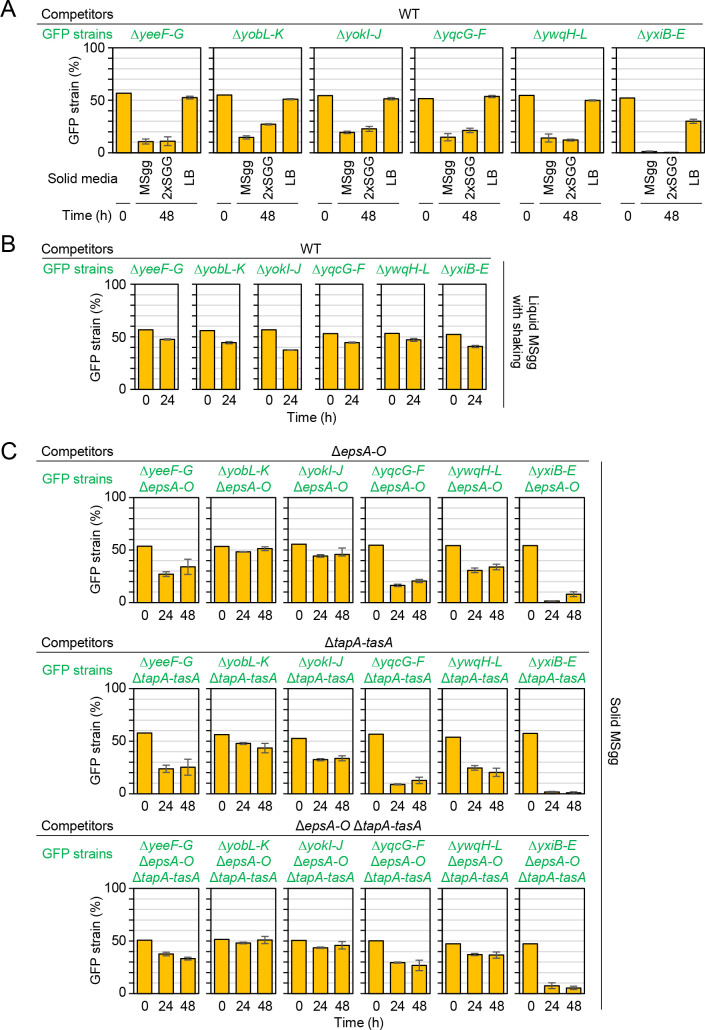
LXG toxin–antitoxin systems specifically function in biofilms. (A) Competition assays with the wild-type strain and LXG toxin–antitoxin deletion mutants on three different solid media. (B) Competition assays with the wild-type strain and LXG toxin–antitoxin deletion mutants in liquid shaking culture. Strains were co-cultured for 24 h in liquid MSgg medium with vigorous shaking. (C) Biofilm matrix polymers were required for LXG toxin function. Competition assays were conducted in the Δ*epsA*–*O* (top) or Δ*tapA*–*tasA* (bottom) mutant backgrounds. Percentages are presented as mean ± standard deviation (*n* = 3).

As described above, expression levels of LXG toxin–antitoxin operons differed depending on medium conditions. We therefore examined whether LXG toxins exerted different effects depending on medium conditions. On 2×SGG medium, the LXG toxin–antitoxin deletion mutants were outcompeted by the wild-type by 48 h, as observed on MSgg medium (Fig 6A). By contrast, these deletion mutants performed almost as well as the wild-type in competition assays on LB (Fig 6A), although LXG toxin–antitoxin systems were expressed in LB (Fig 2). Since unlike LB, MSgg and 2×SGG media promote biofilm formation, we hypothesized that LXG toxin systems might specifically work in biofilms. To test this hypothesis, we examined the effect of inhibiting biofilm formation on LXG toxin function in two experiments. First, we carried out competition assays under shaking conditions, which prevents biofilm formation. In liquid shaking culture with MSgg medium, all six mutants performed nearly as well as the wild-type in competition assays (Fig 6B). Second, we carried out competition assays in the Δ*epsA*–*O*, Δ*tapA*–*tasA*, and Δ*epsA*–*O* Δ*tapA*–*tasA* mutant backgrounds. Since the *epsA*–*O* and *tapA-tasA* operons are required for the synthesis of biofilm matrix polymers, exopolysaccharides and TasA amyloid polymers, respectively, deleting these operons abolishes biofilm formation [20,55–57]. In Δ*epsA*–*O*, Δ*tapA*–*tasA*, and Δ*epsA*–*O* Δ*tapA*–*tasA* backgrounds, the proportions of the toxin-antitoxin deletion mutants Δ*yeeF-G*, Δ*yobL-K*, Δ*yokI-J*, and Δ*ywqH-L* in colonies remained almost constant or decreased only mildly on MSgg (Fig 6C). The proportion of the Δ*yqcG-F* mutant in colonies decreased mildly only in the Δ*epsA*–*O* Δ*tapA*–*tasA* background. These results indicate that YeeF, YobL, YokI, YqcG, and YwqJ toxins require exopolysaccharides and TasA amyloid polymers for their full function. By contrast, the proportion of the Δ*yxiB-E* mutant remained very low in co-cultured colonies, even in the Δ*epsA*–*O* Δ*tapA*–*tasA* background. These observations indicate that the delivery of the YxiD toxin may require an unknown factor that is probabaly induced under biofilm conditions.

### Duels between toxin–antitoxin deletion mutants

**Fig 7:**
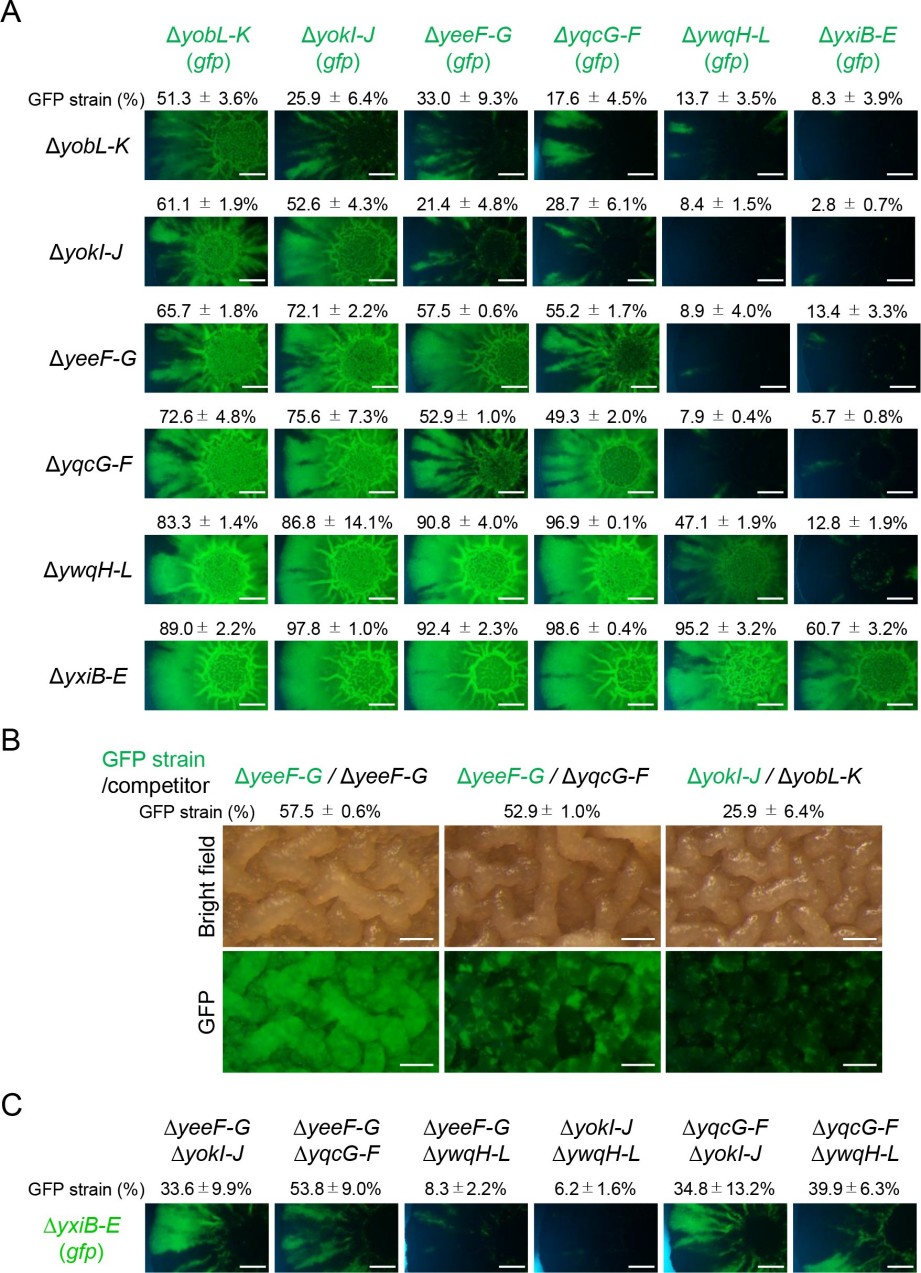
Round-robin duels between six LXG toxin–antitoxin deletion mutants. (A) The indicated LXG toxin–antitoxin deletion mutants, with or without the *gfp* reporter, were co-cultured at a 1:1 ratio on MSgg solid medium. After 48 h, the proportion of GFP-reporter strains within colonies was determined as mean ± standard deviation (*n* = 3). Fluorescent images of colonies are also shown. The experiments were performed at least three times, and representative examples are shown in the figures. The mutant strains are arranged from left (top) to right (bottom) in order of competitiveness. Scale bar, 2 mm. (B). Enlarged images of colony centers. The values of the GFP strain (%) are from panel A. Scale bar, 0.2 mm. (C) LXG toxins function synergistically. The Δ*yxiB*–*E* mutant with the *gfp* reporter was co-cultured with double LXG toxin–antitoxins mutants. The proportion of GFP-reporter strains within colonies was reported as mean ± standard deviation (*n* = 3). Fluorescent images of colonies are also shown. Scale bar, 2 mm.

Bioinformatic analysis predicted that each *B*. *subtilis* strain produces a different set of LXG toxins–antitoxins; thus, *B*. *subtilis* strains can attack each other, and simple predator–prey relationships are not expected in LXG toxin–antitoxin-mediated competition between *B*. *subtilis* strains. To mimic the situation, we carried out round-robin duels between the six LXG toxin–antitoxin deletion mutants, each of which was sensitive to one of the LXG toxins produced by the other deletion mutants. We spotted 1:1 mixtures of two LXG toxin–antitoxin deletion mutants (one of which was labeled with GFP) on MSgg solid medium. After 48 h of cultivation, the proportion of GFP-labeled strains in resultant colonies was analyzed by flow cytometry and fluorescent microscopy. The round-robin duels revealed a hierarchy of potency in six LXG toxins (Fig 7A). The YxiD and YwqJ toxins were the most and second most potent toxins, respectively. The YxiD-sensitive Δ*yxiB*–*E* mutant was overwhelmed by the other five mutants and appeared to be mostly absent in the colonies. Likewise, the YwqJ-sensitive Δ*ywqH*–*L* mutant was overwhelmed by the other four mutants, but not by the Δ*yxiB*–*E* mutant. Although the duels revealed that the order of the remaining four toxins was YqcG = YeeF > YokI > YobL, based on potency, duels between four mutants, Δ*yeeF-G*, Δ*yobL*–*K*, Δ*yokI*–*J*, and Δ*yqcG*–*F*, did not lead to definitive results. In these duels, the four mutants formed their own territories within colonies. For example, in duels with the Δ*yobL*–*K*, Δ*yokI*–*J*, or Δ*yqcG*–*F* mutant, the Δ*yeeF*–*G* (*gfp*) mutant, the weakest of the four, was proportionally reduced, but formed GFP-fluorescence-rich sections radially in peripheral regions of colonies (Fig 7A). Spatially segregated growth was observed within wrinkle structures in central regions of colonies (Fig 7B). The size of sections likely depended on the potency of LXG toxins (Fig 7A and 7B). Such section formation was rarely observed in duels between the same mutants, e.g., Δ*yeeF*–*G* versus Δ*yeeF*–*G* (*gfp*). These results indicate that LXG toxin–antitoxin systems enabled *B*. *subtilis* strains to outcompete competitors or to drive spatial segregation in communities. As described above, LXG toxin–antitoxin operons were highly expressed on 2×SGG medium. We therefore carried out the same duels on 2×SGG medium. However, the results were not much different from those observed on MSgg medium (S9 Fig).

*B*. *subtilis* strains produce multiple LXG toxins, which might synergistically inhibit competitors. To test this possibility, six LXG toxin–antitoxin double mutants were co-cultured with the weakest mutant Δ*yxiD*–*E* carrying *gfp*. As shown in Fig 7A, the Δ*yxiD*–*E* (*gfp*) mutant almost disappeared from colonies when competing against single deletion mutants of other LXG toxin–antitoxin operons. By contrast, the Δ*yxiD*–*E* (*gfp*) mutant formed separate sectors in colonies in duels with four double mutants, Δ*yeeF*–*G* Δ*yokI*–*J*, Δ*yeeF* Δ*yqcG*–*F*, Δ*yqcG*–*F* Δ*yokI*–*J*, or Δ*yqcG*–*F* Δ*ywqH*–*L* (Fig 7C). Thus, two LXG toxins produced by the Δ*yxiD*–*E* (*gfp*) mutant can compete with the most potent toxin YxiD produced by the four double mutants. These results indicate that producing multiple LXG toxins increases the chance of strains expanding their territories and surviving within communities, even if competitors produce potent LXG toxins.

### LXG toxins are effective against natural isolates

**Fig 8:**
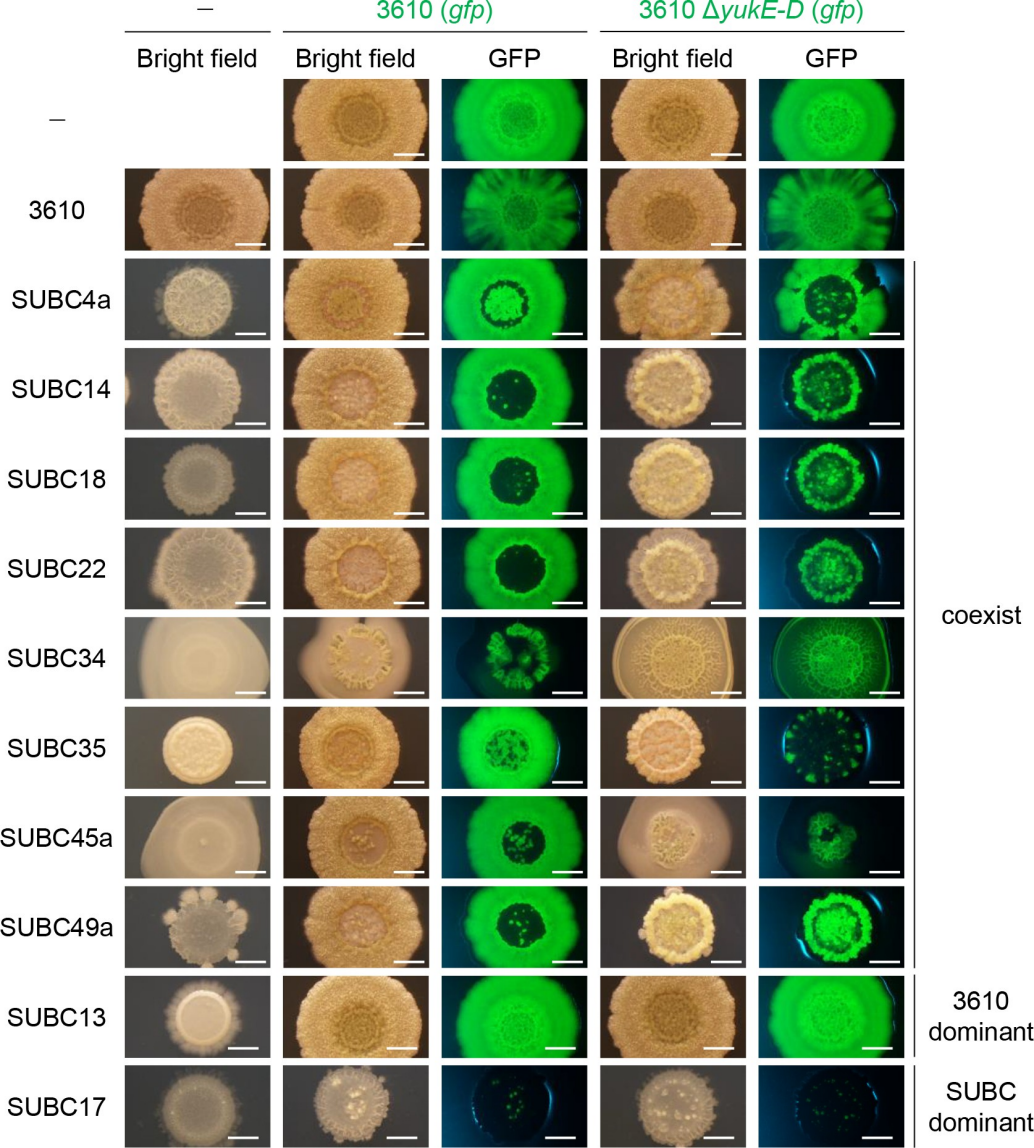
LXG toxins are effective against natural isolates of *B*. *subtilis*. The GFP-labeled strain 3610 or the T7SS mutant Δ*yukE*–*D* was co-cultured with a *B*. *subtilis* natural isolate at a ratio of 1:1 on MSgg medium. After 48 h of cultivation, the morphology and GFP fluorescence of colonies were observed with a stereomicroscope. Single-strain cultures are shown as references. Selected results are shown; see S10 Fig for complete results. The experiments were repeated twice and confirmed the reproducibility. The classification of co-cultured colonies is indicated on the right. Scale bar, 2 mm.

Natural strains of *B*. *subtilis* probably produce a different set of LXG toxins than NCIB3610 strain. To further investigate whether LXG toxin–antitoxin systems mediate intraspecies competition, we carried out competition assays using 26 natural isolates of *B*. *subtilis*, which were previously isolated from soil [58]. Specifically, the GFP-labeled 3610 strain or the T7SS mutant Δ*yukE*–*D* was mixed equally with one of 26 natural isolates, and the mixtures were inoculated on MSgg solid medium. If LXG toxins play a critical role in intraspecies competition, then the Δ*yukE*–*D* mutant, which cannot deliver LXG toxins to competitors, should not be as competitive as the wild-type strain. Co-culturing the GFP-labeled strain 3610 with natural isolates revealed that these strains very frequently grew by excluding one another (Figs 8 and S10). Specifically, either strain 3610 or the natural isolates became dominant in 18 resultant colonies, as these colonies exhibited either as much GFP fluorescence as those of GFP-labeled strain 3610 alone (3610 dominant, see SUBC13 and 3610 (*gfp*) coculture in Fig 8) or only faint GFP fluorescence (SUBC dominant, see SUBC17 and 3610 (*gfp*) coculture in Fig 8). These colonies were morphologically quite similar to either those of strain 3610 alone or the natural isolates alone. In eight other co-culture combinations, strain 3610 and the natural isolates coexisted, but were spatially segregated (Fig 8). Co-culturing the T7SS mutant Δ*yukE*–*D* with the eight natural isolates that coexisted with strain 3610 produced colonies that were morphologically different from co-cultured colonies of strain 3610 and the natural isolates. In most of the colonies, the Δ*yukE*–*D* mutant lost territory (bright GFP area) as compared with the territory of strain 3610 in corresponding colonies (Fig 8). The Δ*yukE*–*D* mutant grew with less spatial segregation in co-cultures with SUBC34 than did strain 3610. We could not determine exact cellular ratios in these colonies because these natural strains formed long chains of cells and/or colonies that strongly adhered to agar media. These results demonstrate that LXG toxins play a definite role at least in some intraspecies competition. Phylogenetic analysis of the natural isolates using partial sequences of *gyrA* showed that the eight strains that were sensitive to LXG toxins produced by strain 3610 belonged to two clades (S11 Fig). The importance of LXG toxins in intercellular competition is likely influenced by phylogenetic relationships between strains.

### The division of labor between antibiotics and toxins

*B*. *subtilis* secretes many antibiotics and toxins [7]. A previous study suggests that each *B*. *subtilis* strain uses a unique combination of these antimicrobials to discriminate kin from nonkin under swarming motility conditions [8]. This is likely why LXG toxins did not always play a definitive role in intraspecies competition in biofilms; that is, antibiotics and toxins other than LXG toxins probably played an important role under certain competition conditions. To compare the importance of other antimicrobials with LXG toxins in intraspecies competition, we analyzed the distribution of biosynthetic genes for 16 known and putative secreted antibiotics and toxins in 13 *B*. *subtilis* strains (Table 2). These antimicrobials were the Rhs family of polymorphic toxin (WapA), protein-derived peptide toxins (SDP, YIT, SDP3, SDP4, and SKF), lantibiotics (sublancin, subtilosin, subtilin, and subtilomycin), non-ribosomally synthesized peptide and polyketide antibiotics (surfactin, plipastatin, mycosubtilin, bacilycin, and bacillaene), and an aminosugar antibiotic (kanosamine) [7,23–26,59,60]. Note that these 16 antibiotics and toxins probably do not represent all the antimicrobials secreted by the 13 strains, and unknown antimicrobials likely exist. These antimicrobial biosynthesis genes were unevenly distributed among the 13 strains and had some strain-to-strain variation as previously reported [8]. However, the strain variability of these antimicrobials was less prevalent than that of LXG toxins. In particular, subtilosin, surfactin, bacilycin, bacillaene, and kanosamine were well conserved in these *B*. *subtilis* strains, indicating that these antimicrobials mediate interspecies competition rather than intraspecies competition.

**Table 2:** Distribution of antibiotic and toxin biosynthesis genes in *B*. *subtilis* strains^a^^,^ ^b^^)^.

toxins/antibiotics	WapA				SDP	YIT	SDP3	SDP4	SKF	sublancin	subtilosin	subtilin	subtilomycin	surfactin	plipastatin	mycosubtilin	bacilycin	bacillaene	kanosamine
**subgroups**	1	2	3	4															
*B.subtilis* subsp. *globigii* ATCC 49760											●			●	●	●		●	
*B.subtilis* subsp. *spizizenii* TU-B-10		●									●	●		●		●	●	●	●
*B.subtilis* subsp. *spizizenii* str. W23			●								●	●		●		●	●	●	●
*B.subtilis* subsp. *subtilis* str. RO-NN-1		●									●			●	●		●	●	●
*B.subtilis* subsp. *natto* BEST195				●			●				●			●			●		●
*B.subtilis* subsp. *subtilis* str. BAB-1		●				●					●			◯	●		●	●	●
*B.subtilis* HJ5		●				●					●			●	●		●	●	●
*B.subtilis* XF-1		●				●					●			●	●		●	●	●
*B.subtilis* TO-A	●				●	●			●		●			●	●		●	●	●
*B.subtilis* subsp. *subtilis* str. NCIB 3610	●				●	●			●	●	●			●	●		●	●	●
*B.subtilis* subsp. *subtilis* str. OH 131.1	●					●		●			●		●	●	●		●	●	●
*B.subtilis* BSn5	●					●	●				●		●				●	●	●
*B.subtilis* subsp. *subtilis* str. BSP1					●	●			●		●				●		●	●	●

^a)^ ●, having complete sets of antibiotic and toxin biosynthesis genes; ◯, strain BAB-1 was reported to produce surfactin, but the current version of its genome sequence contains frameshifts in *srfAA* and *srfAB* [61].

^b)^ Antibiotic and toxin biosynthesis genes; WapA, *wapA*; SDP, *sdpABC*; YIT, *yitPOM*, SDP3, *sdpABC* homolog3, SDP4, *sdpABC* homolog4; SKF, *skfABCDEFGH*; sublancin, *sunAT-bdbA-yolJ-bdbB*; subtilosin, *albABCDEFG*; subtilin, *spaBTCIFEGRK*; subtilomycin, *subAPBCIT*; surfactin, *sufAA-AB-AC-AD*; plipastatin, *ppsABCDE*; mycosubtilin, *fenF-mycABC*; bacilycin, *bacABCDEFG*; bacillaene, *pksABCDEFGHIJLMNR*; and kanosamine, *ntdABC*.

**Fig 9:**
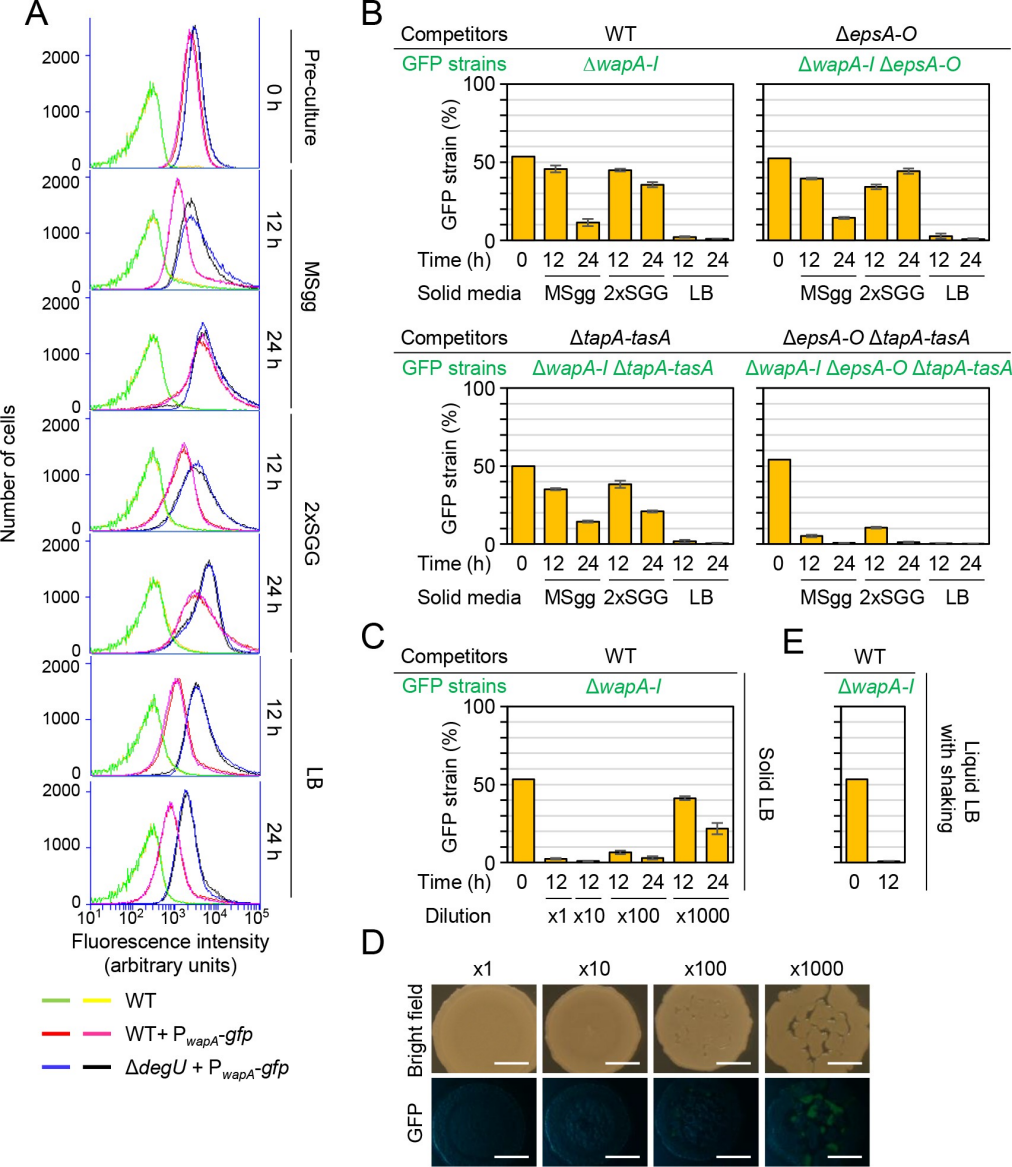
WapA exhibits different properties from LXG toxins. (A) Expression of the *wapAI* toxin-antitoxin operon. The P_*wapA*_*-gfp* strain was grown as described in Fig 1, and GFP fluorescence levels were measured by flow cytometry. Two sets of data are shown for each strain. (B) Competition assays with the wild-type strain and the Δ*wapAI* (*gfp*) mutant on MSgg, 2×SGG, and LB media in different genetic backgrounds. The proportion of GFP-reporter strains within colonies was reported as mean ± standard deviation (*n* = 3). (C) Herd protection did not protect against WapA. Competition assays were conducted on LB using diluted culture mixtures (*n* = 3). (D) Colony morphology in competition assays on LB. Colony morphology and GFP fluorescence were observed with a stereomicroscope 24 h after inoculation. (E) Competition assays with the wild-type strain and the Δ*wapAI* (*gfp*) mutant in LB under shaking conditions (*n* = 3).

Among these antimicrobial genes, another polymorphic toxin WapA markedly contributed to strain variability, and its four variants divided the 13 strains into five groups (four with one of the WapA variants and one with no WapA) (Table 2). WapA is a 2,334-amino acid CDI toxin composed of an N-terminal signal sequence, a central region containing RHS repeats, and a C-terminal tRNA nuclease domain [24]. Its toxic activity is neutralized by the WapI antitoxin encoded immediately downstream of *wapA* [24]. WapA is likely exported through the SecA-dependent secretion pathway and anchored on cell walls [62], but its exact mechanism of delivery to recipient cells remains unclear. We were interested in comparing WapA and LXG toxins. We analyzed the expression of the *wapAI* operon in three media using the P_*wapA*_*-gfp* reporter. When grown with vigorous shaking to an OD_600_ of 0.7–0.8 in liquid LB, the P_*wapA*_*-gfp*-reporter strain exhibited high levels of GFP fluorescence (Fig 9A 0 h). After inoculation onto MSgg, 2×SGG, and LB solid media, the P_*wapA*_*-gfp*-reporter strain also exhibited high levels of GFP fluorescence at 12 and 24 h, independent of the medium. The Δ*degU* mutation elevated expression from P_*wapA*_*-gfp*, as suggested previously [39–42]. These results indicate that *wapAI* was expressed during an earlier growth phase than were LXG toxin–antitoxin operons, and that DegS-DegU regulated *wapAI* and LXG toxin–antitoxin operons in an opposite fashion.

We hypothesized that WapAI and LXG toxin-antitoxin systems might function under different conditions. To test this hypothesis, we carried out one-to-one competition assays between the wild-type and the Δ*wapAI* mutant on three solid media. The proportion of the Δ*wapAI* mutant in mixed colonies decreased to <5% by 12 h on LB, whereas the proportion of the Δ*wapAI* mutant slowly decreased by or remained nearly constant at 24 h on MSgg and 2×SGG, respectively (Fig 9B). Thus, WapA had less of an effect in MSgg and 2×SGG media, which support biofilm formation. This effect was not due to decreased expression of *wapAI*, because *wapAI* was expressed in MSgg and 2×SGG at levels comparable to or higher than those in LB (Fig 9A). To test whether biofilm formation-associated mechanisms could influence WapA-mediated effects, we carried out the same competition assays in Δ*epsA*–*O*, Δ*tapA*–*tasA*, and Δ*epsA*–*O* Δ*tapA*–*tasA* backgrounds. Although the Δ*epsA*–*O* or Δ*tapA*–*tasA* mutation alone did not significantly affect the results obtained with the Δ*wapAI* mutant, in the Δ*epsA*–*O* Δ*tapA*–*tasA* background, the proportion of the Δ*wapAI* mutant significantly decreased by 12 h in all three media (Fig 9B). Considering the expression profiles of *wapAI*, these results indicate that WapAI mediates intercellular competition under non-biofilm conditions or before the onset of biofilm formation.

Comparison of the properties of WapA and LXG toxins that function under different conditions must help to understand LXG toxins. In competition assays, the Δ*wapAI* mutant was fully outcompeted by the wild-type on solid LB, even when the initial cell density was reduced by 100-fold (Fig 9C). Some Δ*wapAI* cells survived in mixed colonies when the initial cell density was reduced by 1,000-fold. However, in this case, the initial cell density was so low that cells in a spot formed multiple small colonies; nevertheless, the Δ*wapAI* mutant disappeared from outer areas of the spot where small colonies merged together, and the proportion of the Δ*wapAI* mutant in colonies decreased over time (Fig 9C and 9D). Thus, herd protection was unlikely to protect cells from WapA. Moreover, when wild-type and *wapAI* mutant strains were co-cultured in liquid LB with vigorous shaking, the Δ*wapAI* mutant was outcompeted by the wild-type. Thus, unlike LXG toxins, WapA mediates competition under shaking culture conditions (Fig 9E). Taken together, these results indicate that WapAI and LXG toxin-antitoxin systems have different properties. *B*. *subtilis* uses different CDI toxin-antitoxin systems for competition depending on the mode of growth.

## Discussion

Polymorphic CDI toxins play a major role in interbacterial competition between related strains under non-biofilm conditions. However, since the activity of many antibiotics and toxins including CDI toxins is inhibited in biofilms [29–38], their function in biofilms remains unclear. Here, we show that LXG toxin–antitoxin systems specifically mediated intraspecies competition in biofilms probably as CDI toxin-antitoxin systems. By contrast, another CDI toxin system WapAI mediated intraspecies competition only in non-biofilm conditions. These results strongly suggest that *B*. *subtilis* produces special CDI toxins that can mediate interbacterial competition in a unique biofilm environment.

*B*. *subtilis* strains have diverse LXG toxins, the number and variation of which is sufficient to distinguish each strain. Thus, *B*. *subtilis* strains can attack each other, and LXG toxin–antitoxin systems probably mediate mutual antagonism between *B*. *subtilis* strains (Fig 1B). LXG toxins were probably delivered to competitors by T7SS in *B*. *subtilis*, as observed in *S*. *intermedius* [45]. However, there are differences in the LXG toxin delivery mechanism of two bacteria. *S*. *intermedius* has three LXG toxin genes, each of which forms an operon with cognate antitoxin and WXG100 protein genes [45]. Delivery of three LXG toxins were promoted by different WXG100 proteins in *S*. *intermedius* [45]. By contrast, in *B*. *subtilis*, LXG toxin-antitoxin operons do not contain WXG100 protein genes, and the function of six LXG toxins depended on a single WXG100 protein, YukE in *B*. *subtilis*. This difference may be due to differences in LXG domains; that is, LXG domains of *S*. *intermedius* LXG toxins are less similar to each other, whereas LXG domains of *B*. *subtilis* LXG toxins are similar to each other (S1 Fig).

LXG toxins exerted their effects against competitors only in biofilm conditions. Unlike other CDI toxins including the WapA toxin, LXG toxins did not exert their effects in shaking cultures. LXG toxins of *S*. *intermedius* exerted their effect in solid medium but not liquid media [45]. These observations suggest that LXG toxins probably require prolonged cell contact for toxin delivery. The activity of five LXG toxins, YeeF, YobL, YokI, YqcG, and YwqJ, fully or partly depended on the presence of the *epsA* and *tapA* operons, which are required for synthesis of exopolysaccharides and TasA amyloid fibers [20,55–57]. In biofilms, *B*. *subtilis* cells form bundles of cell chains that are held together by exopolysaccharides and TasA amyloid fibers [20,55–57]. The requirement of exopolysaccharides and TasA amyloid fibers indicates that bundle formation is required for delivery of these LXG toxins. The close and nematic alignment of cells in bundles probably facilitates the delivery of toxins from producers to recipients. However, although Δ*epsA-O* and Δ*tapA-tasA* mutations prevent bundle formation, YeeF, YokI, YqcG and YwqJ toxins still exhibited partial toxicity in these mutation backgrounds, suggesting that exopolysaccharides and TasA amyloid fibers probably play another role in LXG toxin delivery. Unlike LXG toxins, the WapA toxin did not exert its effect in biofilms, and its action was impeded by the presence of exopolysaccharides and TasA polymers. These observations suggest that the close and nematic alignment of cells is not sufficient for the delivery of CDI toxins, and interaction between matrix polymers and toxins or toxin delivery apparatuses may greatly affect their delivery. These interactions probably prevent WapA delivery but facilitate the delivery of these five LXG toxins. By contrast, the activity of the YxiD toxin was not impeded by Δ*epsA-O* and Δ*tapA-tasA* mutations. However, YxiD toxin exerted its effect only in biofilm conditions. The delivery of the YxiD toxin may require an unknown biofilm-related extracellular factor. We showed that LXG toxins were delivered in a T7SS and YukE (WXG100)-dependent fashion as observed in *S*. *intermedius* [45]. However, our understanding of the delivery mechanism of these toxins remains limited, and further work is required to elucidate the delivery mechanism.

The findings that each *B*. *subtilis* strain possesses three to nine diverse LXG toxin-antitoxin systems suggest that kin discrimination in *B*. *subtilis* biofilms is highly exclusive as previously suggested [8,28]. This system confers advantages that could improve access to space and resources. Since close kin strains can be strong competitors and can potentially exploit more extracellular products effectively due to their genetic relatedness, a highly exclusive system could be a solution to protect biofilm cells against competition and social exploitation. In addition to kin discrimination, LXG toxin-antitoxin systems probably play another role. In co-culture experiments, strain 3610 and several natural isolates coexisted but were spatially segregated in colony biofilms. The T7SS mutation Δ*yukE-*D, which prevented LXG toxin delivery, changed the segregation patterns. These observations indicate that LXG toxins are also involved in spatial segregation in biofilms. Since attack provokes counterattack, and lysis of competitors can lead to the release of cellular toxins and harmful molecules [63–65], excess engagement in warfare to eliminate competitors is costly and risky, especially for biofilm cells fixed within a matrix and unable to relocate easily. Driving spatial segregation can be a solution to avoid excess warfare. Mutual antagonism between CDI toxin producers has been shown to result in such segregation [53,66,67]. However, in co-culture experiments of two strains, each of which produced a different LXG toxin, potent LXG toxin producers became dominant. Spatial segregation was observed only when two strains produced LXG toxins of comparable potency. Thus, producing one LXG toxin is not always sufficient to drive spatial segregation. We showed that some strains producing two relatively weak LXG toxins were able to compete with the strain producing the most potent YxiD toxin, and co-culturing these strains led to their spatially segregated growth. These observations indicate that producing many diverse LXG toxins, rather than one, may be important in driving spatial segregation in biofilms.

Spatial segregation is probably a result of the short-range properties of CDI toxins and herd protection. Despite differences in potency, all six LXG toxins were sensitive to hard protection. Herd protection is thought to occur as follows; when clusters of two different CDI toxin producers encounter, adjacent competitors attack one another. This causes the accumulation of dead cells at the interstrain boundary, which forms a barrier to block further attack from CDI toxin producers [53,66,67]. Considering this mechanism, leaving dead cells intact is likely effective for herd protection. We showed that induction of six LXG toxins caused immediate growth cessation but not rapid cell lysis. Thus, the attack of these six LXG toxins probably leaves dead cells intact, meaning that the LXG toxins provide herd protection. However, herd protection is not always effective against CDI toxins. CDI toxins that are potent and rapidly lyse cells can eliminate competitors even when they are in clusters [67]. We observed that herd protection was ineffective against WapA, which functioned in non-biofilm conditions, although its mechanism remains unclear. Herd protection-sensitive properties of LXG toxins may be important for biofilm-dwelling cells to avoid excess warfare and for promoting biofilm diversity.

LXG toxins may not be the only toxins that work in biofilms. *B*. *subtilis* produced two types of CDI toxins, depending on the growth mode. We showed that the DegS-DegU two-component system activated transcription of four LXG toxin–antitoxin operons, in addition to the *yukE* operon encoding T7SS [49], while it negatively regulated transcription of the *wapAI* operon. These observations suggest that the growth mode-dependent use of antimicrobials is controlled in part through transcriptional regulation by DegS-DegU. In addition to LXG toxin-antitoxin operons, DegS-DegU activates transcription of the *yitP* operon (YIT toxin production) and the *bacA* operon (bacilycin synthesis) and represses the *srfA* operon (surfactin synthesis) [26,68,69]. Repression of the *srfA* operon indirectly induces plipastatin synthesis [68]. These DegS-DegU-dependent antibiotics and toxins may contribute to biofilm-specific competition mechanisms. The distribution of these antibiotics and toxins in *B*. *subtilis* strains varied. Each of them likely protects *B*. *subtilis* from different competitors in biofilms.

## Methods

### Bacterial strains and culture conditions

*B*. *subtilis* strain NCIB 3610 and derivatives used in this study are listed in Table 3. The construction of *B*. *subtilis* mutants is described in S1 text. Primers used for strain construction are listed in S2 Table. Natural isolates of *B*. *subtilis* were kindly provided by H. Takamatsu and R. Kuwana, and their *gyrA* sequences were described previously [58]. *B*. *subtilis* strains were grown in MSgg (5 mM potassium phosphate (pH 7), 100 mM MOPS (pH 7), 2 mM MgCl_2_, 700 μM CaCl_2_, 50 μM MnCl_2_, 50 μM FeCl_3_, 1 μM ZnCl_2_, 2 μM thiamine, 0.5% (w/v) glycerol, 0.5% (w/v) glutamate, 50 μg/ml tryptophan) [20], 2×SGG (16 g/l nutrient broth (BD Difco, Franklin Lakes, NJ, USA), 0.2% (w/v) KCl, 2 mM MgSO_4_·7H_2_O, 1 mM Ca(NO_3_)_2_, 0.1 mM MnCl_2_, 1 μM FeSO_4_) [50], or LB (LB Lennox; BD Difco). *E*. *coli* strains HB101 and JM105 were used for the construction and maintenance of plasmids.

**Table 3:** *B*. *subtilis* strains used in this study.

Strains	Genotypes	References or construction^a^
NCIB3610	prototroph	[20]
N1920	*amyE*::P_*yeeF*_-*gfp* (*cat*)	This study
N1939	*amyE*::P_yeeF_-*gfp* (*cat*) Δ*degU*::*kan*	N345 (Δ*degU*::*kan*) [26] → N1920
N1921	*amyE*::P_*yobL*_-*gfp* (*cat*)	This study
N1940	*amyE*::P_*yobL*_-*gfp* (*cat*) Δ*degU*::*kan*	N345 (Δ*degU*::*kan*) [26] → N1921
N1922	*amyE*::P_*yokI*_-*gfp* (*cat*)	This study
N1941	*amyE*::P_*yokI*_-*gfp* (*cat*) Δ*degU*::*kan*	N345 (Δ*degU*::*kan*) [26] → N1922
N1923	*amyE*::P_*yqcG*_-*gfp* (*cat*)	This study
N1942	*amyE*::P_*yqcG*_-*gfp* (*cat*) Δ*degU*::*kan*	N345 (Δ*degU*::*kan*) [26] → N1923
N1917	*amyE*::P_*ywqH*_-*gfp* (*cat*)	This study
N1944	*amyE*::P_*ywqH*_-*gfp* (*cat*) Δ*degU*::*kan*	N345 (Δ*degU*::*kan*) [26] → N1917
N1918	*amyE*::P_*yxiB*_-*gfp* (*cat*)	This study
N1943	*amyE*::P_*yxiB*_-*gfp* (*cat*) Δ*degU*::*kan*	N345 (Δ*degU*::*kan*) [26] → N1918
N2302	*amyE*::P_*spac*-hy_-*yeeF* (*cat*)	This study
N2303	*amyE*::P_*spac*-hy_-*yobL* (*cat*)	This study
N2304	*amyE*::P_*spac*-hy_-*yokI* (*cat*)	This study
N2305	*amyE*::P_*spac*-hy_-*yqcG* (*cat*)	This study
N2306	*amyE*::P_*spac*-hy_-*ywqJ* (*cat*)	This study
N2307	*amyE*::P_*spac*-hy_-*yxiD* (*cat*)	This study
N2296	*amyE*::P_*spac*-hy_-*yeeF-yeeG* (*cat*)	This study
N2297	*amyE*::P_*spac*-hy_-*yobL-yobK* (*cat*)	This study
N2298	*amyE*::P_*spac*-hy_-*yokI-yokJ* (*cat*)	This study
N2299	*amyE*::P_*spac*-hy_-*yqcG-yqcF* (*cat*)	This study
N2300	*amyE*::P_*spac*-hy_-*ywqJ-ywqK* (*cat*)	This study
N2301	*amyE*::P_*spac*-hy_-*yxiD-yxxD* (*cat*)	This study
N1925	Δ*yeeF-G*::*cat*	This study
N1927	Δ*yobL-K*::*cat*	This study
N1926	Δ*yokI-J*::*cat*	This study
N1928	Δ*yqcG-F*::*cat*	This study
N1993	Δ*ywqH-L*::*cat*	W593 [39] →NCIB3610
N1994	Δ*yxiB-yxxE*::*cat*	W592 [39] →NCIB3610
N1789	*amyE*::P_*spac*-hy_-*gfp* (*cat*)	This study
N1957	*amyE*::P_*spac*-hy_-*gfp* (*tet*)	pCM::TC [70] → *amyE*::P_*spac*-hy_-*gfp* (*cat*)
N2030	Δ*yeeF-G*::*cat amyE*::P_*spac*-hy_-*gfp* (*tet*)	N1957 → N1925
N2031	Δ*yobL-K*::*cat amyE*::P_*spac*-hy_-*gfp* (*tet*)	N1957 → N1927
N1934	Δ*yokI-J*::*cat amyE*::P_*spac*-hy_-*gfp* (*tet*)	N1957 → N1926
N2033	Δ*yqcG-F*::*cat amyE*::P_*spac*-hy_-*gfp* (*tet*)	N1957 → N1928
N2034	Δ*ywqH-L*::*cat amyE*::P_*spac*-hy_-*gfp* (*tet*)	N1957 → N1993
N2035	Δ*yxiB-yxxE*::*cat amyE*::P_*spac*-hy_-*gfp* (*tet*)	N1957 → N1994
N2024	Δ*yeeF-G*::*cat amyE*::P_*spac*-hy_-*yezG* (*erm*)	This study
N2025	Δ*yobL-K*::*cat amyE*::P_*spac*-hy_-*yobK* (*erm*)	This study
N2074	Δ*yokI-J*::*cat amyE*::P_*spac*-hy_-*yokJ* (*erm*)	This study
N2027	Δ*yqcG-F*::*cat amyE*::P_*spac*-hy_-*yqcF* (*erm*)	This study
N2028	Δ*ywqH-L*::*cat amyE*::P_*spac*-hy_-*ywqK* (*erm*)	This study
N2029	Δ*yxiB-yxxE*::*cat amyE*::P_*spac*-hy_-*yxxD* (*erm*)	This study
N1448	Δ*yukE-yueD*::*cat*	W1102 [39] →NCIB3610
N1997	Δ*yukE-yueD*::*spc*	pCM::SP [70] → Δ*yukE-yueD*::*cat*
N2401	Δ*yukE* (in-frame deletion)	This study
N2402	Δ*yukC* (in-frame deletion)	This study
N2403	Δ*yukE amyE*::P_*spac*-hy_-*yukE* (*cat*)	This study
N2404	Δ*yukC amyE*::P_*spac*-hy_-*yukC* (*cat*)	This study
N355	Δ*epsA-O*::*spc*	[71]
N2068	Δ*yeeF-G*::*cat* Δ*epsA-O*::*spc amyE*::P_*spac*-hy_-*gfp* (*tet*)	N355 → N1925
N2069	Δ*yobL-K*::*cat* Δ*epsA-O*::*spc amyE*::P_*spac*-hy_-*gfp* (*tet*)	N355 → N1927
N2070	Δ*yokI-J*::*cat* Δ*epsA-O*::*spc amyE*::P_*spac*-hy_-*gfp* (*tet*)	N355 → N1926
N2071	Δ*yqcG-F*::*cat* Δ*epsA-O*::*spc amyE*::P_*spac*-hy_-*gfp* (*tet*)	N355 → N1928
N2072	Δ*ywqH-L*::*cat* Δ*epsA-O*::*spc amyE*::P_*spac*-hy_-*gfp* (*tet*)	N355 → N1993
N2073	Δ*yxiB-yxxE*::*cat* Δ*epsA-O*::*spc amyE*::P_*spac*-hy_-*gfp* (*tet*)	N355 → N1994
N11	Δ*tapA-tasA*::*erm*	[71]
N2053	Δ*yeeF-G*::*cat* Δ*tapA-tasA*::*erm amyE*::P_*spac*-hy_-*gfp* (*tet*)	N11 → N1925
N2054	Δ*yobL-K*::*cat* Δ*tapA-tasA*::*erm amyE*::P_*spac*-hy_-*gfp* (*tet*)	N11 → N1927
N2055	Δ*yokI-J*::*cat* Δ*tapA-tasA*::*erm amyE*::P_*spac*-hy_-*gfp* (*tet*)	N11 → N1926
N2056	Δ*yqcG-F*::*cat* Δ*tapA-tasA*::*erm amyE*::P_*spac*-hy_-*gfp* (*tet*)	N11 → N1928
N2057	Δ*ywqH-L*::*cat* Δ*tapA-tasA*::*erm amyE*::P_*spac*-hy_-*gfp* (*tet*)	N11 → N1993
N2058	Δ*yxiB-yxxE*::*cat* Δ*tapA-tasA*::*erm amyE*::P_*spac*-hy_-*gfp* (*tet*)	N11 → N1994
N364	Δ*epsA-O*::*spc* Δ*tapA-tasA*::*erm*	N11 → N355
N2059	Δ*yeeF-G*::*cat* Δ*epsA-O*::*spc* Δ*tapA-tasA*::*erm amyE*::P_*spac*-hy_-*gfp* (*tet*)	N11 → N2068
N2060	Δ*yobL-K*::*cat* Δ*epsA-O*::*spc* Δ*tapA-tasA*::*erm amyE*::P_*spac*-hy_-*gfp* (*tet*)	N11 → N2069
N2061	Δ*yokI-J*::*cat* Δ*epsA-O*::*spc* Δ*tapA-tasA*::*erm amyE*::P_*spac*-hy_-*gfp* (*tet*)	N11 → N2070
N2062	Δ*yqcG-F*::*cat* Δ*epsA-O*::*spc* Δ*tapA-tasA*::*erm amyE*::P_*spac*-hy_-*gfp* (*tet*)	N11 → N2071
N2063	Δ*ywqH-L*::*cat* Δ*epsA-O*::*spc* Δ*tapA-tasA*::*erm amyE*::P_*spac*-hy_-*gfp* (*tet*)	N11 → N2072
N2064	Δ*yxiB-yxxE*::*cat* Δ*epsA-O*::*spc* Δ*tapA-tasA*::*erm amyE*::P_*spac*-hy_-*gfp* (*tet*)	N11 → N2073
N1976	Δ*yeeF-G*::*erm*	pCM::EM [70] → Δ*yeeF-G*::*cat*
N1977	Δ*yokI-J*::*erm*	pCM::EM [70] → Δ*yokI-J*::*cat*
N1978	Δ*yqcG-F*::*erm*	pCM::EM [70] → Δ*yqcG-F*::*cat*
N2018	Δ*yeeF-G*::*erm* Δ*yokI-J*::*cat*	N1976 → N1926
N2019	Δ*yokI-J*::*cat* Δ*yqcG-F*::*erm*	N1977 → N1926
N2020	Δ*yeeF-G*::*erm* Δ*yqcG-F*::*cat*	N1976 → N1928
N2021	Δ*yeeF-G*::*erm* Δ*ywqH-L*::*cat*	N1976 → N1993
N2022	Δ*yokI-J*::*erm* Δ*ywqH-L*::*cat*	N1977 → N1993
N2023	Δ*yqcG-F*::*erm* Δ*ywqH-L*::*cat*	N1978 → N1993
N1998	Δ*yukE-yueD*::*spc amyE*::P_*spac*-hy_-*gfp* (*tet*)	N1957 → N1997
N2075	*amyE*::P_*wapA*_*-gfp* (*cat*)	This study
N2076	*amyE*::P_*wapA*_*-gfp* (*cat*) Δ*degU*::*kan*	Δ*degU*::*kan* [26] → N2075
N1953	Δ*wapAI*::*cat*	This study
N1958	Δ*wapAI*::*cat amyE*::P_*spac*-hy_-*gfp* (*tet*)	Δ*degU*::*kan* [26] → N1953
N2077	Δ*wapAI*::*cat* Δ*epsA-O*::*spc amyE*::P_*spac*-hy_-*gfp* (*tet*)	N355 → N1958
N2078	Δ*wapAI*::*cat* Δ*tapA-tasA*::*erm amyE*::P_*spac*-hy_-*gfp* (*tet*)	N11 → N1958
N2088	Δ*wapAI*::*cat* Δ*epsA-O*::*spc* Δ*tapA-tasA*::*erm amyE*::P_*spac*-hy_-*gfp* (*tet*)	N2077 → N2078

### Bioinformatic analyses

LXG toxin homologs in *B*. *subtilis* strains were identified by an NCBI BLASTp search (https://blast.ncbi.nlm.nih.gov/Blast.cgi) using strain-specific taxid numbers. In this search, the combined sequence of YeeF and YxiD was used as a bait. Phylogenetic trees of LXG toxins and antitoxins were constructed using Multiple Sequence Alignment by CLUSTALW (https://www.genome.jp/tools-bin/clustalw) with default settings. Multiple sequence alignments were constructed using Multiple Sequence Alignment by CLUSTALW, Clustal Omega (https://www.ebi.ac.uk/Tools/msa/clustalo/) or NCBI COBALT (https://www.ncbi.nlm.nih.gov/tools/cobalt/re_cobalt.cgi) with default settings. Gene organization was compared using SyntTax (https://archaea.i2bc.paris-saclay.fr/synttax/).

### DNA and RNA isolation

Cultures (2 ml) of *B*. *subtilis* strains grown overnight at 28°C in LB were added to 100 ml LB, and the strains were grown with shaking at 37°C. When the OD_600_ reached 0.7–0.8, IPTG (final 1 mM) was added to the cultures. Before and 1 h after the addition of IPTG, cells (5 OD) were pelleted in a 15 ml tube by centrifugation (5,800 × g for 2 min) and immediately frozen in liquid nitrogen. Thawed cells were dissolved in 1 ml LETS buffer (10 mM Tris-HCl (pH 8.0), 50 mM LiCl, 10 mM EDTA, 1% sodium dodecyl sulfate), and then 0.7 ml glass beads (diameter 0.35–0.5 mm) and 1 ml phenol-chloroform-isoamyl alcohol (25: 24: 1, pH 8.0) were added. After vortexing for 5 min, samples were centrifuged at 5,800 × g for 10 min at 4°C. 0.6 ml of aqueous phase were transferred to a 1.5 ml tube and then mixed with 0.6 ml of isopropanol. Samples were centrifugated at 17,400 × g for 20 min at 4°C, and then pellets were resuspended in 100 μl sterilized water. One μl of samples were analyzed by 1% agarose gel electrophoresis.

### Propidium iodide staining

1 h after induction of LXG toxins, 1.5 ml of cells were pelleted by centrifugation (17,400 × g for 2 min). Cells were resuspended in 300 μl 10 mM Tris-HCl (pH 7.6) and then mixed with 700 μl ethanol. To fix the cells, the suspensions were kept at 4°C overnight. 200 μl of the suspensions were centrifuged at 17,400 × g for 2 min. Cells were resuspended in 100 μl of PBS (1.37 mM NaCl, 81 mM Na_2_HPO_4_, 26.8 mM KCl, 14.7 mM KH_2_PO_4_) containing 0.1 μg/ml propidium iodide, and incubated for 20 min in the dark. Propidium iodide-stained cells were analyzed by flow cytometry.

### Competition assays

Cultures (150 μl) of *B*. *subtilis* strains grown overnight at 28°C in LB were added to 5 ml LB, and the strains were grown with shaking at 37°C until the OD_600_ reached 0.7–0.8. The cultures were then diluted to an OD_600_ of 0.5 with LB, and two culture dilutions (500 μl each) were mixed well by vortexing. Two microliters of the mixtures were spotted onto solid medium, and the remaining volume was used to determine the proportions of the two strains at time 0 by flow cytometry. The inoculated plates were incubated at 30°C. After 12 h, 24 h, or 48 h, colonies were harvested to determine the proportions of the two strains by flow cytometry. Two or three colonies were harvested for the 12 h samples, while one colony was harvested for the 24 h and 48 h samples. The proportions of the two strains at each time point were determined by averaging three experiments. Raw data is shown in S1 Dataset. For competition assays in liquid MSgg medium, the two-culture mixtures were prepared similarly, and 30 μl of the mixtures were added to 5 ml MSgg or LB. One ml of liquid cultures were harvested for flow cytometry analysis. The proportions of the two strains in 24 h cultures were also determined by averaging three experiments.

### Flow cytometry analysis

Colonies were scraped with inoculation loops and suspended in 300 μl PBS. After the suspension was well dispersed by repetitive pipetting, cells were pelleted by centrifugation at 17,400 g for 1 min. Likewise, cells of liquid cultures were pelleted by centrifugation at 17,400 g for 1 min. Then, cells were fixed with 4% paraformaldehyde for 7 min [72]. Prior to flow cytometry analysis, biofilm cells were subjected to mild sonication [72]. Single-cell fluorescence was measured with an Accuri C6 flow cytometer (BD Biosciences, Franklin Lakes, NJ, USA). The number of recorded events was 50,000. The threshold was set at 20,000 on FSC-H.

### Expression of GFP reporters

To measure the expression of GFP reporters, *B*. *subtilis* strains were grown using similar procedures as described for competition assays, except that two strains were not mixed. The expression of GFP reporters was measured by flow cytometry as described above.

### Microscopy

The expression of GFP reporters in *B*. *subtilis* colonies was analyzed with a SZX7 stereomicroscope (Olympus, Tokyo, Japan) equipped with an AdvanCam-E3Rs digital color camera (Advan Vision, Tokyo, Japan) as described previously [26]. Images were obtained and processed with AdvanView (Advan Vision) and Photoshop Elements (Adobe, San Jose, CA, USA). The experiments were performed at least three times, and representative examples are shown in the figures.

## Supporting information

S1 Fig Multiple alignments of LXG domains. (A) LXG toxins of *B*. *subtilis* strain 3610. Identical amino acid residues in three or more proteins are highlighted in yellow. (B) LXG toxins of *S*. *intermedius*. Identical amino acid residues in two or three proteins are highlighted in green. Sequences were aligned using CLUSTALW (https://www.genome.jp/tools-bin/clustalw).
(PDF)

S2 Fig Phylogenetic tree of *B*. *subtilis* strains. The phylogenetic tree is constructed based on full-length *gyrA* alignment. *B*. *licheniformis* DSM13 was used as an outgroup reference.
(PDF)

S3 Fig Phylogenetic tree of 66 LXG toxin homologs. Group and subgroup names are indicated at the right of the clades. The proteins of strain 3610 are shown in red.
(PDF)

S4 Fig Sequence alignment of LXG toxin homologs. Indicated proteins were aligned using the Clustal Omega website (https://www.ebi.ac.uk/Tools/msa/clustalo/). Identical (*) and conserved (: or.) amino acids are indicated below the alignment according to the Clustal Omega scheme. Each subgroup of proteins is shown in a different color. Alignments of YeeF group proteins (A), YobL and C group proteins (B), YqcG group proteins (C), YwqH group proteins (D), and YxiD and G group proteins (E). Positions of LXG and toxin domains in proteins from strain 3610 (A–D) or proteins from strain W23 (E) are indicated above the sequences.
(PDF)

S5 Fig Phylogenetic tree of putative antitoxins. Group and subgroup names are indicated on the right. The proteins of strain 3610 are shown in red.
(PDF)

S6 Fig YeeF homologs from 85 *B*. *subtilis* strains. (A) Phylogenetic tree of 79 YeeF homologs from *B*. *subtilis*. Seventy-nine YeeF homologs were identified by BLASTp (taxid, 1423) using YeeF as a bait. Possible subgroups are indicated by bars at the right of the clades. Clades containing YeeF-1, 2, 3, or 4 subgroup proteins are labeled. (B) Alignment of C-terminal regions of 79 YeeF toxin homologs. Subgroups are color-coded for ease of viewing.
(PDF)

S7 Fig Overexpression of YeeF, YobL, YokI, YqcG, and YxiD toxins produces anucleate cells. Cells collected at 1 h shown in Fig 3B were fixed in 70% ethanol. These cells were stained with the DNA-dye propidium iodide (PI), and cellular DNA content was analyzed by flow cytometry. Plots of unstained cells are shown as references.
(PDF)

S8 Fig Colony development of LXG toxin–antitoxin deletion mutants. Wild-type and mutant strains were grown on three solid media. Top–down views of colonies are shown. Magnified images of colonies were taken with a stereomicroscope. Scale bar, 2 mm.
(PDF)

S9 Fig Round-robin duels between six LXG toxin–antitoxin deletion mutants in rich biofilm-supporting medium. Indicated LXG toxin–antitoxin deletion mutants, with or without the *gfp* reporter, were co-cultured at a 1:1 ratio on 2×SGG solid medium as described in Fig 5. The proportion of GFP-reporter strains within colonies is indicated above fluorescent images of colonies. The experiments were repeated at least three times, and representative examples are shown in the figures. Percentages are presented as mean ± standard deviation (*n* = 3). Scale bar, 2 mm.
(PDF)

S10 Fig LXG toxins mediate intraspecies competition. The GFP-labeled strain 3610 or the Δ*yukE*–*D* mutant was co-cultured with one of 26 *B*. *subtilis* natural isolates at a ratio of 1:1 on MSgg medium. After 48 h of cultivation, the morphology and GFP fluorescence of colonies were observed with a stereomicroscope. Single-strain cultures are shown as references. The classification of co-cultured colonies is indicated on the right. The experiments were repeated twice and confirmed the reproducibility. Scale bar, 2 mm.
(PDF)

S11 Fig Phylogenetic tree of *B*. *subtilis* natural isolates based on partial sequences of *gyrA*. Natural isolates that were sensitive to LXG toxins produced by strain 3610 are shown in red. Thirteen *B*. *subtilis* strains and *B*. *licheniformis* DSM13 from S2 Fig were used as references (shown in blue).
(PDF)

S1 Table Distribution of LXG toxin-antitoxin systems in *B*. *subtilis*. (XLSX)

S2 Table Primers used in this study. (DOCX)

S1 Text Construction of *B*. *subtilis* strains. (DOCX)

S1 Dataset Numerical data used in this study. (XLSX)
