# Supplementary material for: Diverse LXG toxin and antitoxin systems specifically mediate intraspecies competition in Bacillus subtilis biofilms
Source: PLoS Genet. 2021 Jul 19;17(7):e1009682. doi: 10.1371/journal.pgen.1009682 (PMC8321402; doi:10.1371/journal.pgen.1009682)
Supplement: S1 Fig — (A) LXG toxins of B. subtilis strain 3610. Identical amino acid residues in three or more proteins are highlighted in yellow. (B) LXG toxins of S. intermedius. Identical amino acid residues in two or three proteins are highlighted in green. Sequences were aligned using CLUSTALW (https://www.genome.jp/tools-bin/clustalw). (PDF) [file pgen.1009682.s001.pdf]

A

```
YokI        -MKVFEADSLLSEADKRTKEYKELRSQMVKLKKAFKAVADLDDSKFSGKGADNIKAFYHD
YobL        -MKVFEADSLLFEADKRTKEYKELRSQMVKLKKAFKEVANLDDSEFSGKGADNIKAFYHG
YeeF        -MKVFEAKTLLSEATDRAKEYKELRTQMVNLRKALKGVADLSDSEFSGKGASNIKAFYHD
YqcG        -MKVFEAKTLLTEAEKRAQEYKDLKSKMVKLKKAFKAVADLDDSEFSGKGANNIKSFYED
YwqJ        MSKVFESKSLIEEAKSRKKQYETLEEQLNTLKKAFQGVADLGDN-FKGNGADNIKDFFQG
YxiD        -MKTLDVHALHEGIQHTIEKLDKQKQQLEKLEKSVEHLAGMKDA-LKGKGGDAIRTFYEE

YokI        -HVGVTDQWIDLIDMKIVFLSSISAKLEDAK-MSDAYIEESFLEHELVNAYTKSKSIMSE
YobL        -HVGVTDQWIDLIDMKIAFLSSMSATLEDAK-MSDAYIEESFLEHELANAYAKSKSIMSE
YeeF        -HVGVADQWIDYIDMKIAFFNSIAGAAEDKG-LSDAYIEESFLEHELANANKKSKSIMSE
YqcG        -QAGIADQWIDLIEMKISFLTSIPGFLEDAN-LSDAYIEETFLAHELANAYTKSKSIMSE
YwqJ        -QAEIVDSWLTLVSAQIAFLNGISGDIKDQE-LNDSYVETSFLDHELPNGDLKASEIVSA
YxiD        CHKPFLLFFGIFIDEYKKVLKQTQHAISSVESNSHGMIAEAFLSHDARHGVKHAREVTEQ

YokI        QKKAMKDILNDINDILPLEIFSTEDFKDKLSSADDKREKTIDKINKLDEDLKTEYAETEQ
YobL        QKKAMKDILNNINDILPLEIFSTEDFKDKLSSADDKREKTIDKLNKLDEDLKTEYAETEP
YeeF        QKKAMKDILNDIDDILPLDLFSTETFKDELADANDKRKKTLEKLDALDEDLKTEYALSEP
YqcG        QKKAMKDILNDINDILPLDLFSTETFKNELSSAEKKRKEAIEKMDEVDQNLTSEYGLSEA
YwqJ        HKEEIDSILSGISDIIDLDMYTLDDYADKMGDAQKIRRDTITAVDKLDESLTTEYQNLES
YxiD        LTDAVNRQTSAIDHIVSLPTVNDSFFRMETEQAERLISDTLNKLFQFDGQQTQALEAAKS

YokI        NEQFI
YobL        NEQFI
YeeF        NEQFI
YqcG        NEQMI
YwqJ        LDNAV
YxiD        DFQTM
```

B

```
TelA        --MKIDMTEVNNQKTALANSISNLNGQIDTAKNSLTNLTSSSSLTGDVKTAIDAKINNYQ
TelB        MSFHVEVAELYRSLQTLVNESETAIRRLEETKTSYHRILTSNSMHGQVGTAISEEINTAH
TelC        --MSIKMDLGKSQAQADSVKKMCQAQMAGYQALQQSIQVFANDTESLKGKAYDSAR-AYF

TelA        VPLLTNFTNALTTLSAQYDKTIEQFQSTVSENAADAVIDTDYLQGLLDNYSGIETSISTI
TelB        TVVVVRLKHLLYLLHQDFSKELTSFQEATGESSPTAVLDEEVLLHANQTYQSVSSKVEAI
TelC        STILLPLAQGSELYAESLQKAIAKLPEEYQARVDTKSWDEEDLLRLIRQEEEQIHQLEAI

TelA        NTETSTIYSSISDIISLTNPDSSTITTPLAAAKTILTDTKTNMESFNGWTRGTELADLLL
TelB        HRSVQQAAAKVSDLIVEES-DRHHYHAFQDNLYNAREVITKAIEQVSAWDAAGQTLGSEG
TelC        YESISRLEISRTEKQNLRRTNTDLIRGHQANKR-VYEVILEGLRVYDTYSATLFEELEEI

TelA        SQTQTIETLIGYAS
TelB        ALQELQLLLTSLR-
TelC        DLQLQRGL------
```
