# Supplementary figures and images for: Diverse LXG toxin and antitoxin systems specifically mediate intraspecies competition in Bacillus subtilis biofilms
Source: PLoS Genet. 2021 Jul 19;17(7):e1009682. doi: 10.1371/journal.pgen.1009682 (PMC8321402; doi:10.1371/journal.pgen.1009682)

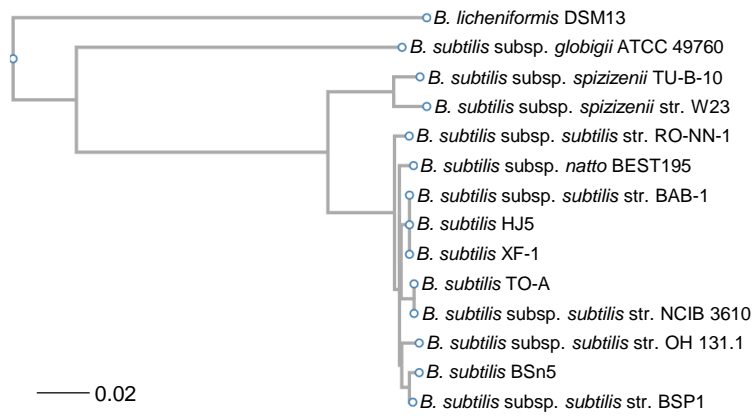

Supplement: S2 Fig — The phylogenetic tree is constructed based on full-length gyrA alignment. B. licheniformis DSM13 was used as an outgroup reference. (PDF) [file pgen.1009682.s002.pdf]

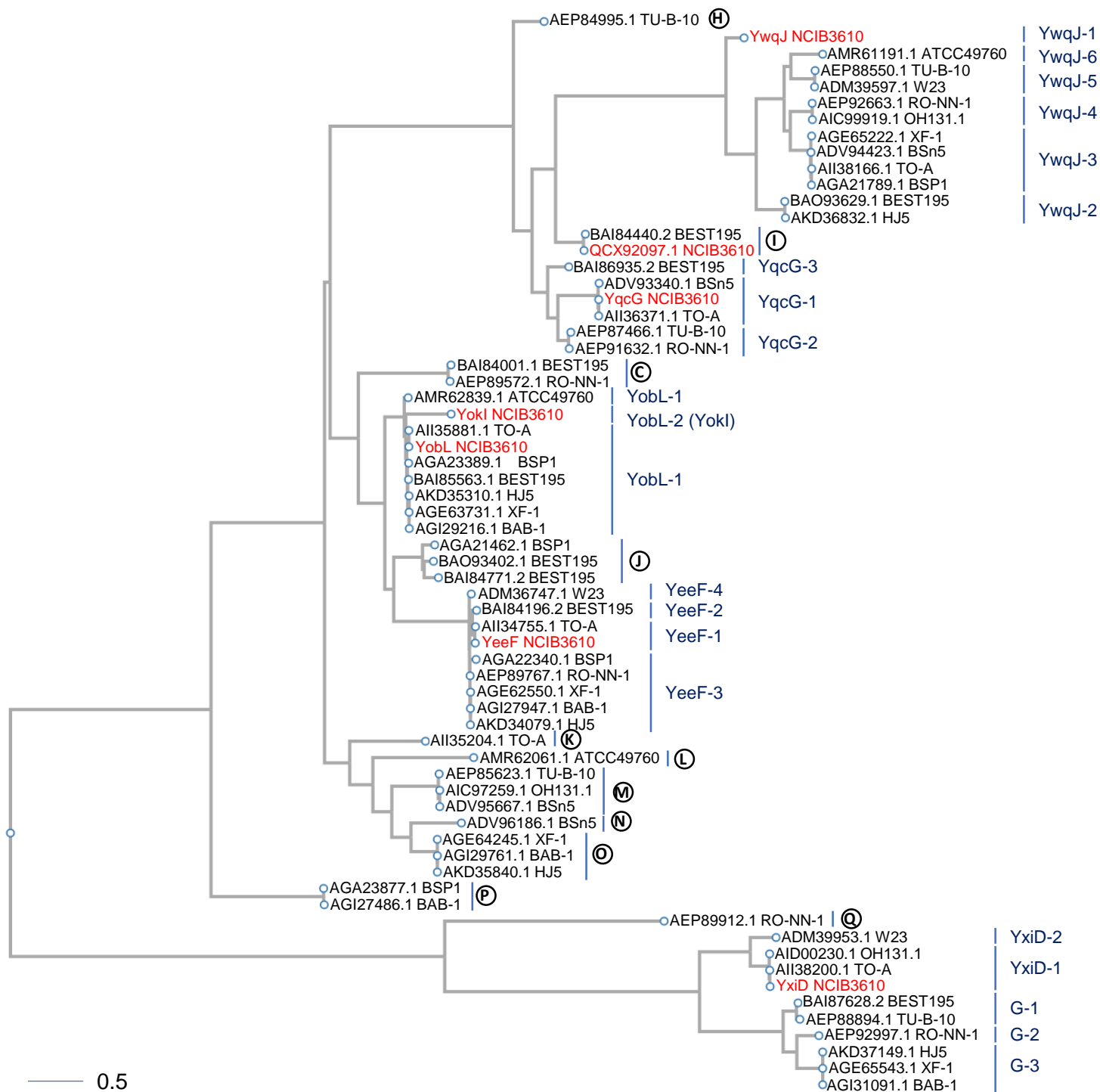

Supplement: S3 Fig — Group and subgroup names are indicated at the right of the clades. The proteins of strain 3610 are shown in red. (PDF) [file pgen.1009682.s003.pdf]

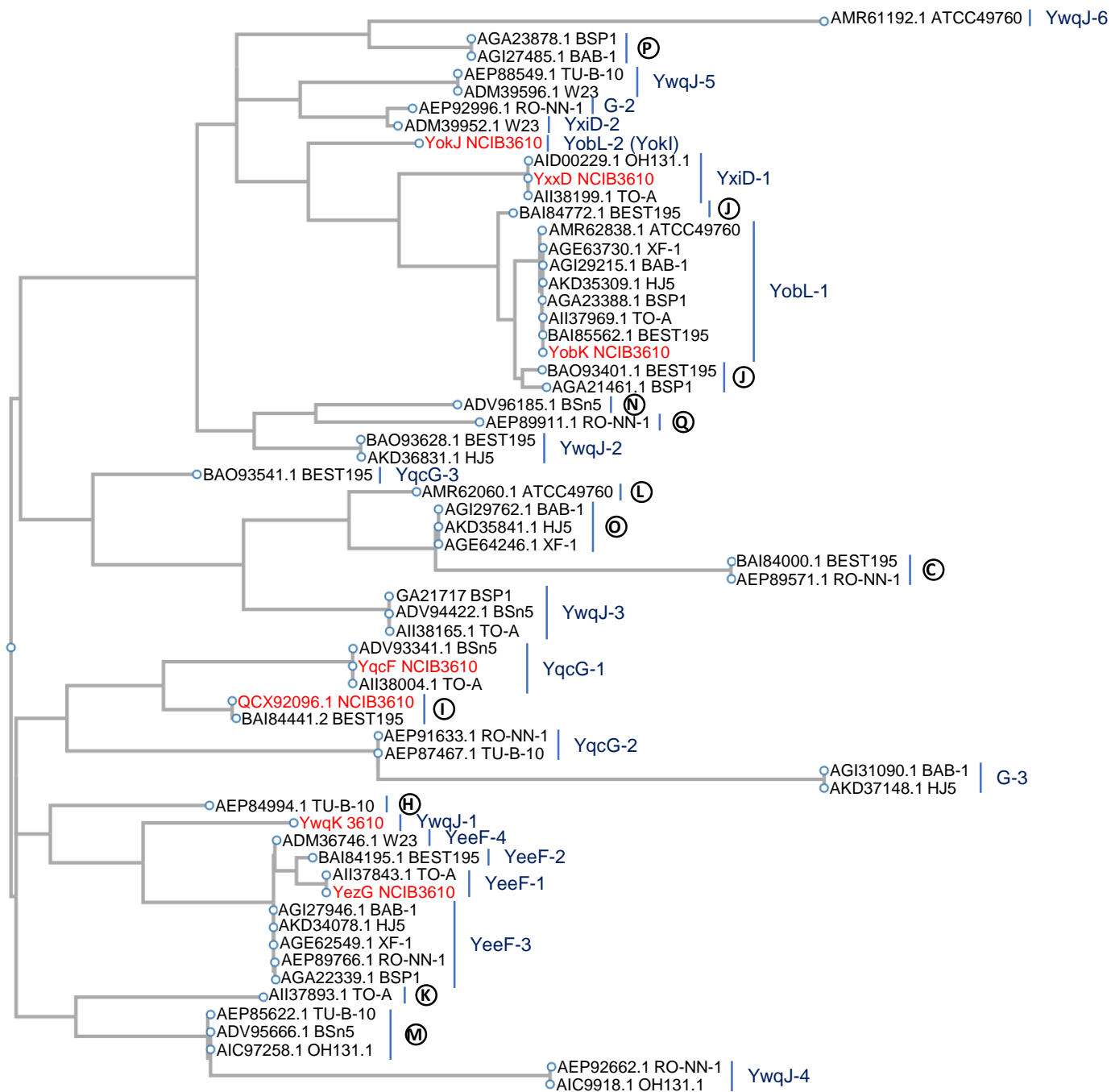

— 0.5

Supplement: S5 Fig — Group and subgroup names are indicated on the right. The proteins of strain 3610 are shown in red. (PDF) [file pgen.1009682.s005.pdf]

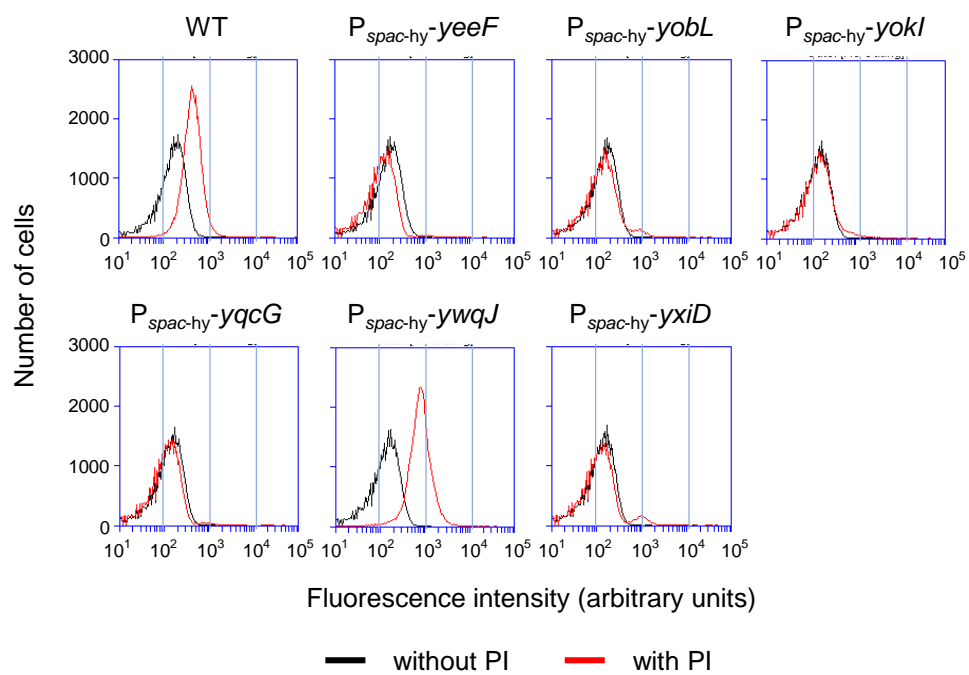

Supplement: S7 Fig — Cells collected at 1 h shown in Fig 3B were fixed in 70% ethanol. These cells were stained with the DNA-dye propidium iodide (PI), and cellular DNA content was analyzed by flow cytometry. Plots of unstained cells are shown as references. (PDF) [file pgen.1009682.s007.pdf]

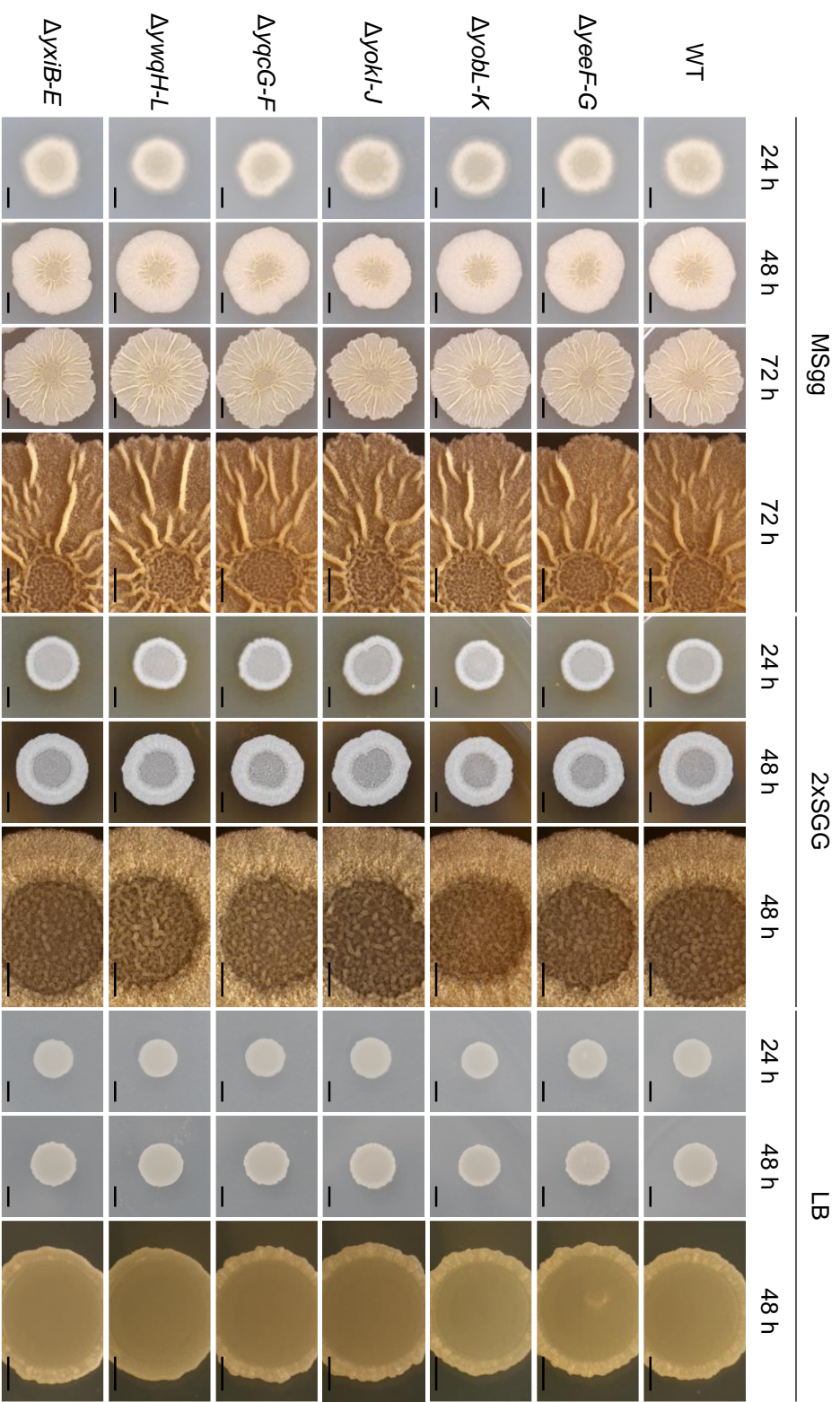

Supplement: S8 Fig — Wild-type and mutant strains were grown on three solid media. Top–down views of colonies are shown. Magnified images of colonies were taken with a stereomicroscope. Scale bar, 2 mm. (PDF) [file pgen.1009682.s008.pdf]

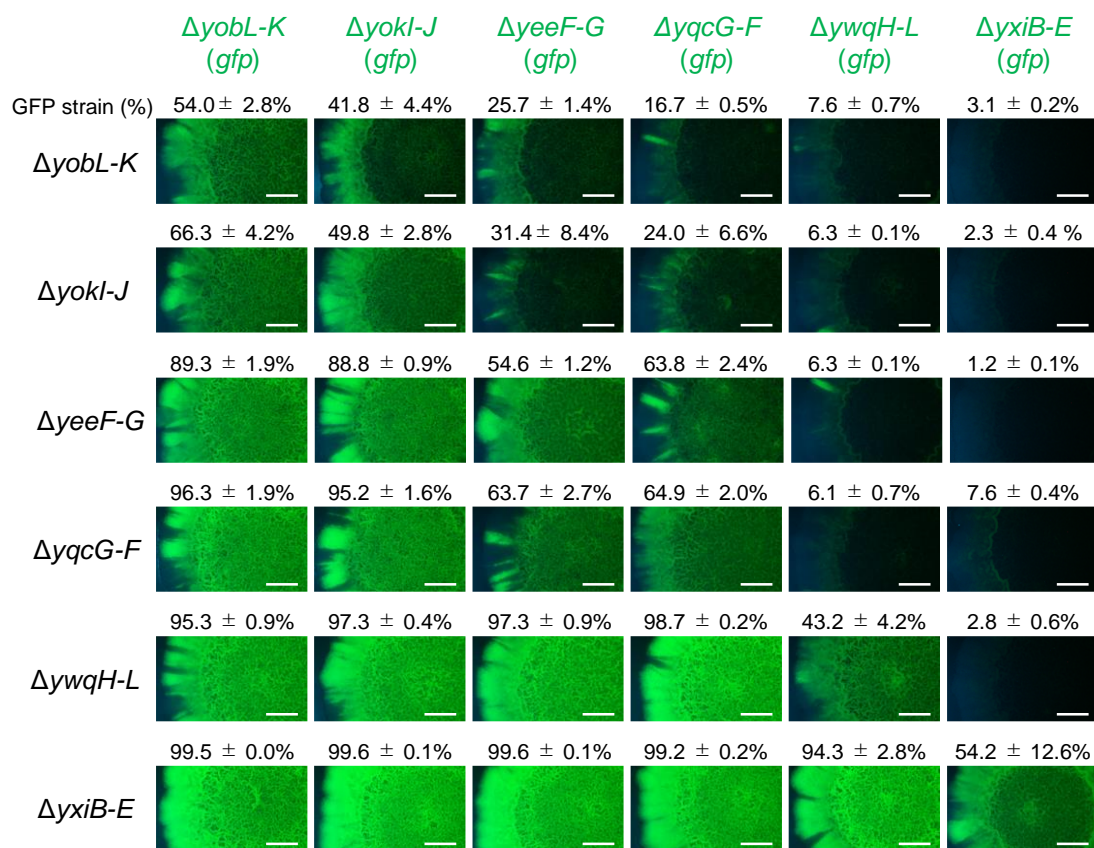

Supplement: S9 Fig — Indicated LXG toxin–antitoxin deletion mutants, with or without the gfp reporter, were co-cultured at a 1:1 ratio on 2×SGG solid medium as described in Fig 5. The proportion of GFP-reporter strains within colonies is indicated above fluorescent images of colonies. The experiments were repeated at least three times, and representative examples are shown in the figures. Percentages are presented as mean ± standard deviation (n = 3). Scale bar, 2 mm. (PDF) [file pgen.1009682.s009.pdf]

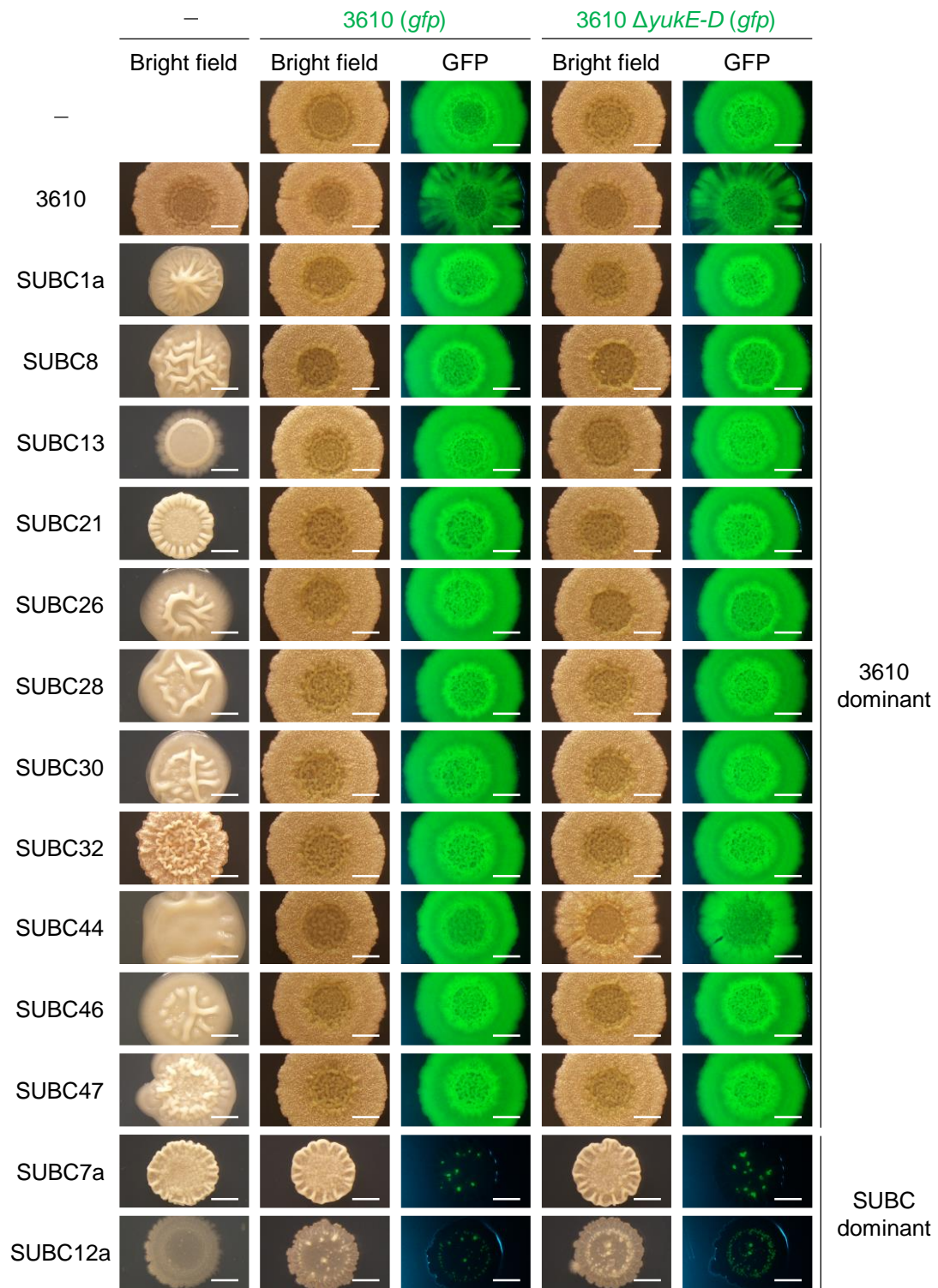

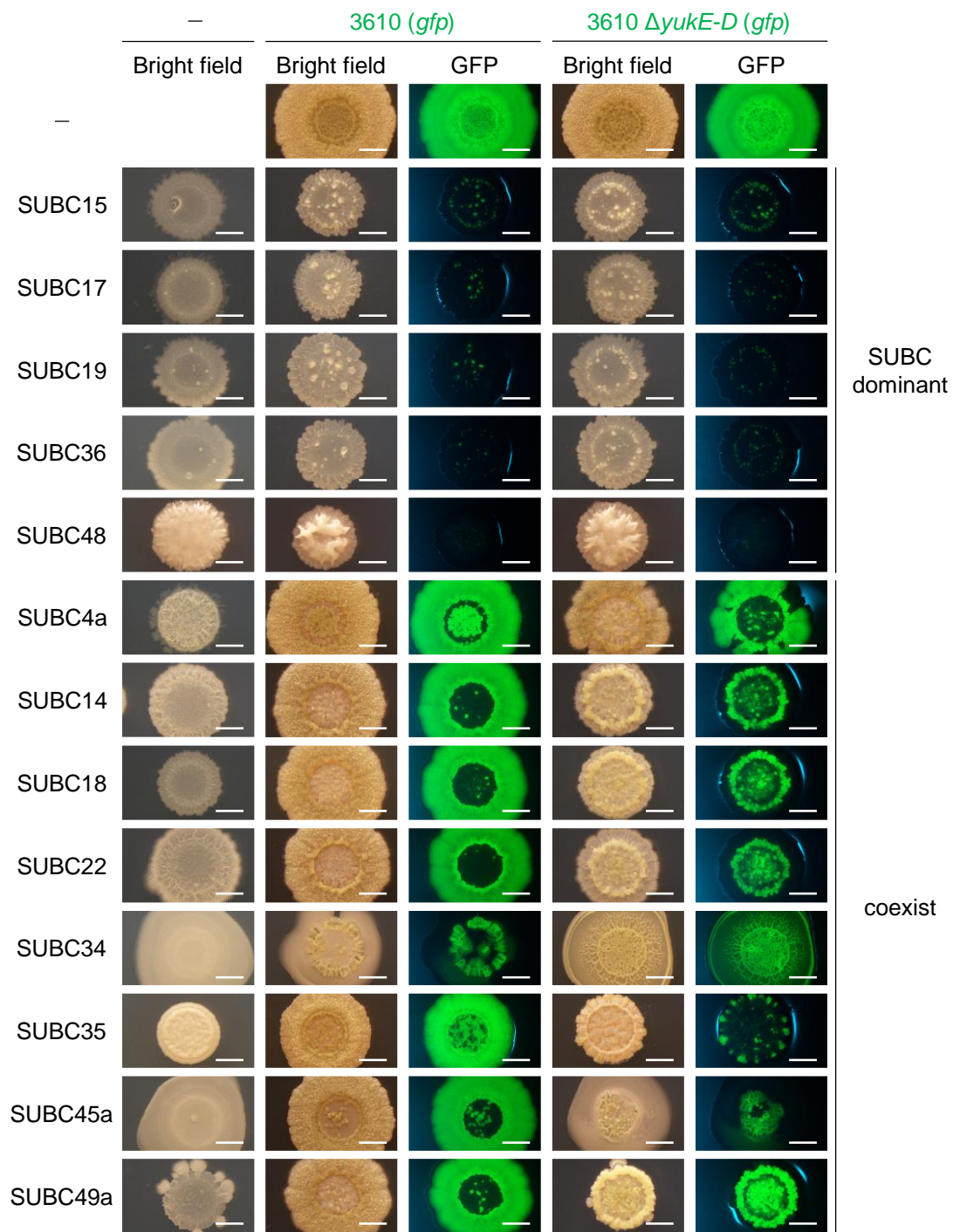

Supplement: S10 Fig — The GFP-labeled strain 3610 or the ΔyukE–D mutant was co-cultured with one of 26 B. subtilis natural isolates at a ratio of 1:1 on MSgg medium. After 48 h of cultivation, the morphology and GFP fluorescence of colonies were observed with a stereomicroscope. Single-strain cultures are shown as references. The classification of co-cultured colonies is indicated on the right. The experiments were repeated twice and confirmed the reproducibility. Scale bar, 2 mm. (PDF) [file pgen.1009682.s010.pdf]

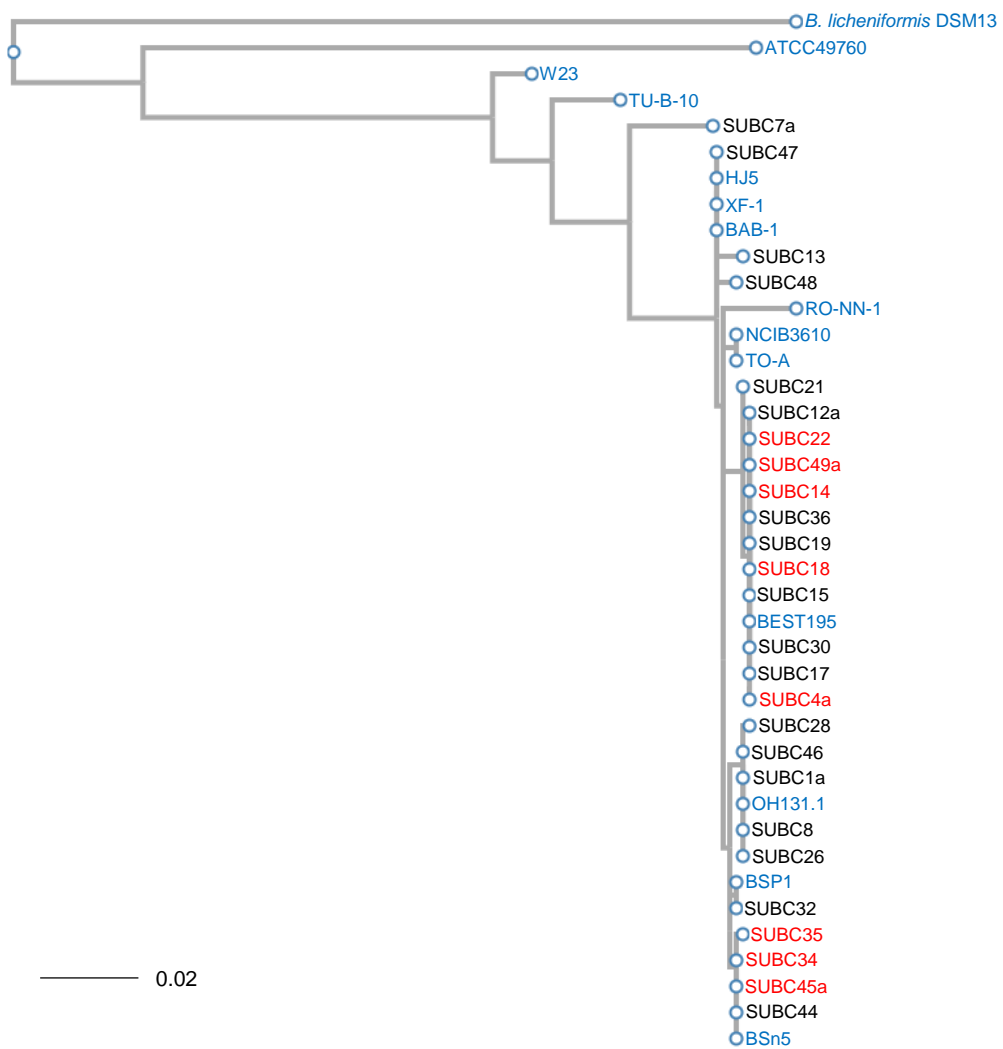

Supplement: S11 Fig — Natural isolates that were sensitive to LXG toxins produced by strain 3610 are shown in red. Thirteen B. subtilis strains and B. licheniformis DSM13 from S2 Fig were used as references (shown in blue). (PDF) [file pgen.1009682.s011.pdf]
