# Supplementary material for: Diverse LXG toxin and antitoxin systems specifically mediate intraspecies competition in Bacillus subtilis biofilms
Source: PLoS Genet. 2021 Jul 19;17(7):e1009682. doi: 10.1371/journal.pgen.1009682 (PMC8321402; doi:10.1371/journal.pgen.1009682)
Supplement: S4 Fig — Indicated proteins were aligned using the Clustal Omega website (https://www.ebi.ac.uk/Tools/msa/clustalo/). Identical (*) and conserved (: or.) amino acids are indicated below the alignment according to the Clustal Omega scheme. Each subgroup of proteins is shown in a different color. Alignments of YeeF group proteins (A), YobL and C group proteins (B), YqcG group proteins (C), YwqH group proteins (D), and YxiD and G group proteins (E). Positions of LXG and toxin domains in proteins from strain 3610 (A–D) or proteins from strain W23 (E) are indicated above the sequences. (PDF) [file pgen.1009682.s004.pdf]

A

```
                                                 LXG
YeeF_NCIB3610           --MKVFEAKTLLSEATDRAKEYKELRTQMVNLRKALKGVADLSDSEFSGKGASNIKAFYH      58
AII34755.1_TO-A         --MKVFEAKTLLSEATDRAKEYKELRTQMVNLRKALKSVADLSDSEFSGKGASNIKAFYH      58
BAI84196.2_BEST195      --MKVFEAKTLLSEAADRAKEYKELRTQMVNLRKALKSVADLSDSEFSGKGASNIKAFYH      58
AGA22340.1_BSP1         -------------------------------MRKALKSVADLSDSEFSGKGASNIKAFYH      29
AEP89767.1_RO-NN-1      --MKVFEAKTLLSEATDRAKEYKELRTQMVNLRKALKSVADLSDSEFSGKGASNIKAFYH      58
AGE62550.1_XF-1         MVMKVFEAKTLLSEATDRAKEYKELRTQMVNLRKALKSVADLSDSEFSGKGASNIKTFYH      60
AKD34079.1_HJ5          --MKVFEAKTLLSEATDRAKEYKELRTQMVNLRKALKSVADLSDSEFSGKGASNIKTFYH      58
AGI27947.1_BAB-1        --MKVFEAKTLLSEATDRAKEYKELRTQMVNLRKALKSVADLSDSEFSGKGASNIKTFYH      58
ADM36747.1_W23          --MKVLEVKTLLSEATDRAKEYKELRTQMVNLRKALKSVADLGDSEFSGKGASNIKAFYH      58
                                                       :*****.****.*************:***
                                                LXG
YeeF_NCIB3610           DHVGVADQWIDYIDMKIAFFNSIAGAAEDKGLSDAYIEESFLEHELANANKKSKSIMSEQ      118
AII34755.1_TO-A         DHVGVTDQWIDYIDMKIAFFNSIAGAAEDKGLSDAYIEESFLEHELENANKKSKSIMSEQ      118
BAI84196.2_BEST195      DHVGVTDQWIDYIDMKIAFFNSIAGAAEDKGLSDAYIEESFLEHELANANKKSKSIMSEQ      118
AGA22340.1_BSP1         DHVGVTDQWIDYIDMKSHFSTASPEPPKTKASQTHTSKNPSWSTNWQMPIKKSKSIMSEQ      89
AEP89767.1_RO-NN-1      DHVGVTDQWIDYIDMKIAFFNSIAGAAEDKGLSDAYIEESFLEHELANANKKSKSIMSEQ      118
AGE62550.1_XF-1         DHVGVTDQWIDYIDMKIAFFNSIAGAAEDKGLSDAYIEESFLEHELANANKKSKSIMSEQ      120
AKD34079.1_HJ5          DHVGVTDQWIDYIDMKIAFFNSIAGAAEDKGLSDAYIEESFLEHELANANKKSKSIMSEQ      118
AGI27947.1_BAB-1        DHVGVTDQWIDYIDMKIAFFNSIAGAAEDKGLSDAYIEESFLEHELANANKKSKSIMSEQ      118
ADM36747.1_W23          DHVGVTDQWIDYIDMKIAFFNSIAGAAEDKGLSDAYIEESFLEHELANAHKKSKSIMSEQ      118
                        *****:**********  * .:     : *. .    ::   . :     **********
                                                LXG
YeeF_NCIB3610           KKAMKDILNDIDDILPLDLFSTETFKDELADANDKRKKTLEKLDALDEDLKTEYALSEPN      178
AII34755.1_TO-A         KKAMKDILNDIDDILPLDLFSTETFKDELADANDKRKKTLEKLDALDEDLKTEYALSEPN      178
BAI84196.2_BEST195      KKAMKDILNDIDDILPLDLFSTETFKDELADANDKRKKTLDKLDALDEDLKTEYALSEPN      178
AGA22340.1_BSP1         KKAMKDILNDVDDILPLDLFSTETFKDELADANDKRKKTLEKLDALDEDLKTEYALSEPN      149
AEP89767.1_RO-NN-1      KKAMKDILNDIDDILPLDLFSTETFKDELADANNKRKKTLEKLDALDEDLKTEYALSEPN      178
AGE62550.1_XF-1         KKAMKDILNDIDDILPLDLFSTETFKDELADANDKRKKTLEKLDALDEDLKTEYALSEPN      180
AKD34079.1_HJ5          KKAMKDILNDIDDILPLDLFSTETFKDELADANDKRKKTLEKLDALDEDLKTEYALSEPN      178
AGI27947.1_BAB-1        KKAMKDILNDIDDILPLDLFSTETFKDELADANDKRKKTLEKLDALDEDLKTEYALSEPN      178
ADM36747.1_W23          KKAMKDILNDIDNILPLDLFSTETFNDELADANDKRKKTLEKLDALDEDLKTEYALSEPN      178
                        **********:*:************:*******:******:*******************
                                  LXG
YeeF_NCIB3610           EQFIKSDFQKLQEATGKGKNATPIHYNAKAYRESDIHKKKGDIEKRTEAYLKIKKEEAKE      238
AII34755.1_TO-A         EQFIKSDFQKLQEATGKGKNAIPIHYNAKAYRESDIHKKKGDIEKRTDAYLKIKKEEAKE      238
BAI84196.2_BEST195      EQFIKSDFQKLQEATGKGKNATPIHYNAKAYRESDIHKKKGDIEKRTEAYLKIKTEEAKK      238
AGA22340.1_BSP1         EQFIKSDFQKLQEATGKGKNATPIHYNAKAYRESDIHKKKGDIEKRTEAYLKIKKEEAKE      209
AEP89767.1_RO-NN-1      EQFIKSDFQKLQEATGKGKNATPIHYNAKAYRESDIHKKKGDIEKRTEAYLKIKKEEAKE      238
AGE62550.1_XF-1         EQFIKSDFQKLQEATGKGKTATPIHYNAKAYRESDIHKKKGDIEKRTEAYLKIKKEEAKE      240
AKD34079.1_HJ5          EQFIKSDFQKLQEATGKGKNATPIHYNAKAYRESDIHKKKGDIEKRTEAYLKIKKEEAKE      238
AGI27947.1_BAB-1        EQFIKSDFQKLQEATGKGKNATPIHYNAKAYRESDIHKKKGDIEKRTEAYLKIKKEEAKE      238
ADM36747.1_W23          EQFIKSDFQKLQEATGKGKNATPIHYNAKAYRESDIHKKKGDIEKRTEAYLKIKKEEAKE      238
                        *******************.* *************************:******.****:

YeeF_NCIB3610           REIEKLKERLKNYDYADADEFYEMAKTIGYENLTAEQQRYFTQIENTRELEAGFKGVAVG      298
AII34755.1_TO-A         REIEKLKERLKNYDYADADEFYEMAKTIGYENLTAEQQRYFTQIENTRELEAGFKGVAVG      298
BAI84196.2_BEST195      REIEKLKERLKNYDYADADEFYSMAKTIGYENLTAEQQRYFTQIENTRELEAGFKGVAVG      298
AGA22340.1_BSP1         REIEKLKERLKNYDYADADEFYEMAKTIGYKNLTAEQQRYFTQIENTRELEAGFKGVAVG      269
AEP89767.1_RO-NN-1      REIEKLKERLKNYDYADADEFYEMAKTIGYENLTAEQQRYFTQIENTRELEAGFKGVAVG      298
AGE62550.1_XF-1         REIEKLKERLKNYDYADADEFYEMAKTIGYENLTAEQQRYFTQIENTRELEAGFKGVAVG      300
AKD34079.1_HJ5          REIEKLKERLKNYDYADADEFYEMAKTIGYENLTAEQQRYFTQIENTRELEAGFKGVAVG      298
AGI27947.1_BAB-1        REIEKLKERLKNYDYADADEFYEMAKTIGYENLTAEQQRYFTQIENTRELEAGFKGVAVG      298
ADM36747.1_W23          REIEKLKERLKNYDYADADEFYEMAKTIGYENLTAEQQRYFTQIENTRELEAGFKGVAVG      298
                        **********************.*******:*****************************

YeeF_NCIB3610           LYDSGKDAVVGLWDMVTDPGGTVEAITGAMAHPIKTYEAISAAIEESYQKDMVNGDTYSR      358
AII34755.1_TO-A         LYDSGKDAVVGLWDMVTDPGGTVEAITGAMAHPIKTYEAISAAIEESYQKDMVNGDTYSR      358
BAI84196.2_BEST195      LYDSGKDAVVGLWDMVTDPGGTVEAITGAMAHPIKTYEAISAAIEESYQKDMVNGDTYSR      358
AGA22340.1_BSP1         LYDSGKDAVVGLWDMVTDPGGTVEAITGAVAHPIKTYEAISAAIEESYQKDMVNGNTYSR      329
AEP89767.1_RO-NN-1      LYDSGKDAVVGLWDMVTDPGGTVEAITGAVAHPIKTYEAISAAIEESYQKDMVNGDTYSR      358
AGE62550.1_XF-1         LYDSGKDAVVGLWDMVTDPGGTVEAITGAVAHPIKTYEAISAAIEESYQKDMVNGDTYSR      360
AKD34079.1_HJ5          LYDSGKDAVVGLWDMVTDPGGTVEAITGAVAHPIKTYEAISAAIEESYQKDMVNGDTYSR      358
AGI27947.1_BAB-1        LYDSGKDAVVGLWDMVTDPGGTVEAITGAVAHPIKTYEAISAAIEESYQKDMVNGDTYSR      358
ADM36747.1_W23          LYDSGKDAVVGLWDMVTDPGGTVEAITGAVAHPIKTYEAISAAIEESYQKDMVNGDTYSR      358
                        *****************************:*************************:****

YeeF_NCIB3610           ARWVSYAVGTVVTSIVGTKGVGAVSKTGTAAKVTTKVKTAASKSATAQKAITVSKQTVDH      418
AII34755.1_TO-A         ARWVSYAVGTVVTSIVGTKGVGAVSKTGTAAKVTTKVKTAASKSATAQKAITVSKQTVDH      418
BAI84196.2_BEST195      ARWVSYAVGTVVTSIVGTKGVGAVSKTGTAAKVTAKVKTAANKSATAQKAIMVSKQTVDH      418
AGA22340.1_BSP1         ARWVSYAVGTVVTSIVGTKGVGAVSKTGTAAKVTAKVKTTASKSATAQKAITVSKQTVDH      389
AEP89767.1_RO-NN-1      ARWVSYAVGTVVTSIVGTKGVGAVSKTGTAAKVTAKVKTTASKSATAQKAITVSKQTVDH      418
AGE62550.1_XF-1         ARWVSYAVGTVVTSIVGTKGVGAVSKTGTAAKMTAKVKTTASKSATAQKAITVSKQTVDH      420
AKD34079.1_HJ5          ARWVSYAVGTVVTSIVGTKGVGAVSKTGTAAKMTAKVKTTASKSATAQKAITVSKQTVDH      418
AGI27947.1_BAB-1        ARWVSYAVGTVVTSIVGTKGVGAVSKTGTAAKMTAKVKTTASKSATAQKAITVSKQTVDH      418
ADM36747.1_W23          ARWVSYAVGTVVTSIVGTKGVGAVSKTGTATKVTTKVKTAASKSATAQKAITVSKQTIDH      418
                        ******************************:*:*:****:*.********* *****:**
```

S4 Fig A. continued

A

```
YeeF_NCIB3610          IKQKVNTGIEVSKKHVKTKLNQIGDLTLADILPYHPRHDLVPAGVPYNAVNGVTLKEGLQ    478
AII34755.1_TO-A        IKQKVNTGIEVSKKHVKTKLNQIGDLTLADILPYHPRHDLVPAGVPYNAVNGVTLKEGLQ    478
BAI84196.2_BEST195     IKQKVNTGIEVSKKHVKTRLNQIGDLTLADILPYHPRHDLVPAGVPYNAVNGVTLKEGLQ    478
AGA22340.1_BSP1        IKQKVNTGIEVSKKHVKTKLNQIGDLTLADILPYHPRHDLVPAGVPYNAVNGVTLKEGLQ    449
AEP89767.1_RO-NN-1     IKQKVNTGIEVSKKHVKTKLNQIGDLTLADILPYHPRHDLVPAGVPYNAVNGVTLKEGLQ    478
AGE62550.1_XF-1        IKQKVNTGIEGSKKHVKTKLNQIGDLTLADILPYHPRHDLVPAGVPYNAVNGVTLKEGLQ    480
AKD34079.1_HJ5         IKQKVNTGIEGSKKHVKTKLNQIGDLTLADILPYHPRHDLVPAGVPYNAVNGVTLKEGLQ    478
AGI27947.1_BAB-1       IKQKVNTGIEGSKKHVKTKLNQIGDLTLADILPYHPRHDLVPAGVPYNAVNGVTLKEGLQ    478
ADM36747.1_W23         IKQKVNKGIEVSKKHVKTKLNQIGDLTLADILPYHPRHDLVPAGVPYNAVNGVTLKEGLQ    478
                       ******.*** *******:*****************************************
                                                                     toxin
YeeF_NCIB3610          KFAKVILPKPYGTSSSGRRTPAPHVPPVTVKYGEHFARWSRKKVLKPNIIYKTKEGYTYT    538
AII34755.1_TO-A        KFAKVILPKPYGTSSSGRRTPAPHVPPVTVKYGEHFARWSRKKVLKPNVIYKTKEGYTYT    538
BAI84196.2_BEST195     KFAKVILPKPYGTSSSGRRTPAPHVPPVTVKYGEHFARWSRKKVLKPNVIYKTKEGYTYT    538
AGA22340.1_BSP1        KFAKVILPKPYGTSSSGRRTPAPHVPPVTVKYGEHFARWSRKKVLKPNIIYKTKEGYTYT    509
AEP89767.1_RO-NN-1     KFAKVILPKPYGTSSSGRRTPAPHVPPVTVKYGEHFARWSRKKVLKPNIIYKTKEGYTYT    538
AGE62550.1_XF-1        KFAKVILPKPYGTSSSGRRTPAPHVPPVTVKYGEHFARWSRKKVLKPNIIYKTKEGYTYT    540
AKD34079.1_HJ5         KFAKVILPKPYGTSSSGRRTPAPHVPPVTVKYGEHFARWSRKKVLKPNIIYKTKEGYTYT    538
AGI27947.1_BAB-1       KFAKVILPKPYGTSSSGRRTPAPHVPPVTVKYGEHFARWSRKKVLKPNIIYKTKEGYTYT    538
ADM36747.1_W23         KFAKVILPKPYGTSSSGRRTPAPVVPPVTVKYGEHYARWSRKKVLKPNIIYKTKEGYTYT    538
                       ********************** ***********:************:************
                                                toxin
YeeF_NCIB3610          TDNYGRITSVKADLQLGEAKRNQYAQTNAGKPQDRKPDDDGGHLIATQFKGSGQFDNIVP    598
AII34755.1_TO-A        TDNYGRITSVKADLQLGEAKRNQYAQTNAGKPQDRKPDDDGGHLIATQFKGSGQFDNIVP    598
BAI84196.2_BEST195     TDNYGRITSVKADLQLGEAKRNQYAQTNAGKPQDRKPDDDGGHLIATQFKGSGQFDNIVP    598
AGA22340.1_BSP1        TDNYGRITSVKADLQLGEAKRNQYAQTNAGKPQDRKPDDDGGHLIATQFKGSGQFDNIVP    569
AEP89767.1_RO-NN-1     TDNYGRITSVKADLQLGEAKRNQYAQTNAGKPQDRKPDDDGGHLIATQFKGSGQFDNIVP    598
AGE62550.1_XF-1        TDNYGRITSVKADLQLGEAKRNQYAQTNAGKPQDRKPDDDGGHLIATQFKGSGQFDNIVP    600
AKD34079.1_HJ5         TDNYGRITSVKADLQLGEAKRNQYAQTNAGKPQDRKPDDDGGHLIATQFKGSGQFDNIVP    598
AGI27947.1_BAB-1       TDNYGRITSVKADLQLGEAKRNQYAQTNAGKPQDRKPDDDGGHLIATQFKGSGQFDNIVP    598
ADM36747.1_W23         TDNYGRITSVKADLQLGEAKRNQYAQSHAGKPQDRKPDDDGGHLIATQFKGSGQFDNIVP    598
                       **************************::********************************
                                           toxin
YeeF_NCIB3610          MNSQINRSGGKWYEMEQEWAKALSKKPPKKVAVQIEPVYSGDSLRPSYFDVTYKIGSRKE    658
AII34755.1_TO-A        MNSQINRSGGKWYEMEQEWAKALSKKPPKKVAVQIEPVYSGDSLRPSYFDVTYKIGSRKE    658
BAI84196.2_BEST195     MNSQINRSGGKWYNMEQVWSKALSQKPPKQVTVKINNIYEGDSLRPSKFIVVYKVGDKEP    658
AGA22340.1_BSP1        MNSQINRSGGRWYEMEQEWAKALKEEPPQKVNVNIKAIYKGDSLRPDKFIVKYRIGDADF    629
AEP89767.1_RO-NN-1     MNSQINRSGGRWYEMEQEWAKALKEEPPQKVNVNIKAIYKGDSLRPDKFIVKYRIGDADF    658
AGE62550.1_XF-1        MNSQINRSGGRWYEMEQEWAKALKEEPPQKVNVNIKAIYKGDSLRPDKFIVKYRIGDADF    660
AKD34079.1_HJ5         MNSQINRSGGRWYEMEQEWAKALKEEPPQKVNVNIKAIYKGDSLRPDKFIVKYRIGDADF    658
AGI27947.1_BAB-1       MNSQINRSGGRWYEMEQEWAKALKEEPPQKVNVNIKAIYKGDSLRPDKFIVKYRIGDADF    658
ADM36747.1_W23         MNSQINRSGGRWYEMEQEWAKALKEEPPQKVNVNIKAIYKGDSLRPDKFIVKFRIGDADF    658
                       **********:**:*** *:***.::**::* *:*: :*.******. * * :::*. .

YeeF_NCIB3610          ISVSIKNQPGG          669
AII34755.1_TO-A        ISVSIKNQPGG          669
BAI84196.2_BEST195     VRTIIKNKAGG          669
AGA22340.1_BSP1        EKVTLKNQSGG          640
AEP89767.1_RO-NN-1     EKVTLKNQSGG          669
AGE62550.1_XF-1        EKVTLKNQSGG          671
AKD34079.1_HJ5         EKVTLKNQSGG          669
AGI27947.1_BAB-1       EKVTLKNQSGG          669
ADM36747.1_W23         EKVTIKNQSGG          669
                         . :**: **
```

S4 Fig A

B

```
                                                       LXG
AMR62839.1_ATCC49760     MKVFEADSLLSEADKRTKEYKELRSQMVKLRKAFKAVADLDESEFSGKGANNIKAFYHDH     60
YobL_NCIB3610            MKVFEADSLLFEADKRTKEYKELRSQMVKLKKAFKEVANLDDSEFSGKGADNIKAFYHGH     60
AII35881.1_TO-A          MKVFEADSLLFEADKRTKEYKELRSQMVKLKKAFKEVANLDDSEFSGKGADNIKAFYHGH     60
AGA23389.1_BSP1          MKVFEADSLLFEADKRTKEYKELRSQMVKLRKAFQNVANLDDSEFSGKGADNIKAFYHDH     60
BAI85563.1_BEST195       MKVFEADSLLFEADKRTKEYKELRSQMVKLRKAFQNVANLDDSEFSGKGADNIKAFYHDH     60
AGI29216.1_BAB-1         MKVFEADSLLFEADKRTKEYKELRSQMVKLRKAFQNVATLDDSEFSGKGADNIKAFYHDH     60
AKD35310.1_HJ5           MKVFEADSLLFEADKRTKEYKELRSQMVKLRKAFQNVATLDDSEFSGKGADNIKAFYHDH     60
AGE63731.1_XF-1          --------------------------MVKLRKAFQNVATLDDSEFSGKGADNIKAFYHDH     34
YokI_NCIB3610            MKVFEADSLLSEADKRTKEYKELRSQMVKLKKAFKAVADLDDSKFSGKGADNIKAFYHDH     60
AEP89572.1_RO-NN-1       MKVFEAKSLLSEAENRAKDYKDLKNQMIKLRKAFKAVADLDDSEFSGKGANNIKAFYHDH     60
BAI84001.1_BEST195       MKVFEAKSLLSEAENRAKDYKELKNQMIKLRKAFKAVADLDDSEFSGKGANNIKAFYHDH     60
                                                   *:**:***: ** **:*:******:*******.*

                                                      LXG
AMR62839.1_ATCC49760     VGVTDQWIDLIDMKIAFLSSVSTKVEDAKMSDAYIEESFLEHELVNAYNKSKSIMSEQKK     120
YobL_NCIB3610            VGVTDQWIDLIDMKIAFLSSMSATLEDAKMSDAYIEESFLEHELANAYAKSKSIMSEQKK     120
AII35881.1_TO-A          VGVTDQWIDLIDMKIAFLSSMSATLEDAKMSDAYIEESFLEHELANAYAKSKSIMSEQKK     120
AGA23389.1_BSP1          VGVTDQWIDLIDMKIAFLSSISAKLEDAKMSDAYIEESFLEHELANAYTKSKSIMSEQKK     120
BAI85563.1_BEST195       VGVTDQWIDLIDMKIAFLSSMSAKLEDAKMSDAYIEESFLEHELANAYTKSKSIMSEQKK     120
AGI29216.1_BAB-1         VGVTDQWIDLIDMKIAFLSRMSAKLEDAKMSDAYIEESFLEHELANAYTKSKSIMSEQKK     120
AKD35310.1_HJ5           VGVTDQWIDLIDMKIAFLSRMSAKLEDAKMSDAYIEESFLEHELANAYTKSKSIMSEQKK     120
AGE63731.1_XF-1          VGVTDQWIDLIDMKIAFLSRMSAKLEDAKMSDAYIEESFLEHELANAYTKSKSIMSEQKK     94
YokI_NCIB3610            VGVTDQWIDLIDMKIVFLSSISAKLEDAKMSDAYIEESFLEHELVNAYTKSKSIMSEQKK     120
AEP89572.1_RO-NN-1       VGVTDQWIDLIEMKIAFLTSISGVLEEASLSDAYIEESFLEHELANAYKKSKSIMSEQKK     120
BAI84001.1_BEST195       VGVTDQWIDLIEMKIAFLTSISGVLEDASLSDAYIEESFLEHELTNAYKKSKSIMSEQKK     120
                         ***********:***.**: :*  :*:*.:**************.***  **********

                                                      LXG
AMR62839.1_ATCC49760     AMKDILNDINDILPLEIFSTEDFKDKLSSAEDKREKTVDTLSNLDESLKTEYAETEPNEQ     180
YobL_NCIB3610            AMKDILNNINDILPLEIFSTEDFKDKLSSADDKREKTIDKLNKLDEDLKTEYAETEPNEQ     180
AII35881.1_TO-A          AMKDILNNINDILPLEIFSTEDFKDKLSSADDKREKTIDKLNKLDEDLKTEYAETEPNEQ     180
AGA23389.1_BSP1          AMKDILNDINDILPLEIFSTENFKDKLSSADDKREKTIDKLNKLDENLKSEYAETEPNEQ     180
BAI85563.1_BEST195       AMKDILNDINDILPLEIFSTEDFKDKLSSADDKREKTIDKLNKLDEDLKTEYAETEPNEQ     180
AGI29216.1_BAB-1         AMKDILNDINDILPLEIFSTEDFKDKLSSADDKREKTIDKLNKLDEDLKTEYVETEPNEQ     180
AKD35310.1_HJ5           AMKDILNDINDILPLEIFSTEDFKDKLSSADDKREKTIDKLNKLDEDLKTEYVETEPNEQ     180
AGE63731.1_XF-1          AMKDILNDINDILPLEIFSTEDFKDKLSSADDKREKTIDKLNKLDEDLKTEYVETEPNEQ     154
YokI_NCIB3610            AMKDILNDINDILPLEIFSTEDFKDKLSSADDKREKTIDKINKLDEDLKTEYAETEQNEQ     180
AEP89572.1_RO-NN-1       AMKDILNDIDDILPLDLFSTETFKDELSSAENKRKKTVDKICDVDENLKTEYAITEPNEQ     180
BAI84001.1_BEST195       AMKDILNDIDDILTLDLFSTETFKDELSSAENKRKKTVDKIGDVDENLKTEYAITEPNEQ     180
                         *******:*:*** *::**** ***:****::**:**:*.: .:**.**:**. ** ***

                                     LXG
AMR62839.1_ATCC49760     FIQQDFKKLQGSTGKGKNATPIHYNAKAYRESDIHKKKDDIEKHSEAYLSVKKEEAKERE     240
YobL_NCIB3610            FIQQDFKKLQESTGKGKNATPIHYNAKAYRESDIHKKKGDIEKHSEAYLSVKKEEAKERE     240
AII35881.1_TO-A          FIQQDFKKLQESTGKGKNATPIHYNAKAYRESDIHKKKGDIEKHSEAYLSVKKEEAKERE     240
AGA23389.1_BSP1          FIQQDFKKLQESTGKGKNATPIHYNAKAYRESDIHKKKGDIEKHSEAYLSVKQEEAKERE     240
BAI85563.1_BEST195       FIQQDFKKLQESTGKGKNATPIHYNAKAYRESDIHKKKGDIEKHSEAYLSVKKEEAKDRE     240
AGI29216.1_BAB-1         FIQQDFKKLQESTGKGKNATPIHYNAKAYRESDIHKKKGDIEKHSESYLSVKKEEAKERE     240
AKD35310.1_HJ5           FIQQDFKKLQESTGKGKNATPIHYNAKAYRESDIHKKKGDIEKHSESYLSVKKEEAKERE     240
AGE63731.1_XF-1          FIQQDFKKLQESTGKGKNATPIHYNAKAYRESDIHKKKGDIEKHSESYLSVKKEEAKERE     214
YokI_NCIB3610            FIQQDFKKLQESTGKGKNATPIHYSAKAYRESDIHKKKGDIEQHSEAYLTVKKEEAKERE     240
AEP89572.1_RO-NN-1       FIKADFQKLQESTGKGKNATPLHYNAKAYRESDIHKKKGDIEKQSEAYLKIKKEEAKKHE     240
BAI84001.1_BEST195       FIKADFQKLQESTGKGKNATPLHYNAKAYRESDIHKKKGDIEKQSEAYLKIKKEEANKCE     240
                         **: **:*** **********:**.*************.***::**:**.:*:***:. *

AMR62839.1_ATCC49760     IKELKKKLNDGVSDPDEYLEIAKKVGYENLEPAQVQLAVQIEQAKQLEGAGEITWDIVKG     300
YobL_NCIB3610            IKELKKKLNDGVSDPDEYLEIAKKVGYENLEPTQVQLAVQIEQAKQLEGAGEITWDIVKG     300
AII35881.1_TO-A          IKELKKKLNDGVSDPDEYLEIAKKVGYENLEPTQVQLAVQIEQAKQLEGAGEITWDIVKG     300
AGA23389.1_BSP1          IKELKKKLNDGVSDPDEYLEIAKKVGYENLEPAQVQLAVQIEQAKQLEGAGEITWDIVKG     300
BAI85563.1_BEST195       IKELKKKLNDGVSDPVEYLEIAKKVGYENLEPAQVQLAVQIEQAKQLEGAGEITWDIVKG     300
AGI29216.1_BAB-1         IKELKKKLNDGVSDPDEYLEIAKKVGYENLEPAQVQLAVQIEQAKQLEEAGEVTWDIVKG     300
AKD35310.1_HJ5           IKELKKKLNDGVSDPDEYLEIAKKVGYENLEPAQVQLAVQIEQAKQLEEAGEVTWDIVKG     300
AGE63731.1_XF-1          IKELKKKLNDGVSDPDEYLEIAKKVGYENLEPAQVQLAVQIEQAKQLEEAGEVTWDIVKG     274
YokI_NCIB3610            IKELKKKLNDGVSDPDEYLEIAKKVGYENLEPAQVQLAVQIEQAKQLEGAGEITWDIVKG     300
AEP89572.1_RO-NN-1       IKDLKRQLV-KVTDPDEYLKIAKKIGYENLEPEQQVYFRQLEELQQK-------AEIGKG     292
BAI84001.1_BEST195       IKDLKKQLV-KVTDPDEYLKIAKKIGYENLEPEQQVYFRQLEELQQK-------AEIGKG     292
                         **:**::*   *:** ***:****:******* *     *:*: :*         :* **
```

S4 Fig B continued

B

```
AMR62839.1_ATCC49760      VGVGLYDVGKDTVTGIWDFITDPGETLSALGNAVIHPVKTYDAISAAIEESYQKDMVNGD     360
YobL_NCIB3610             VGVGLYDVGKDTVTGIWDFITDPGETLSALGNAAMHPVKTYDAISAAIEESYQKDMVNGD     360
AII35881.1_TO-A           VGVGLYDVGKDTVTGIWDFITDPGETLSALGNAAMHPVKTYDAISAAIEESYQKDMVNGD     360
AGA23389.1_BSP1           VGVGLYDVGKDTVTGLWDFITDPGETLSALGNAVIHPVKTYDAISAAIEESYQKDMVNGD     360
BAI85563.1_BEST195        VGVGLYDVGKDTVTGIWDFITDPGETLSALGNAVIHPVKTYDAISAAIEESYQKDMVNGD     360
AGI29216.1_BAB-1          VGVGLYDVGKDTVTGIWDFITDPGETLSALGNAVIHPVKTYDAISAAIEESYQKDMVNGD     360
AKD35310.1_HJ5            VGVGLYDVGKDTVTGIWDFITDPGETLSALGNAVIHPVKTYDAISAAIEESYQKDMVNGD     360
AGE63731.1_XF-1           VGVGLYDVGKDTVTGIWDFITDPGETLSALGNAVIHPVKTYDAISAAIEESYQKDMVNGD     334
YokI_NCIB3610             VGVGLYDVGKDTVTGLWDFITDPGETLSALGNAVIHPVKTYDAISAAIEESYQKDMVNGD     360
AEP89572.1_RO-NN-1        IAMGMYEAGKDTVMGLYQLARHPIETLSGTVNAALHPIDTYKIIAKDIEDTFQRDMINGD     352
BAI84001.1_BEST195        IAMGMYEAGKDTVMGLYQLARHPIESLSGTVNAALHPIDTYKIIAKDIEDTFQREMINGD     352
                          :.:*:*:.***** *:::: .* *:**.  **.:**:.**. *:  **:::*::*:***

AMR62839.1_ATCC49760      AYSRSRWVTYAVGSVAAAIVGTKGAGAINKADAAGKVINKA-----SQ--AGKKLKDVKL     413
YobL_NCIB3610             AYSRSRWVTYAIGSVAVAVVGTKGAGAINKADAAGKVINKA-----SQ--AGKKIKDVKI     413
AII35881.1_TO-A           AYSRSRWVTYAIGSVAVAVVGTKGAGAINKADAAGKVINKA-----SQ--AGKKIKDVKI     413
AGA23389.1_BSP1           AYSRSRWVTYAIGSVAAAVVGTKGAGAINKADAAGKVINKA-----SQ--AGKKIKDVKI     413
BAI85563.1_BEST195        AYSRSRWVTYAVGSVAVAVFGTKGAGAINKADAAGKVINKA-----SQ--AGKKIKDVKI     413
AGI29216.1_BAB-1          AYSRSRWVTYAVGSVAVAVFGNKGAGAINKADAAGKVINKA-----SQ--AGKKIKDVKI     413
AKD35310.1_HJ5            AYSRSRWVTYAVGSVAVAVFGTKGAGAINKADAAGKVINKA-----SQ--AGKKIKDVKI     413
AGE63731.1_XF-1           AYSRSRWVTYAVGSVAVAVFGTKGAGAINKADAAGKVINKA-----SQ--AGKKIKDVKI     387
YokI_NCIB3610             AYSRSRWVTYAIGSVAAAIVGTKGAGAINKADAAGKVINKA-----SQ--AGKKIKDVKI     413
AEP89572.1_RO-NN-1        SYSRAKWVSYVGSTVVLAIVGPKGIDKVSKVAKAGSKVAAIKALEASKAGIKKGIEYVKI     412
BAI84001.1_BEST195        SHSRAKWVSYVGSTVVLAIVGPKGIDKVSKVAKAGSKVAALKTLEVSKTGIKKGIEYVKI     412
                          ::**::**:*.  .:*. *:.* ** . :.*.  **. :        *:    * :: **:

AMR62839.1_ATCC49760      PDLLPYNPKNKL-ALADNVPYNVVDSQNLKNELLTNAKKLPDG----TRKPFTGQKIIP-     467
YobL_NCIB3610             PDLLPYNPKYKL-ALADNVPYNVVDSQNLKNELLTNAKKIPDG----TRKPFTGQKKSP-     467
AII35881.1_TO-A           PDLLPYNPKYKL-ALADNVPYNVVDSQNLKNELLTNAKKIPDG----TRKPFTGQKKSP-     467
AGA23389.1_BSP1           PDLLPYNPKYDF-ALADNVPYNVVDSQSLKNELLTNAKKIPDG----TRKPFTGQKISP-     467
BAI85563.1_BEST195        PDLLPYNPKYDF-PLADNVPYNVVDSQSLKNELLTNAKKIPDG----TRKPFTGQKISP-     467
AGI29216.1_BAB-1          PDLLPYNPKYDF-ALADNVPYNVVDSQSLKNELLTNAKKIPDG----TRKPFTGQKISP-     467
AKD35310.1_HJ5            PDLLPYNPKYDF-ALADNVPYNVVDSQSLKNELLTNAKKIPDG----TRKPFTGQKISP-     467
AGE63731.1_XF-1           PDLLPYNPKYDF-ALADNVPYNVVDSQSLKNELLTNAKKIPDG----TRKPFTGQKISP-     441
YokI_NCIB3610             PDLLPYNPKYDL-AMAGDVPYNVVDGENLKNQLMSFAKGSD-K----EVKPFDVVDYRPS     467
AEP89572.1_RO-NN-1        PNVFEQ--HYAMAGGSGTFPFNVIDGENYKSSALEIFKTSSTVQGLKKAKPHEVVNELK-     469
BAI84001.1_BEST195        PSVFEQ--QFAMAGGSGTFPFNVLDGENYKNSALEIFKNSSTVQGLKKAKPHEVVNELK-     469
                          *.::    :  :   :. .*:**:*.:. *.. :   *           **.   .

AMR62839.1_ATCC49760      ------------PWLNKEKYEAYEIEGKVKAK----GKVKDVSRRVYTMKDIDINQKTEF     511
YobL_NCIB3610             ------------PWLNKEKYDAYEIEGKVKAK----GKVKDVSRRVYTMKDIDINQKTEF     511
AII35881.1_TO-A           ------------PWLNKEKYDAYEIEGKVKAK----GKVKDVSRRVYTMKDIDINQKTEF     511
AGA23389.1_BSP1           ------------PWLNKEKYDAYEIEGKVKAK----GKVKDVSRRVYTMKDIDINQKTEF     511
BAI85563.1_BEST195        ------------PWLNKEKYDAYEIEGKVKAK----GKVKDVSRRVYTMKDIDINQKTEF     511
AGI29216.1_BAB-1          ------------PWLNKEKYEAYEIEGKVKAK----GKVKDVSRRVYTMKDIDINQKTEF     511
AKD35310.1_HJ5            ------------PWLNKEKYEAYEIEGKVKAK----GKVKDVSRRVYTMKDIDINQKTEF     511
AGE63731.1_XF-1           ------------PWLNKEKYEAYEIEGKVKAK----GKVKDVSRRVYTMKDIDINQKTEF     485
YokI_NCIB3610             NSPLENHHGVMDVWAKHN-VPNYVSRGSNTPTVALTKEQHNATKKVYREWL---------     517
AEP89572.1_RO-NN-1        ------------TF----QSRKYTFGGQS-FLIDKRGMKHILERHHPNLWDGSIKSQQSF     512
BAI84001.1_BEST195        ------------TF----QSRKYTFGGQS-FLIDKRGMKHILERHHPNLWDGSIKSQQSF     512
                                                         toxin

AMR62839.1_ATCC49760      GVTNL----------QLMKNGNAPYAKDGTQINLHHL----IQEEPGPMLE---IPNSLH     554
YobL_NCIB3610             GVTNL----------QLMKNGNAPYAKDGTQINLHHL----IQEEPGPMLE---IPNSLH     554
AII35881.1_TO-A           GVTNL----------QLMKNGNAPYAKDGTQINLHHL----IQEEPGPMLE---IPNSLH     554
AGA23389.1_BSP1           GVTNL----------QLMKNGNAPYAKDGTQINLHHL----IQEEPGPMLE---IPNSLH     554
BAI85563.1_BEST195        GVTNL----------QLMKNGNAPYAEDGTQINLHHL----IQEEPGPMLE---IPNSLH     554
AGI29216.1_BAB-1          GVTNL----------QLMKNGNAPYAKDGTQINLHHL----IQEEPGPMLE---IPNSLH     554
AKD35310.1_HJ5            GVTNL----------QLMKNGNAPYAKDGTQINLHHL----IQEEPGPMLE---IPNSLH     554
AGE63731.1_XF-1           GVTNL----------QLMKNGNAPYAKDGTQINLHHL----IQEEPGPMLE---IPNSLH     528
YokI_NCIB3610             ----------------FEKTGK----KVGGKVNWKEVSPREIQELTEKMFDAANVPKEAR     557
AEP89572.1_RO-NN-1        LNKEMTVNDVADAIESIMKQNREELTKKGTKFSYQIRGTYEGQ-----------------     555
BAI84001.1_BEST195        LNKEMTVNDVADAIESIMKQNREELTKKGTKFSYQIRGSYEGQ-----------------     555
                                    toxin                        *

AMR62839.1_ATCC49760      TKYSDIIHKLKSDGESFRNDKVLKAQYESFRS--------------    586
YobL_NCIB3610             TKYSDVIHQLKSDGESFRNDKVLKAQYESFRKRYWKWRAKQFENEN    600
AII35881.1_TO-A           TKYSDVIHQLKSDGESFRNDKVLKAQYESFRKRYWKWRAKQFENEN    600
AGA23389.1_BSP1           TKYSDVIHQLKSDGESFRNDKVLKAQYESFRKRYWKWRAKQFENEN    600
BAI85563.1_BEST195        TKYSDVIHQLKSDGESFRNDKVLKAQYESFRKRYWKWRAKQFENEN    600
AGI29216.1_BAB-1          TKYSDVIHQLKSDGESFRNDKVLKAQYESFRKRYWKWRAKQFENEN    600
AKD35310.1_HJ5            TKYSDVIHQLKSDGESFRNDKVLKAQYESFRKRYWKWRAKQFENEN    600
AGE63731.1_XF-1           TKYSDVIHQLKSDGESFRNDKVLKAQYESFRKRYWKWRAKQFENEN    574
YokI_NCIB3610             QQYYN-----------------------AFNQY--NFRK-------    571
AEP89572.1_RO-NN-1        -KY---------------------------VVGFQKGRVGQFYPEK    573
BAI84001.1_BEST195        -QY---------------------------VVGFQKGRVGQFYPEK    573
                           :*
```

S4 Fig B

C

```
                             LXG
AII36371.1_TO-A       --MKVFEAKTLLTEAEKRAQEYKDLKSKMVKLKKAFKAVADLDDSEFSGKGANNIKSFYE    58
YqcG_NCIB3610         --MKVFEAKTLLTEAEKRAQEYKDLKSKMVKLKKAFKAVADLDDSEFSGKGANNIKSFYE    58
ADV93340.1_BSn5       --MKVFEAKTLLTEAEKRAKEYKDLKSKMVKLKKAFKAVADLDDSEFSGKGANNIKSFYE    58
AEP87466.1_TU-B-10    ------------------------------------------MIAEFSGKGANNIKSFYE    18
AEP91632.1_RO-NN-1    --MKVFEAKTLLSEAEKRAKEYKDLKSKMVKLKKAFKAVADLDDSEFSGKGANNIKSFYE    58
BAI86935.2_BEST195    MIMKVFEAKTLLSEAENRAKEYKDLKSKMIKLKKAFKAVADLDDSEFSGKGANNIKSFYE    60
                                                                 :****************

                             LXG
AII36371.1_TO-A       DQAGIADQWIDLIEMKISFLTSIPGFLEDANLSDAYIEETFLAHELANAYTKSKSIMSEQ    118
YqcG_NCIB3610         DQAGIADQWIDLIEMKISFLTSIPGFLEDANLSDAYIEETFLAHELANAYTKSKSIMSEQ    118
ADV93340.1_BSn5       DQAGIADQWIDLIEMKISFLTSIPGFLEDANLSDAYIEETFLAHELANASTKSKSIMSEQ    118
AEP87466.1_TU-B-10    DQAGIADQWIDLIEMKISFLTSIPGFLEDANLSDAYIEETFLAHELANASTKSKSIMSEQ    78
AEP91632.1_RO-NN-1    DQAGIADQWIDLIEMKISFLTSIPGFLEDANLSDAYIEETFLAHELANAYTKSKSIMSEQ    118
BAI86935.2_BEST195    DQAGIADQWIDLIEMKISFLTSIPGILEDSSLSDAYIEESFLEHELANANSKSKSIMSEQ    120
                      *************************:***. ********:** ****** :*********

                             LXG
AII36371.1_TO-A       KKAMKDILNDINDILPLDLFSTETFKNELSSAEKKRKEAIEKMDEVDQNLTSEYGLSEAN    178
YqcG_NCIB3610         KKAMKDILNDINDILPLDLFSTETFKNELSSAEKKRKEAIEKMDEVDQNLTSEYGLSEAN    178
ADV93340.1_BSn5       KKAMQDILNDINDILPLDLFSTETFKNELSSAEKKRKEAIEKMDEVDENLTSEYGLSEAN    178
AEP87466.1_TU-B-10    KKAMQDILNDINDILPLDLFSTETFKNELSSAEKKRKEAIEKMDEVDENLTSEYGLSEAN    138
AEP91632.1_RO-NN-1    KKAMKDILNDINDILPLDLFSTETFKNELSSAEKKRTEAIEKMDEVDENLTSEYGLSEAN    178
BAI86935.2_BEST195    KKAIKDILNEIHDVLPLDVFSTEDFKTELSSAEKKRKNTVEKISEVDEDLTSEYALSEAN    180
                      ***::****:*:*:****:**** **.*********.:::**:.***::*****.*****

                   LXG
AII36371.1_TO-A       EQMIQADYQALMNATAKGKSASPIHYNAKAYRDSEIHKMTEDVKKQSTDYISFKDQQAEQ    238
YqcG_NCIB3610         EQMIQADYQALMNATAKGKSASPIHYNAKAYRDSEIHKMTEDVKKQSTDYISFKDQQAEQ    238
ADV93340.1_BSn5       EQMIQADYQALMNATAKGKSASPIHYNAMAYRDSEIHKMTEDVKKQSTDYISFKDQQAEQ    238
AEP87466.1_TU-B-10    EQMIQADYQALMNATAKGKSTSPIHYNAKAYRDSEIHKMTEDVKKQSTDYISFKDQQTEQ    198
AEP91632.1_RO-NN-1    EQMIQADYQALMNATAKGKSASPIHYNAKAYRDSEIHKMTEDVKKQSTDYISFKDQQAEQ    238
BAI86935.2_BEST195    EQMIQADYQALINATAKGKSASPIHYNAKAYRDSEIHKMTEDVKKQSTDYIAFKDQQAEQ    240
                      ***********:********:******* **********************:*****:**

AII36371.1_TO-A       RRIAKEQEELANRPWYEKSWDAVCNFTGEVSGYYDYKRAADGVDPVTGEKLTAGQRVAAG    298
YqcG_NCIB3610         RRIAKEQEELANRPWYEKSWDAVCNFTGEVSGYYDYKRAADGVDPVTGEKLTAGQRVAAG    298
ADV93340.1_BSn5       RRIAKEQEELANRPWYEKSWDAVCNFTGEVSGYYDYKRAADGVDPVTGEKLTAGQRVAAG    298
AEP87466.1_TU-B-10    RRIAKEQEELANRPWYEKSWDAVCNFTGKVSGYYDYKRAADGVDPVTGEKLTAGQRVAAG    258
AEP91632.1_RO-NN-1    RRIAKEQEELANRPWYEKSWDAVCNFTGEVSGYYDYKRAADGVDPVTGEKLTAGQRVAAG    298
BAI86935.2_BEST195    RRLAKEQEELANRPWYEKTWDVVCNFTGEVSGYYDYKRAADGVDPVTGEKLTEGQRVAAG    300
                      **:***************:**.******:*********************** *******

AII36371.1_TO-A       AMAAAGYIPIVGWAGKLAKGGKAVYSTSKALYRADKALDVYKTPKTFHALQNSSKGLYGL    358
YqcG_NCIB3610         AMAAAGYIPIVGWAGKLAKGGKAVYSTSKALYRADKALDVYKTPKTFHALQNSSKGLYGL    358
ADV93340.1_BSn5       AMAAAGYIPIVGWAGKLAKGGKAVYSTSKALYRADKALDVYKTPKTFHALQNSSKGLYGL    358
AEP87466.1_TU-B-10    AMAAAGYIPIVGWAGKLAKGGKAVYSTSKALYKADKALDVYKTPKTFHALQNSSKGLYGL    318
AEP91632.1_RO-NN-1    AMAAAGYIPIVGWAGKLAKGGKAVYSTSKVLYRADKALDVYKTPKTFHALQNSSKGLYGL    358
BAI86935.2_BEST195    AMAAAGYVPIVGWAGKLAKGGKAVYSTSKALYTADKALDVYKTPQTFHALQNSSKGLYGL    360
                      *******:*********************.** ***********:***************

AII36371.1_TO-A       ASANGFSEAITGRDMFGNKVSKERQEQSLSGAMAMLVPFGARGINKKLNAKSSSRVSEAS    418
YqcG_NCIB3610         ASANGFSEAITGRDMFGNKVSKERQEQSLSGAMAMLVPFGARGINKKLNAKSSSRVSEAS    418
ADV93340.1_BSn5       ASANGFSEAITGRDMFGNKVSKERQEQSLSGAMAMLVPFGARGINKKLNAKSSSRVSETS    418
AEP87466.1_TU-B-10    ASANGFSEAITGHDMFGNKVSDEQRQNSINLALSAFVPFGVHGVSGKLNAKSKSTSGIAS    378
AEP91632.1_RO-NN-1    ASANGFSEAITGHDMLGNKVSDEQRQNSINLALSAFVPFGVHGVSGKLNAKSKSTSGIAS    418
BAI86935.2_BEST195    ASANGFSEAITGRDMFGNKISKEQQHNSINAALAVFMPFGAKGFSGKTGLKASSSTETV-    419
                      ************:**:***:*.*::.:*:.  *:: ::***.:*..  *  .  *:.*     .

                                                                     toxin
AII36371.1_TO-A       TNTSKKPK--------VPKTYKRPTYFRKGVRDKVWENAKDSTGS-VKDPLTK-QVMKKD    468
YqcG_NCIB3610         TNTSKKPK--------VPKTYKRPTYFRKGVRDKVWENAKDSTGS-VKDPLTK-QVMKKD    468
ADV93340.1_BSn5       TNTSKKPK--------VPKTYKRPTYFRKGVRDKVWENAKDSTGS-VKDPLTK-QVMKKD    468
AEP87466.1_TU-B-10    GANKNYHESRSASLREIKRQLNIPRTQQPA-SQKMV-PLTDSNGNRILNEKKQ-PVMTRE    435
AEP91632.1_RO-NN-1    GSNKNYHESRSASLREIKRQLNIPRTQQPA-SQKMV-PLTDSNGNRILNEKKQ-PVMTRE    475
BAI86935.2_BEST195    ---TQVTVSRKGAFKEAKRDAGIPRAQQPESINRVEMRTAPHEGGRVIKDKNGKIIRTRE    476
                         .:             :    *   :     :::          *. :  .   .   :  .::

                                        toxin
AII36371.1_TO-A       EPWD-MGH--------KPGYEFRKHQQSAMERNISRKQFLDEHNNPDHYQPELPSSNRSH    519
YqcG_NCIB3610         EPWD-MGH--------KPGYEFRKHQQSAMERNISRKQFLDEHNNPDHYQPELPSSNRSH    519
ADV93340.1_BSn5       EPWD-MGH--------KPGYEFRKHQQSAMERNISRKQFLDEHNNPDHYQPELPSSNRSH    519
AEP87466.1_TU-B-10    LTYE-INGKKIVIQDHSHGHDFGEG-------G--------IGNQPSHHNVRPENNTRNG    479
AEP91632.1_RO-NN-1    LTYE-INGKKIVIQDHSHGHDFGEG-------G--------IGNQPSHHNVRPENNTRNG    519
BAI86935.2_BEST195    YTFTNNKGEKIIIQDHSAGHEK--------------------GGQGPHFNVRPIDNTRTG    516
                        :              . *::                           .:   *.:  .   ...*.

AII36371.1_TO-A       KGEDMTDDYFGD-----     531
YqcG_NCIB3610         KGEDMTDDYFGD-----     531
ADV93340.1_BSn5       KGEDMTDDYFGD-----     531
AEP87466.1_TU-B-10    KVDGMEDHYYFDRRNRK     496
AEP91632.1_RO-NN-1    KVDGMEDHYYFDRRNRK     536
BAI86935.2_BEST195    KVPGTKEHYPFNK----     529
                      *  .  :.*  :
```

S4 Fig C

D

```
                                               LXG
YwqJ_NCIB3610            MSKVFESKSLIEEAKSRKKQYETLEEQLNTLKKAFQGVADLGDNFKGNGADNIKDFFQGQ    60
AKD36832.1_HJ5           MSKVFESKSLIEEAKSRKKQYETLEEQLNTLKKAFQGVADLGDNFKGKGADNIKDFFQGQ    60
BAO93629.1_BEST195       MSKVFESKSLIEEAKSRKKQYETLEEQLNTLKKAFQGVADLGDNFKGKGADNIKDFFQGQ    60
AGE65222.1_XF-1          MSKVFESKSLIEEAKSRKKQYETLEEQLNTLKKAFQGVADLGDNFKGKGADNIKDFFQGQ    60
ADV94423.1_BSn5          MSKVFESKSLIEEAKSRKKQYETLEEQLNTLKKAFQGVADLGDNFKGNGADNIKDFFQGQ    60
AGA21789.1_BSP1          MSKVFESKSLIEEAKSRKKQYETLEEQLNTLKKAFQGVADLGDNFKGKGADNIKDFFQGQ    60
AII38166.1_TO-A          MSKVFESKSLIEEAKSRKKQYETLEEQLNTLKKAFQGVADLGDNFKGKGADNIKDFFQGQ    60
AEP92663.1_RO-NN-1       MSKVFESKSLIEEAKSRKKQYETLEEQLNTLKKAFQGVADLGDNFKGKGADNIKDFFQGQ    60
AIC99919.1_OH131.1       MSKVFESKSLIEEAKSRKKQYETLEEQLNTLKKAFQGVADLGDNFKGKGADNIKDFFQGQ    60
AEP88550.1_TU-B-10       MSKVFESKSLIEEAKSRKKQYETLEEQLNTLKKAFQEVADLGDNFKGKGADNIKDFFQGQ    60
ADM39597.1_W23           MSKVFESKSLIEEAKSRKKQYETLEEQLNTLKKAFQEVADLGDNFKGKGADNIKDFFQGQ    60
AMR61191.1_ATCC49760     MSKVFESQSLIDEAEKRKKQYETFEEQLNTLKKAFQGVADLGDDFKGKGADNIKDFFQGQ    60
                         *******:***:**:.*******:************ ******:***:************

                                              LXG
YwqJ_NCIB3610            AEIVDSWLTLVSAQIAFLNGISGDIKDQELNDSYVETSFLDHELPNGDLKASEIVSAHKE    120
AKD36832.1_HJ5           AEIVDSWLTLVSAQIAFLNGISGDIKDQELNDSYVETSFLDHELPNGDLKASEIVSAHKE    120
BAO93629.1_BEST195       AEIVDSWLTLVSAQIAFLNGISGDIKDQELNDSYVETSFLDHELPNGDLKASEIVSAHKE    120
AGE65222.1_XF-1          AEIVDSWLTLVSAQIAFLNGISGDIKDQELNDSYVETSFLDHELPNGDLKASEIVSAHKE    120
ADV94423.1_BSn5          AEIVDSWLTLVSAQIAFLNGISGDIKDQELNDSYVETSFLDHELPNGDLKASEIVSAHKE    120
AGA21789.1_BSP1          AEIVDSWLTLVSAQIAFLNGISGDIKDQELNDSYVETSFLDHELPNGDLKASEIVSAHKE    120
AII38166.1_TO-A          AEIVDSWLTLVSAQIAFLNGISGDIKDQELNDSYVETSFLDHELPNGDLKASEIVSAHKE    120
AEP92663.1_RO-NN-1       AEIVDSWLTLVSAQIAFLNGISGDIKDQELNDSYVETSFLDHELPNGDLKASEIVSAHKE    120
AIC99919.1_OH131.1       AEIVDSWLTLVSAQIAFLNGISGDIKDQELNDSYVETSFLDHELPNGDLKASEMVSAHKE    120
AEP88550.1_TU-B-10       AEIVESWLTLVSAQIAFLNGISGDIKDQELNDSYVETSFLDHELSNGDLKASEIVSAHKE    120
ADM39597.1_W23           AEIVESWLTLVSAQIAFLNGISGDIKDQELNDSYVETSFLDHELPNGDLKASEIVSAHKE    120
AMR61191.1_ATCC49760     AEIVDSWLTLVSSQIAFLKGISGDIKDQELSDSYVETSFLDHELPNGDLKASEIVSAHKA    120
                         ****:*******:*****:***********.************* ********:*****

                                              LXG
YwqJ_NCIB3610            EIDSILSGISDIIDLDMYTLDDYADKMGDAQKIRRDTITAVDKLDESLTTEYQNLESLDN    180
AKD36832.1_HJ5           EIDSILSGISDIIDLDMYTLDDYADKMGDAQKTRRDTITAVDKLDESLTTEYQNLESLDN    180
BAO93629.1_BEST195       EIDSILSGISDIIDLDMYTLDDYADKMGDAQKIRRDTITAVDKLDESLTTEYQNLESLDN    180
AGE65222.1_XF-1          EIDSILSGISDIIDLDMYTLDDYADKMGDAQKTRRDTITAVDKLDESLTTEYQNLESLDN    180
ADV94423.1_BSn5          EIDSILSGISDIIDLDMYTLDDYADKMGDAQKIRRDTITAVDKLDESLTTEYQNLESLDN    180
AGA21789.1_BSP1          EIDSILSGISDIIDLDMYTLDEYADKMGDAQKIRRDTITAVDKLDESLTTEYQNLESLDN    180
AII38166.1_TO-A          EIDSILSGISDIIDLDMYTLDEYADKMGDAQKVRRDTITAVDKLDESLTTEYQNLESLDN    180
AEP92663.1_RO-NN-1       EIDSILSGISDIIDLDMYTLDDYADKMGDAQKIRRDTITAVDKLDESLTTEYQNLESLDN    180
AIC99919.1_OH131.1       EIDSILSGISDIIDLDMYTLDDYADKMGDAQKIRRDTITAVDKLDESLTTEYQNLESLDN    180
AEP88550.1_TU-B-10       EIDSILSGISDIIDLDMYTLDDYADKMGDAQKIRRETITAVDKLDESLTTEYQNLESLDN    180
ADM39597.1_W23           EIDSILSGISDIIDLDMYTLDDYADKMGDAQKIRRDTITAVDKLDESLTTEYQNLESLDN    180
AMR61191.1_ATCC49760     EIDSILSGISDIIDLDMYSLDNYADKMDNAQKVRRDTITAVDQLDESLTTEYQNLESLDN    180
                         *****************:**:*****.:*** **:******:*****************

                                   LXG
YwqJ_NCIB3610            AVLTKYSVLMQATSNGKSASPMYYDKKAFHSNEVYKSVIEVENQGTTYIDAKTQQAEARR    240
AKD36832.1_HJ5           AVLTKYSVLMQATSNGKSASPMYYDKKAFHSNEVYKSVIEVENQGTTYIDAKTQQAEARR    240
BAO93629.1_BEST195       AVLTKYSVLMQATSNGKSASPMYYDKKAFHSNEVYKSVIEVENQGTTYIDAKTQQAEARR    240
AGE65222.1_XF-1          AVLTKYSVLMQATSNGKSASPMYYDKKAFHSNEVYKSVIEVENQGTTYIDAKTQQAEARR    240
ADV94423.1_BSn5          AVLTKYSVLMQATSNGKSASPMYYDKKAFHSNEVYKSVIEVENQGTTYIDAKTQQAEARR    240
AGA21789.1_BSP1          AVLTKYSVLMQATSNGKSASPMYYDKKAFHSNEVYKSVIEVENQGTTYIDAKAQQAEARR    240
AII38166.1_TO-A          AVLTKYSVLMQATSNGKSASPMYYDKKAFHSNEVYKSVIEVENQGTTYIDAKAQQAEARR    240
AEP92663.1_RO-NN-1       AVLTKYSVLMQATSNGKSASPMYYDKKAFHSNEVYKSVIEVENQGTTYIDAKTQQAEARR    240
AIC99919.1_OH131.1       AVLTKYSVLMQATSNGKSASPMYYDKKAFHSNEVYKSVIEVENQGTTYIDAKTQQAEARR    240
AEP88550.1_TU-B-10       AVLTKYSVLMQATSNGKSASPMYYDKKAFHSNEVYKSVIEVEKQGTTYIDAKAQQAEARR    240
ADM39597.1_W23           AVLTKYSVLMQATSNGKSASPMYYDKKAFHSNEVYKSVIEVEKQGTTYIDAKAQQAEARR    240
AMR61191.1_ATCC49760     AVLSKYSVLMQATSNGKSATPMYYDKKAFHSNEVYKSVIEVEKQGTSYIEAKEQQAEARR    240
                         ***:***************:**********************:***:**:** *******

YwqJ_NCIB3610            LQEKAEEEANKPWYEKTWDGVCNFTGEVTGYYDYKRATEGVDPVTGEKLSTAERVTAGAM    300
AKD36832.1_HJ5           LQEKAEEEANKPWYEKTWDGVCNFTGEVTGYYDYKRATEGVDPVTGEKLSTAERVTAGAM    300
BAO93629.1_BEST195       LQEKAEEEANKPWYEKTWDGVCNFTGEVTGYYDYKRATEGVDPVTGEKLSTAERVTAGAM    300
AGE65222.1_XF-1          LQEKAEEEANKPWYEKTWDGVCNFTGEVTGYYDYKRATEGVDPVTGEKLSTAERVTAGAM    300
ADV94423.1_BSn5          LQEKAEEEANKPWYEKTWDGVCNFTGEVTGYYDYKRATEGVDPVTGEKLSTAERVTAGAM    300
AGA21789.1_BSP1          LQEKAEEEANKPWYEKTWDGVCNFTGEVTGYYDYKRATEGIDPVTGEKLSTAERVTAGAM    300
AII38166.1_TO-A          LQEKAEEEANKPWYEKTWDGVCNFTGEVTGYYDYKRATEGIDPVTGEKLSTAERVTAGAM    300
AEP92663.1_RO-NN-1       LQEKAEEEANKPWYEKTWDGVCNFTGEVTGYYDYKRATEGVDPVTGEKLSTAERVTAGAM    300
AIC99919.1_OH131.1       LQEKAEEEANKPWYEKTWDGVCNFTGEVTGYYDYKRATEGVDPVTGEKLSTAERVTAGAM    300
AEP88550.1_TU-B-10       LQEKAEEEANKPWYEKTWDGVCNFTGEVTGYYDYKRATEGVDPVTGEKLSTAERVTAGAM    300
ADM39597.1_W23           LQEKAEEEANKPWYEKTWDGVCNFTGEVTGYYDYKRATEGVDPVTGEKLSTAERVTAGAM    300
AMR61191.1_ATCC49760     LQEKAEEEANKPWYEKTWDGICTFTGEVSGYYDYKRATEGVDPVTGEKLSAAERVTAGAM    300
                         ********************:*.*****:***********:*********:*********
```

S4 Fig D continued

D

```
YwqJ_NCIB3610          AAAGFIPVVGWAGRAFKGGKAIYKTGKAAIAAEHALDAYKTGKSLDILKMTEMGAYGLVA    360
AKD36832.1_HJ5         AAAGFIPVVGWAGRAFKGGKAIYKTGKAAIAAEHALDAYKTGKSLDILKMTEMGAYGLVA    360
BAO93629.1_BEST195     AAAGFIPVVGWAGRAFKGGKAIYKTGKAAIAAEHALDAYKTGKSLDILKMTEMGAYGLVA    360
AGE65222.1_XF-1        AAAGFIPVVGWAGRAFQGGKAIYKTGKAAIAAEHALDAYKTGKSLDILKMTEMGAYGLVA    360
ADV94423.1_BSn5        AAAGFIPVVGWAGRAFKGGKAIYKTGKAAIAAEHALDAYKTGKSLDILKMTEMGAYGLVA    360
AGA21789.1_BSP1        AAAGFIPVVGWAGRAFKGGKAIYKTGKAAIAAEHALDAYKTGKSLDILKMTEMGAYGLVA    360
AII38166.1_TO-A        AAAGFIPVVGWAGRAFKGGKAIYKTGKAAIAAEHALDAYKTGKSLDILKMTEMGAYGLVA    360
AEP92663.1_RO-NN-1     AAAGFIPVVGWAGRAFKGGKAIYKTGKAAIAAEHALDAYKTGKSLDILKMTEMGAYGLVA    360
AIC99919.1_OH131.1     AAAGFIPVVGWAGRAFKGGKAIYKTGKAAIAAEHALDAYKTGKSLDILKMTEMGAYGLVA    360
AEP88550.1_TU-B-10     AAAGFIPVVGWAGRAFKGGKAIYKTGKAAIAADHALDAYKTGKSLDILKMSEMGAYGLVA    360
ADM39597.1_W23         AAAGFIPVVGWAGRAFKGGKAIYKTGKAAIAADHALDAYKTGKSLDILKMSEMGAYGLVA    360
AMR61191.1_ATCC49760   AAAGFIPVVGWAGRAFKGGKAIYKTGKAAITAEHALDAYKTGKSLDILKMSEMGAYGLIA    360
                       ****************:*************:*:*****************:*******:*

YwqJ_NCIB3610          SNGFSEAVTGRDMFGNKVSEEKRKQGALEAITIIGGAGL-AHYFDRLYQKNAPYVNKVSN    419
AKD36832.1_HJ5         SNGFSEAVTGRDMFGNKVSEEKRKTGALEAALSLVGAGALAKSVDNTL----SMGRNLDK    416
BAO93629.1_BEST195     SNGFSEAVTGRDMFGNKVSEEKRKTGALEAALSLVGAGALAKSVDNTL----SMGRNLDK    416
AGE65222.1_XF-1        SNGFSEAVTGRDMFGNKVSEEKRKQGALEAVLSLVGAGALAKHIDKGI----PLATHSKV    416
ADV94423.1_BSn5        SNGFSEAVTGRDMFGNKVSEEKRKQGALEAALSLVGAGALAKHIDKGI----PLATHSKV    416
AGA21789.1_BSP1        SNGFSEAVTGRDMFGNKVSEEKRKQGALEAALSLVGAGALAKHIDKGI----PLATHSKV    416
AII38166.1_TO-A        SNGFSEAVTGRDMFGNKVSEEKRKQGALEAALSLVGAGALAKHIDKGI----PLATHSKV    416
AEP92663.1_RO-NN-1     SNGFSEAVTGRDMFGNKVSEEKRKQGALEAALSLVGAGALAKHIDKGI----PLATHSKV    416
AIC99919.1_OH131.1     SNGFSEAVTGRDMFGNKVSEEKRKQGALEAALSLVGAGALAKHIDKGI----PLATHSKV    416
AEP88550.1_TU-B-10     SNGFSEAVTGRDMFGNKVSEEKRKQGALEAALSLVGAGALAKHINKGI----PLATHSKV    416
ADM39597.1_W23         SNGFSEAVTGRDMFGNKVSEEKRKQGALEAALSLVGAGALAKHINKGI----PLATHSKV    416
AMR61191.1_ATCC49760   SNGFSEAVTGRDMFGNKVSDEKRKTGALEAALSLVGAGALAKHIDKGI----PLATHTKV    416
                       ******************:****  *****   :  *.*  *: .:.          : .
                                                           toxin
YwqJ_NCIB3610          ESLISNIAKTTEEKQTRLQYLRNKH----GVLSKEDLH-----HRINLRAEVLNELSRIK    470
AKD36832.1_HJ5         SY-----S---FSK-----IKQSGHINKEQIVTSTGHTFDLKPEKKQLYTKK------VN    457
BAO93629.1_BEST195     SY-----S---FSK-----IKQSGHINKEQIVTSTGHTFDLKPEKKQLYTKK------VN    457
AGE65222.1_XF-1        QT-----ANKTLEKVKQFKVPTNVRVYAERPVTPDGFTFGIPTVRVDVEKTA------VK    465
ADV94423.1_BSn5        QK-----ANKTLEKVKQFKVPTNVRVYAERPVTPDGFTFGIPTVRVDVEKTA------VK    465
AGA21789.1_BSP1        QK-----ANKTLEKVKQFKVPTNVRVYAERPVTPEGFTFGIPTVRVDVEKTA------VK    465
AII38166.1_TO-A        QK-----ANKTLEKVKQFKVPTNVRVYAERPVTPDGFTFGIPTVRVDVEKTA------VK    465
AEP92663.1_RO-NN-1     QK-----ANKTLEKVKQFKVPTNVRVYAERPVTPEGFTFGIPTVRVDVEKTA------VK    465
AIC99919.1_OH131.1     QK-----ANKTLEKVKQFKVPTNVRVYAERPVTPEGFTFGIPTVRVDVEKTA------VK    465
AEP88550.1_TU-B-10     QK-----ANKTLEKVKQFKVPTNVRVYAERPVTPDGLTFGFPTVRVDVETKS------VK    465
ADM39597.1_W23         QK-----ANKTLEKVKQFKVPTNVRVYAERPVTPDGLTFGFPTVRVDVGTKS------VK    465
AMR61191.1_ATCC49760   QK-----ANTILEKAKQFKVPTNVRVYAERPVTTNGFTFGFPTVRVDVGTKS------VK    465
                       .      :    .*        . :      ::  .        : ::          ::
                                                toxin
YwqJ_NCIB3610          SSGLTKKQ--RGPAVA----------GVLDKKTGNYYFGINNIDGKPPKVLHPLIHDRIV    518
AKD36832.1_HJ5         NDKLQSSKKAVDPVEQSVSYYRVQGGGAGNK-TSQYRIKVNS-DGS---IAIPI-KNADL    511
BAO93629.1_BEST195     NDKLQSSKKSVDPVEQSVSYYRVQGGGAGNK-TSQYRIKVNS-NGS---IVIPI-KNADL    511
AGE65222.1_XF-1        DLGI----------------VKMVSDGGRK-GSSHS---------P---NISEN-HDKVA    495
ADV94423.1_BSn5        DLGI----------------VKMVSDGGRK-GSSHS---------P---NISKN-HDKVA    495
AGA21789.1_BSP1        DLGI----------------VKMVSDGGRK-GSSHS---------P---NISKN-HDKVA    495
AII38166.1_TO-A        DLGI----------------VKMVSDGGRK-GSSHS---------P---NISKN-HDKVA    495
AEP92663.1_RO-NN-1     DLGI----------------VKMVSSGKGIKGTGKS---------S---NKNPL-P-SWL    495
AIC99919.1_OH131.1     DLGI----------------VKMVSSGKGIKGTGKS---------S---NKNPL-P-SWL    495
AEP88550.1_TU-B-10     DLGI----------------VKMMGKWSNGGEYDRIR---GY-TGR---DLSKY-DNKYL    501
ADM39597.1_W23         DLGI----------------VKMMGKWSNGGEYDRIR---GY-TGR---DLSKY-DNKYL    501
AMR61191.1_ATCC49760   DLGI----------------VKMASGPSKKPQ--------------------------WV    483
                       .  :
                                                toxin
YwqJ_NCIB3610          N--MPTELK-EGYIKTSGAGSHAE-------VNALNEALLQRPDADLKDLMV--------    560
AKD36832.1_HJ5         NISAYNLEHAKYYRDVARPGGEIVEFKVPK---EVDDLIKE-------------------    549
BAO93629.1_BEST195     NISAYNLEHAKYYRDVARPGGEIVEFKVPK---EVDDLIKE-------------------    549
AGE65222.1_XF-1        H----------------------DGYYG---RKEFRTMLNNASFTPHGHKHVKAKTPEEA    530
ADV94423.1_BSn5        H----------------------DGYYG---RKEFRTMLNNASFTPHGHKHVKAKTPEEA    530
AGA21789.1_BSP1        H----------------------DGYYG---RKEFRTMLNNASFTPHGHKHVKAKTPEEA    530
AII38166.1_TO-A        H----------------------DGYYG---RKEFRTMLNNASFTPHGHKHVKAKTPEEA    530
AEP92663.1_RO-NN-1     K----------------------ERWEA---GNNFNKE--NRPRYPYNEVELEAKEAG-G    527
AIC99919.1_OH131.1     K----------------------ERWEA---GNNFNKE--NRPRYPYNEVELEAKEAG-G    527
AEP88550.1_TU-B-10     ID--PRLVVEMNFKGKGKPGTNAAGWERN-AKKFFNTLLKSNPEFWSAENTAKIK-----    553
ADM39597.1_W23         ID--PRLVVEMNFKGKGKPGTNAAGWERN-AKKFFNTLLKSNPEFWSAENTAKIK-----    553
AMR61191.1_ATCC49760   QK--PEDMTQNDYVAHNG-----NNYSKKKRTEEYNAAIADR------------------    518
                                                               .
```

S4 Fig D continued

D

```
                           toxin
YwqJ_NCIB3610          --YVVSARK--------------------INKKMPE-----------------------GV    576
AKD36832.1_HJ5         ---TVIDQ--YGYTKNPRNQG--R--------TAPK---LVDPT----------TPGVSY    581
BAO93629.1_BEST195     ---TVIDQ--YGYTKNPRNQG--R--------TAPK---LVDPT----------TPGVSY    581
AGE65222.1_XF-1        KRFSMTGKKAAQYLPDVNNKALEK--------KALLKGHIIDNGNNNYYFIYDAGKTVGY    582
ADV94423.1_BSn5        KRFSMTGKKAAQYLPDVNNKALEK--------EALLKGHIIDNGNNNYYFIYDAGKTVGY    582
AGA21789.1_BSP1        KRFSMTGKKAAQYLPDVNNKALEK--------EALLKGHIIDNGNNNYYFIYDAGKTVGY    582
AII38166.1_TO-A        KRFSMTGKKAAQYLPDVNNKALEK--------EALLKGHIIDNGNNNYYFIYDAGKTVGY    582
AEP92663.1_RO-NN-1     KKYVV-----DSYSPNKE--IVSR--------KYTQLSDVQEKT----ALSYLNEITKKY    568
AIC99919.1_OH131.1     KKYVV-----DSYSPNKE--IVSR--------KYTQLSDVQEKT----ALSYLNEITKKY    568
AEP88550.1_TU-B-10     ----------RGRVPVVDEQFI-K--------HFP------------QYADYLKDPMRHH    582
ADM39597.1_W23         ----------RGRVPVVDEQFI-K--------HFP------------QYADYLKDPMRHH    582
AMR61191.1_ATCC49760   --EKIIGDNSNGYRLKEPTKWMENRGMVQIKERLKRKS-WTDNEGFNW----------QW    565

                       toxin
YwqJ_NCIB3610          PMPRCPHCEY----ITQNTNYIPEALKYGK------------------------------    602
AKD36832.1_HJ5         ELPAEPWIEWLEEYGHSAKKIE--------------------------------------    603
BAO93629.1_BEST195     ELPAEPWIEWLEEYGHSAKKIE--------------------------------------    603
AGE65222.1_XF-1        DLGT--PTSWIRAEF-SGGEYHGHPIAGSRLDKYL-----------KQLGIDK-------    621
ADV94423.1_BSn5        DLGT--PTSWIRAEF-SGGEYHGHPIAGSRLDKYL-----------KQLGIDK-------    621
AGA21789.1_BSP1        DLGT--PTSWIRAEF-SGGEYHGHPIAGSRLDKYL-----------KQLGIDK-------    621
AII38166.1_TO-A        DLGT--PTSWIRAEF-SGGEYHGHPIAGSRLDKYL-----------KQLGIDK-------    621
AEP92663.1_RO-NN-1     SSG---------SKI-SNGPFNPKALKGGRLKGELILEVPVQNKAIPQKILDEATKNRI-    617
AIC99919.1_OH131.1     SSG---------SKI-SNGPFNPKALKGGRLKGELILEVPVQNKAIPQKILDEATKNRI-    617
AEP88550.1_TU-B-10     HIGEGGQAAALPK--------SLHPGYGGIHNVE---------KEWGITGVDDQIANRLE    625
ADM39597.1_W23         HIGEGGQAAALPK--------SLHPGYGGIHNVE---------KEWGITGVDDQIANRLE    625
AMR61191.1_ATCC49760   DSQHGKLEKWNRRRTEHLGEFDPVTGKQ--TKK------GEPSRRWDG------------    605

YwqJ_NCIB3610          -----------    602
AKD36832.1_HJ5         -----------    603
BAO93629.1_BEST195     -----------    603
AGE65222.1_XF-1        -----------    621
ADV94423.1_BSn5        -----------    621
AGA21789.1_BSP1        -----------    621
AII38166.1_TO-A        -----------    621
AEP92663.1_RO-NN-1     IIRDINGKVYN    628
AIC99919.1_OH131.1     IIRDINGKVYN    628
AEP88550.1_TU-B-10     TFRKESGR---    633
ADM39597.1_W23         TFRKESGR---    633
AMR61191.1_ATCC49760   -----------    605
```

S4 Fig D

E

```
                            LXG
AID00230.1_OH131.1    MKTLDVHALHEGIQHTIEKLDKQKQQLEKLEKSVEHLAGMKDALKGKGGDAIRTFYEECH    60
AII38200.1_TO-A       MKTLDVHALHEGIQHTIEKLDKQKQQLEKLEKSVEHLAGMKDALKGKGGDAIRTFYEECH    60
YxiD_NCIB3610         MKTLDVHALHEGIQHTIEKLDKQKQQLEKLEKSVEHLAGMKDALKGKGGDAIRTFYEECH    60
ADM39953.1_W23        MKTLDVHALHEGIQHTIEKLDKQKQQLEKLEKSVEHLAGMKDALKGKGGDAIRTFYEECH    60
BAI87628.2_BEST195    MKTLDVHALHEGIQHTIEKLDKQKQQLEKLEKSVEHLAGMKDALKGKGGDAIRTFYEECH    60
AEP88894.1_TU-B-10    MKTLDVHALHEGIQHTIEKLDKQKQQLEKLEKSVEHLAGMKDALKGKGGDAIRTFYEECH    60
AEP92997.1_RO-NN-1    MKTLDVHALHEGIQHTIEKLDKQKQQLEKLEKSVEHLAGMKDALKGKGGDAIRTFYEECH    60
AGE65543.1_XF-1       MKTLDVHALHEGIQHTIEKLDKQKQQLEKLEKSVEHLAGMKDALKGKGGDAIRTFYEECH    60
AGI31091.1_BAB-1      MKTLDVHALHEGIQHTIEKLDKQKQQLEKLEKSVEHLAGMKDALKGKGGDAIRTFYEECH    60
AKD37149.1_HJ5        MKTLDVHALHEGIQHTIEKLDKQKQQLEKLEKSVEHLAGMKDALKGKGGDAIRTFYEECH    60
                      ************************************************************
                            LXG
AID00230.1_OH131.1    KPFLLFFGMFIDEYKKVLKQTQHAISSVESDSHGMIAEAFLSHDARHGVKHAREVTEQLT    120
AII38200.1_TO-A       KPFLLFFGMFIDEYKKVLKQTQHAISSVESDSHGMIAEAFLSHDARHGVKHAREVTEQLT    120
YxiD_NCIB3610         KPFLLFFGIFIDEYKKVLKQTQHAISSVESNSHGMIAEAFLSHDARHGVKHAREVTEQLT    120
ADM39953.1_W23        KPFLLFFGMFIDEYKKVLKQTQHAISSVESDSHGMIAEAFLSHDARHGVKHAREVTEQLT    120
BAI87628.2_BEST195    KPFLLFFGMFIDEYKKVLKQTQHAISSVESDSHGMIAEAFLSHDARHGVKHAREVTEQLT    120
AEP88894.1_TU-B-10    KPFLLFFGIFIDEYKKVLKQTQHAISSVESDSHGMIAEAFLSHDARHGVKHAREVTEQLT    120
AEP92997.1_RO-NN-1    KPFLLFFGMFIDEYKKVLKQTQHAISSVESDSHGMIAEAFLSHDARHGVKHAREVTEQLT    120
AGE65543.1_XF-1       KPFLLFFGMFIDEYKKVLKQTQHAISSVESDSHGMIAEAFLSHDARHGVKHAREVTEQLT    120
AGI31091.1_BAB-1      KPFLLFFGMFIDEYKKVLKQTQHAISSVESDSHGMIAEAFLSHDARHGVKHAREVTEQLT    120
AKD37149.1_HJ5        KPFLLFFGMFIDEYKKVLKQTQHAISSVESDSHGMIAEAFLSHDARHGVKHAREVTEQLT    120
                      ********:*********************:*****************************
                            LXG
AID00230.1_OH131.1    DAVNRQTSAIDHIVSLPTVNDSFFRMETEQAERLISDTLNKLFQFDGQQTQALETAKSDF    180
AII38200.1_TO-A       DAVNRQTSAIDHIVSLPTVNDSFFRMETEQAERLISDTLNKLFQFDGQQTQALEAAKSDF    180
YxiD_NCIB3610         DAVNRQTSAIDHIVSLPTVNDSFFRMETEQAERLISDTLNKLFQFDGQQTQALEAAKSDF    180
ADM39953.1_W23        DAVNRQTSAIGHIVSLPAVNDTFFRMETEQAERLISDTLNKLFQFDGQQTQALETAKSDF    180
BAI87628.2_BEST195    DAVNRQTSAIGHIVSLPAVNDTFFRMETEQAERLISDTLNKLFQFDGQQTQALEAAKSDF    180
AEP88894.1_TU-B-10    DAVNRQTSAIDHIVSLPTVNDTFFRMETEQAERLISDTLNKLFQFDGQQTQALETAKSDF    180
AEP92997.1_RO-NN-1    DAVNRQTSAIGHIVSLPAVNDTFFRMETEQAERLISDTLNKLFQFDGQQTQALEAAKSDF    180
AGE65543.1_XF-1       DAVNRQTSAIDHIVSLPTVNDSFFRMETEQAERLISDTLNKLFQFDGQQTQALETAKSDF    180
AGI31091.1_BAB-1      DAVNRQTSAIDHIVSLPTVNDSFFRMETEQAERLISDTLNKLFQFDGQQTQALETAKSDF    180
AKD37149.1_HJ5        DAVNRQTSAIDHIVSLPTVNDSFFRMETEQAERLISDTLNKLFQFDGQQTQALETAKSDF    180
                      **********.******:***:********************************:*****
                            LXG
AID00230.1_OH131.1    QTMKKYIDQLETMYTGPKIEITGYKSGSILKSQEEENINQTFGAINPQMKQAVDSPMEMM    240
AII38200.1_TO-A       QTMKKYIDQLETMYTGPKIEITGYKSGSILKSQEEENINQIFGAINPQMKQADDSPMEMM    240
YxiD_NCIB3610         QTMKKYIDQLETMYTGPKIEITGYKSGSILKSQEEENINQIFGAINPQMKQADDSPMEMM    240
ADM39953.1_W23        QTMKKYIDQLETMYTGPKIEITGYKSGSILKSQEEENINQTFGTINPQMKEADDSPMEMM    240
BAI87628.2_BEST195    QTMKKYIDQLETMYTGPKIEITGYKSGSILKSQEQENINQTFGAINPQMKQPDDSPMEMM    240
AEP88894.1_TU-B-10    QTMKKYIDQLETMYTGPKIEITGYKSGSILKSQEEEKINQTFGGINSQMKQADDSPMELM    240
AEP92997.1_RO-NN-1    QTMKKYIDQLETMYTGPKIEITGYKSGSILKSQEEENINQTFGAINPQMKQSDDSPMEMM    240
AGE65543.1_XF-1       QTMKKYMDQLETMYTGPKIEITGYKTGSILKSQEEENINQTFGGINSQMKQPDDSPMEMM    240
AGI31091.1_BAB-1      QTMKKYMDQLETMYTGPKIEITGYKTGSILKSQEEENINQTFGGINSQMKQPDDSPMEMM    240
AKD37149.1_HJ5        QTMKKYMDQLETMYTGPKIEITGYKTGSILKSQEEENINQTFGGINSQMKQPDDSPMEMM    240
                      ******:******************:********:*:*** ** ** ***:  *****:*

AID00230.1_OH131.1    LKKLAENEKSKVDSVVKTGDSKKVSKN------------------------IIVINGKVY    276
AII38200.1_TO-A       LKKLAENEKSKVDSVVKTGDSKKVSKN------------------------IIVINGKVY    276
YxiD_NCIB3610         LKKLAENEKSKVDSVVKTGDSKKVSKN------------------------IIVINGKVY    276
ADM39953.1_W23        LKKLAENEKSKVDSVVKTGHSKKVSKN------------------------IIVINGKVY    276
BAI87628.2_BEST195    LKKIEKHKQSNVDIVMKDGKQQKIEREIHADDSNVTVLQKEAAAHPKVYGDIRVINDKLY    300
AEP88894.1_TU-B-10    LKKLEKHKQSNVDTVMKDGKQQKIEREIHADDSNLTVLQKEAAAHPKVYGDIRVINDKLY    300
AEP92997.1_RO-NN-1    LKKLEKHKQSNVDSVMMDDKQQKIEREIHADDSNVTVLQKEAAAHPKVYGDIRVINDKLY    300
AGE65543.1_XF-1       LKKLEKHKQSNVDSVMMDDKQQKIEREIHADDSNLTVLQKEAAAHPKVYGDIRVINDKIY    300
AGI31091.1_BAB-1      LKKLEKHKQSNVDSVMMDDKQQKIEREIHADDSNLTVLQKEAAAHPKVYGDIRVINDKIY    300
AKD37149.1_HJ5        LKKLEKHKQSNVDSVMMDDKQQKIEREIHADDSNLTVLQKEAAAHPKVYGDIRVINDKIY    300
                      ***: ::::*:** *:  ...:*:.::                        * ***.*:*

AID00230.1_OH131.1    NTSEHREHIKTDFS---NAEVKQVVYNDTLYNVYIS---------GNDMKLEPVVSLSDI    324
AII38200.1_TO-A       NTSEHREHIKTDFS---NAEVKQVVYNDTLYNVYIS---------GNDMKLEPVVSLSDI    324
YxiD_NCIB3610         NTSEHREHIKTDFS---NAEVKQVVYNDTLYNVYIS---------GNDMKLEPVVSLSDI    324
ADM39953.1_W23        NTSEHREHIKTDFS---NAEVKQVVYNDTLYNVYIS---------GNDMKLEPVVSLSDI    324
BAI87628.2_BEST195    NNKRLKKIDTIEVIDELTRNTASIDYVGGKYYVYENGQIVREFYAGGKKRLEEVSYIPED    360
AEP88894.1_TU-B-10    NHKGLKKIDTIEVIDELTRDTASIDYVGGKYFVYENGQIVREFYAGGKKRLEEVSYIPED    360
AEP92997.1_RO-NN-1    NNKRLKKIDTIEVIDELTRNTASIDYVGGKYYVYENGQIVREFYAGGKKRLEEVSYIPED    360
AGE65543.1_XF-1       NHKGLKKIDTIEVIDELTRDTASIDYVGGKYYIYENGQIVRELYAGGKKRLEEVSYIPED    360
AGI31091.1_BAB-1      NHKGLKKIDTIEVIDELTRDTASIDYVGGKYYIYENGQIVRELYAGGKKRLEEVSYIPED    360
AKD37149.1_HJ5        NHKGLKKIDTIEVIDELTRDTASIDYVGGKYYIYENGQIVRELYAGGKKRLEEVSYIPED    360
                      * .  ::  . :.    . :. .: * .  * :* .          *.. :** *  : :

AID00230.1_OH131.1    KVDENGYVKILETAVELTGVYDLFKAATGRDPVSGEKVTGKDRVVASINSVPLAKIAKLE    384
AII38200.1_TO-A       KVDENGYVKILETAVELTGVYDLFKAATGRDPVSGEKVTGKDRVVASINSVPFAKIAKLE    384
YxiD_NCIB3610         KVDENGYVKILETAVELTGVYDLFKAATGRDPVSGEKVTGKDRVVASINSVPFAKIAKLE    384
ADM39953.1_W23        KVDENGNVKILETAVELTGVYDLFKAATGRDPVSGEKVTGKDRVVASINSVPFAKIAKLE    384
BAI87628.2_BEST195    K---VGGAKSLDSFLA-GTQYEFIEWVSPQGAVKKLAVRGGKRVVTDVAKHVVEKD----    412
AEP88894.1_TU-B-10    K---VGGAKSLDSFLA-GTQYEFIEWVSPQGAVKKLAVRGGKRVVTDVAKHVAEKD----    412
AEP92997.1_RO-NN-1    K---VGGAKSLDSFLA-GTQYEFIEWVSPQGAVKKLAVRGGKRVVTDVAKQVTEKE----    412
AGE65543.1_XF-1       K---VGGAKPIDSFLA-GTQYEFIEWVSPQGAVKKLAVRGGKRVVTDVAKHVAGKE----    412
AGI31091.1_BAB-1      K---VGGAKPIDSFLA-GTQYEFIEWVSPQGAVKKLAVRGGKRVVTDVAKHVAGKE----    412
AKD37149.1_HJ5        K---VGGAKPIDSFLA-GTQYEFIEWVSPQGAVKKLAVRGGKRVVTDVAKHVAGKE----    412
                      *    * .* ::: :    *:::: .: :. *.   * * .***:.: .    *
```

S4 Fig E continued

E

```
                                                                    toxin
AID00230.1_OH131.1       KLIDINKLINDGKKAKKASEVKNVAKDKGKIANDVSGSANKINSDLIKKYARDIEQRTGR   444
AII38200.1_TO-A          KLIDINKLINNGKKAKKASEVKNVAKDKGKIANDVSGSANKINSDLIKKYARDIEQRTGR   444
YxiD_NCIB3610            KLIDINKLINNGKKAKKASEVKNVAKDKGKIANDVSGSANKINSDLIKKYARDIEQRTGR   444
ADM39953.1_W23           KLIDINKLINDGKKAKKASEVKNVAKDTGNGVPSYGKKS------LPKGPYREVHGFPVK   438
BAI87628.2_BEST195       IKGDVKKKPSKGS-----VNS-------KWWHPGYVDNLSSSQTVIGIKNSPKGLSTLGS   460
AEP88894.1_TU-B-10       IKGDVKKKTPKGS-----VNS-------KWWHPGYVDNLSSSQTVIGIKNSPKGLSTLGS   460
AEP92997.1_RO-NN-1       IKGEVNKVASKGT-----SEI-------GWNMSKGGGQIN------GRKYSQHALERMAP   454
AGE65543.1_XF-1          SKGEVNKVASKGA-----SEG-------KKLV--------SSKTVISSDMEAKILEGQ-R   451
AGI31091.1_BAB-1         SKGEVNKVASKGA-----SEG-------KKLV--------SSKTVISSDMEAKILEGQ-R   451
AKD37149.1_HJ5           SKGEVNKVASKGA-----SEG-------KKLV--------SSKTVISSDMEAKILEGQ-R   451
                            :::*   .*       :                                .
                                                toxin
AID00230.1_OH131.1       ELPKNQIDKLKEA-------LRNK--EYK--KMSPIETAKHR----TKFD----K---VK   482
AII38200.1_TO-A          ELPKNQIDKLKEA-------LRNK--EYK--KMSPIETAKHR----TKFD----K---VK   482
YxiD_NCIB3610            ELPKNQIDKLKEA-------LRNK--EYK--KMSPIETAKHR----TKFD----K---VK   482
ADM39953.1_W23           VKPGAQEKHIP----------NTP--NYK--QE--IANGKNK----SIFY----GDNKTA   474
BAI87628.2_BEST195       STRQNALDAGKGWVGQGAEKMYDKAGNFL---------GYKSVDKMRAFRLQYKPKEKMW   511
AEP88894.1_TU-B-10       STRQNALDAGKGWVGEGAEKMYDKAGNFL---------GYKSVDKMRAFRLQYKPKEKMW   511
AEP92997.1_RO-NN-1       DIPEV-----KAT--------------L---------TNR--AIKKAEELGYKPQTKEF   483
AGE65543.1_XF-1          KVPKNILIGGHSS------SINNANDNFAVEVLSTNADGTKSVMFTKQFVDGNISKIKKS   505
AGI31091.1_BAB-1         KVPKNILIGGHSS------SINNANDNFAVEVLSTNADGTKSVMFTKQFVDGNISKIKKS   505
AKD37149.1_HJ5           KVPKNILIGGHSS------SINNANDNFAVEVLSTNADGTKSVMFTKQFVDGNISKIKKS   505

                                                toxin
AID00230.1_OH131.1       NKVIKEWEENTGQKWPVYKENVVSEKTGKIIRKKGDKYDAHHIIENTFGGEHEWWNMHPA   542
AII38200.1_TO-A          NKVIKEWEENTGQKWPVYKENVVSEKTGKIIRKKGDKYDAHHIIENTFGGEHEWWNMHPA   542
YxiD_NCIB3610            NKVIKEWEENTGQKWPVYKENVVSEKTGKIIRKKGDKYDAHHIIENTFGGEHEWWNMHPA   542
ADM39953.1_W23           QELLDKYAG-----------------KGDFLKNGRERVDFGKPIG-------KYYDRNTG   510
BAI87628.2_BEST195       RANFT-------------ENEI-------------TVVGSKT----------------EL   529
AEP88894.1_TU-B-10       RANFT-------------ENEI-------------TVVGSKT----------------EL   529
AEP92997.1_RO-NN-1       SDFIKKYVDPRNISPSVIEDAI--------M-NTKKIPGNRS---------GTF--VHET   523
AGE65543.1_XF-1          TLFPESWN----------DDQI--------L-KSIIEVGNTPPISTRLRDRSTWH-RASK   545
AGI31091.1_BAB-1         TLFPESWN----------DDQI--------L-KSIIEVGNTPPISTRLRDRSTWH-RASK   545
AKD37149.1_HJ5           TLFPESWN----------DDQI--------L-KSIIEVGNTPPISTRLRDRSTWH-RASK   545
                                                               .
                             toxin
AID00230.1_OH131.1       KFPNEHQAGIHGTGSPANELFKGGKKK-------   569
AII38200.1_TO-A          KFPNEHQAGIHGTGSPANELFKGGKKK-------   569
YxiD_NCIB3610            KFPNEHQAGIHGTGSPANELFKGGKKK-------   569
ADM39953.1_W23           EYVETT-KGLIHYGKDGAHIVPSRP---------   534
BAI87628.2_BEST195       RN---VHVDILD----------------------   538
AEP88894.1_TU-B-10       RN---VHVDILD----------------------   538
AEP92997.1_RO-NN-1       QD---VKVIINEAGDVIT-VIPK-----------   542
AGE65543.1_XF-1          NG---VEIDVIKNGDEVTSGYPTGKVNAPRPSGF   576
AGI31091.1_BAB-1         NG---VEIDVIKNGDEVTSGYPTGKVNAPRPSGF   576
AKD37149.1_HJ5           NG---VEIDVIKNGDEVTSGYPTGKVNAPRPSGF   576
                         .        :
```

S4 Fig E
