# Supplementary material for: Diverse LXG toxin and antitoxin systems specifically mediate intraspecies competition in Bacillus subtilis biofilms
Source: PLoS Genet. 2021 Jul 19;17(7):e1009682. doi: 10.1371/journal.pgen.1009682 (PMC8321402; doi:10.1371/journal.pgen.1009682)
Supplement: S6 Fig — (A) Phylogenetic tree of 79 YeeF homologs from B. subtilis. Seventy-nine YeeF homologs were identified by BLASTp (taxid, 1423) using YeeF as a bait. Possible subgroups are indicated by bars at the right of the clades. Clades containing YeeF-1, 2, 3, or 4 subgroup proteins are labeled. (B) Alignment of C-terminal regions of 79 YeeF toxin homologs. Subgroups are color-coded for ease of viewing. (PDF) [file pgen.1009682.s006.pdf]

A

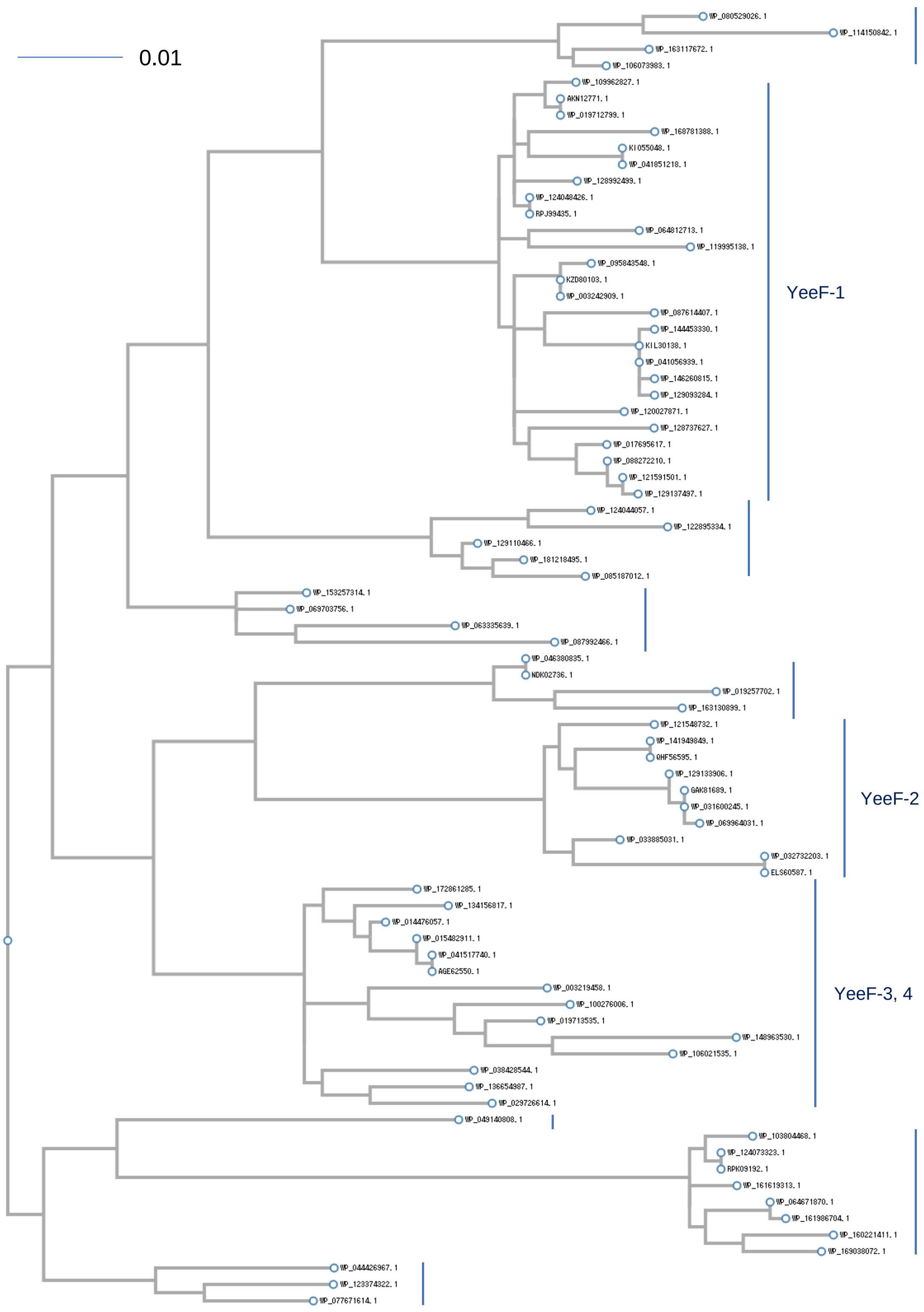
0.01
WP_080529026.1
WP_114150842.1
WP_163117672.1
WP_106073983.1
WP_109962827.1
AKN12771.1
WP_019712799.1
WP_168781388.1
KI055048.1
WP_041851218.1
WP_128992499.1
WP_124048426.1
RPJ99435.1
WP_064812713.1
WP_119995138.1
WP_095843548.1
KZD80103.1
WP_003242909.1
WP_087614407.1
WP_144453330.1
KIL30138.1
WP_041056939.1
WP_146260815.1
WP_129093284.1
WP_120027871.1
WP_128737627.1
WP_017695617.1
WP_088272210.1
WP_121591501.1
WP_129137497.1
YeeF-1
WP_124044057.1
WP_122895334.1
WP_129110466.1
WP_181218495.1
WP_085187012.1
WP_153257314.1
WP_069703756.1
WP_063335639.1
WP_087992466.1
WP_046380835.1
NDK02736.1
WP_019257702.1
WP_163130899.1
WP_121548732.1
WP_141949849.1
QHF56595.1
WP_129133906.1
GAK81689.1
WP_031600245.1
WP_069964031.1
WP_033885031.1
WP_032732203.1
ELS60587.1
YeeF-2
WP_172861285.1
WP_134156817.1
WP_014476057.1
WP_015482911.1
WP_041517740.1
AGE62550.1
WP_003219458.1
WP_100276006.1
WP_019713535.1
WP_148963530.1
WP_106021535.1
WP_038428544.1
WP_136654987.1
WP_029726614.1
YeeF-3, 4
WP_049140808.1
WP_103804468.1
WP_124073323.1
RPK09192.1
WP_161619313.1
WP_064671870.1
WP_161986704.1
WP_160221411.1
WP_169038072.1
WP_044426967.1
WP_123374322.1
WP_077671614.1

B

```
WP_033885031.1      WYNMEQVWSKALSQKPPKQVTVKINNIYEGDSLRPSKFIVVYKVGDKEPVRTIIKNKAGG
WP_141949849.1      WYNMEQVWSKALSQKPPKQVTVKINNIYEGDSLRPSKFIVVYKVGDKEPVRTIIKNKAGG
QHF56595.1          WYNMEQVWSKALSQKPPKQVTVKINNIYEGDSLRPSKFIVVYKVGDKEPVRTIIKNKAGG
WP_129133906.1      WYNMEQVWSKALSQKPPKQVTVKINNIYEGDSLRPSKFIVVYKVGDKEPVRTIIKNKAGG
WP_121548732.1      WYNMEQVWSKALSQKPPKQVTVKINNIYEGDSLRPSKFIVVYKVGDKEPVRTIIKNKAGG
WP_031600245.1      WYNMEQVWSKALSQKPPKQVTVKINNIYEGDSLRPSKFIVVYKVGDKEPVRTIIKNKAGG   YeeF-2
GAK81689.1          WYNMEQVWSKALSQKPPKQVTVKINNIYEGDSLRPSKFIVVYKVGDKEPVRTIIKNKAGG
WP_069964031.1      WYNMEQVWSKALSQKPPKQVTVKINNIYEGDSLRPSKFIVVYKVGDKEPVRTIIKNKAGG
ELS60587.1          WYNMEQVWSKALSQKPPKQVTVKINNIYEGDSLRPSKFIVVYKVGDKEPVRTIIKNKAGG
WP_032732203.1      WYNMEQVWSKALSQKPPKQVTVKINNIYEGDSLRPSKFIVVYKVGDKEPVRTIIKNKAGG
WP_161619313.1      WYKMETEWAAALKEEPPRKVSVRIRPKYLGDSLRPDSFEVIYRIEGKGLFKKFIKNQAGG
WP_124073323.1      WYKMETEWAAALKEEPPRKVSVRIRPKYLGDSLRPDSFEVIYRIEGKGLFKKFIKNQAGG
RPK09192.1          WYKMETEWAAALKEEPPRKVSVRIRPKYLGDSLRPDSFEVIYRIEGKGLFKKFIKNQAGG
WP_103804468.1      WYKMETEWAAALKEEPPRKVSVRIRPKYLGDSLRPDSFEVIYRIEGKGLFKKFIKNQAGG
WP_064671870.1      WYKMETEWAAALKEEPPRKVSVRIRPKYLGDSLRPDSFEVIYRIEGKGLFKKFIKNQAGG
WP_161986704.1      WYKMETEWAAALKEEPPRKVSVRIRPKYLGDSLRPDSFEVIYRIEGKGLFKKFIKNQAGG
WP_160221411.1      WYKMETEWAAALKEEPPRKVSVRIRPKYLGDSLRPDSFEVIYRIEGKGLFKKFIKNQAGG
WP_169038072.1      WYKMETEWAAALKEEPPRKVSVRIRPKYLGDSLRPDSFEVIYRIEGKGLFKKFIKNQAGG
WP_049140808.1      WYKMEQDWAAALQTDPPQKVSVEINALYKGDSLRPTAFRVKYKIGDQEAKLMRIKNQAGG
WP_124044057.1      WYKLEQAWSQALKEEPPKKVTITIENVYESDSLRPTLFDITYKIGEERTKSITIRNQIGG
WP_129110466.1      WYKLEQAWSQALKEEPPKKVTVTIENVYEGDSLRPTLFDITYKIGEERTKSITIRNQIGG
WP_181218495.1      WYKLEQAWSQALKEEPPKKVTVTIENVYEGDSLRPTLFDITYKIGEERTKSITIRNQIGG
WP_085187012.1      WYKLEQAWSQALKEEPPKKVTVTIENVYEGDSLRPTLFDITYKIGEERTKSITIRNQIGG
WP_122895334.1      WYKLEQAWSQALKEEPPKKVTVTIENVYESDSLRPTLFDITYKIGEERTKSITIRNQIGG
WP_106073983.1      WYKMEQVWSKALNGKPPKQVKVNIEPVYQGDSLRPVLFNVEYKIGNRRARQVTIENKPGG
WP_163117672.1      WYKMEQVWSKALNGKPPKQVKVNIEPVYQGDSLRPVLFNVEYKIGNRRARQVTIENKPGG
WP_080529026.1      WYKMEQVWSKALNGKPPKQVKVNIEPVYQGDSLRPVLFNVEYKIGNRRARQVTIENKPGG
WP_114150842.1      WYKMEQVWSKALNGKPPKQVKVNIEPVYQGDSLRPVLFNVEYKIGNRRARQVTIENKPGG
WP_046380835.1      WYEMEQEWAKALKEEPPKRVNVNIESIYKGDSLRPTKFIIEYTIGNKT-KFVTIKNQAGG
NDK02736.1          WYEMEQEWAKALKEEPPKRVNVNIESIYKGDSLRPTKFIIEYTIGNKT-KFVTIKNQAGG
WP_163130899.1      WYEMEQEWAKALKEEPPKRVNVNIESIYKGDSLRPTKFIIEYTIGNKT-KFVTIKNQAGG
WP_019257702.1      WYEMEQEWAKALKEEPPKRVNVNIESIYKGDSLRPTKFIIEYTIGNKT-KFVTIKNQAGG
WP_014476057.1      WYEMEQEWAKALKEEPPQKVNVNIKAIYKGDSLRPDKFIVKYRIGDADFEKVTLKNQSGG
WP_172861285.1      WYEMEQEWAKALKEEPPQKVNVNIKAIYKGDSLRPDKFIVKYRIGDADFEKVTLKNQSGG
WP_015482911.1      WYEMEQEWAKALKEEPPQKVNVNIKAIYKGDSLRPDKFIVKYRIGDADFEKVTLKNQSGG
AGE62550.1          WYEMEQEWAKALKEEPPQKVNVNIKAIYKGDSLRPDKFIVKYRIGDADFEKVTLKNQSGG
WP_041517740.1      WYEMEQEWAKALKEEPPQKVNVNIKAIYKGDSLRPDKFIVKYRIGDADFEKVTLKNQSGG
WP_134156817.1      WYEMEQEWAKALKEEPPQKVNVNIKAIYKGDSLRPDKFIVKYRIGDADFEKVTLKNQSGG
WP_038428544.1      WYEMEQEWAKALKEEPPQKVNVNIKAIYKGDSLRPDKFIVKYRIGDADFEKVTLKNQSGG
WP_136654987.1      WYEMEQEWAKALKEEPPQKVNVNIKAIYKGDSLRPDKFIVKYRIGDADFEKVTLKNQSGG   YeeF-3, 4
WP_029726614.1      WYEMEQEWAKALKEEPPQKVNVNIKAIYKGDSLRPDKFIVKYRIGDADFEKVTLKNQSGG
WP_148963530.1      WYEMEQEWAKALKEEPPQKVNVNIKAIYKGDSLRPDKFIVKYRIGDADFEKVTLKNQSGG
WP_019713535.1      WYEMEQEWAKALKEEPPQKVNVNIKAIYKGDSLRPDKFIVKYRIGDADFEKVTLKNQSGG
WP_100276006.1      WYEMEQEWAKALKEEPPQKVNVNIKAIYKGDSLRPDKFIVKYRIGDADFEKVTLKNQSGG
WP_106021535.1      WYEMEQEWAKALKEEPPQKVNVNIKAIYKGDSLRPDKFIVKYRIGDADFEKVTLKNQSGG
WP_003219458.1      WYEMEQEWAKALKEEPPQKVNVNIKAIYKGDSLRPDKFIVKFRIGDADFEKVTIKNQSGG
WP_119995138.1      WYEMEQEWAKALSKKPPKKVAIQIEPVYSGDSLRPSYFDVTYKIGSRKEISVSIKNQPGG
WP_003242909.1      WYEMEQEWAKALSKKPPKKVAVQIEPVYSGDSLRPSYFDVTYKIGSRKEISVSIKNQPGG
KZD80103.1          WYEMEQEWAKALSKKPPKKVAVQIEPVYSGDSLRPSYFDVTYKIGSRKEISVSIKNQPGG
WP_095843548.1      WYEMEQEWAKALSKKPPKKVAVQIEPVYSGDSLRPSYFDVTYKIGSRKEISVSIKNQPGG
WP_124048426.1      WYEMEQEWAKALSKKPPKKVAVQIEPVYSGDSLRPSYFDVTYKIGSRKEISVSIKNQPGG
RPJ99435.1          WYEMEQEWAKALSKKPPKKVAVQIEPVYSGDSLRPSYFDVTYKIGSRKEISVSIKNQPGG
WP_019712799.1      WYEMEQEWAKALSKKPPKKVAVQIEPVYSGDSLRPSYFDVTYKIGSRKEISVSIKNQPGG
AKN12771.1          WYEMEQEWAKALSKKPPKKVAVQIEPVYSGDSLRPSYFDVTYKIGSRKEISVSIKNQPGG
WP_128992499.1      WYEMEQEWAKALSKKPPKKVAVQIEPVYSGDSLRPSYFDVTYKIGSRKEISVSIKNQPGG
WP_017695617.1      WYEMEQEWAKALSKKPPKKVAVQIEPVYSGDSLRPSYFDVTYKIGSRKEISVSIKNQPGG
WP_088272210.1      WYEMEQEWAKALSKKPPKKVAVQIEPVYSGDSLRPSYFDVTYKIGSRKEISVSIKNQPGG
WP_109962827.1      WYEMEQEWAKALSKKPPKKVAVQIEPVYSGDSLRPSYFDVTYKIGSRKEISVSIKNQPGG
WP_121591501.1      WYEMEQEWAKALSKKPPKKVAVQIEPVYSGDSLRPSYFDVTYKIGSRKEISVSIKNQPGG
KIL30138.1          WYEMEQEWAKALSKKPPKKVAVQIEPVYSGDSLRPSYFDVTYKIGSRKEISVSIKNQPGG   YeeF-1
WP_129137497.1      WYEMEQEWAKALSKKPPKKVAVQIEPVYSGDSLRPSYFDVTYKIGSRKEISVSIKNQPGG
WP_120027871.1      WYEMEQEWAKALSKKPPKKVAVQIEPVYSGDSLRPSYFDVTYKIGSRKEISVSIKNQPGG
WP_144453330.1      WYEMEQEWAKALSKKPPKKVAVQIEPVYSGDSLRPSYFDVTYKIGSRKEISVSIKNQPGG
WP_087614407.1      WYEMEQEWAKALSKKPPKKVAVQIEPVYSGDSLRPSYFDVTYKIGSRKEISVSIKNQPGG
WP_128737627.1      WYEMEQEWAKALSKKPPKKVAVQIEPVYSGDSLRPSYFDVTYKIGSRKEISVSIKNQPGG
WP_146260815.1      WYEMEQEWAKALSKKPPKKVAVQIEPVYSGDSLRPSYFDVTYKIGSRKEISVSIKNQPGG
KIO55048.1          WYEMEQEWAKALSKKPPKKVAVQIEPVYSGDSLRPSYFDVTYKIGSRKEISVSIKNQPGG
WP_041851218.1      WYEMEQEWAKALSKKPPKKVAVQIEPVYSGDSLRPSYFDVTYKIGSRKEISVSIKNQPGG
WP_064812713.1      WYEMEQEWAKALSKKPPKKVAVQIEPVYSGDSLRPSYFDVTYKIGSRKEISVSIKNQPGG
WP_168781388.1      WYEMEQEWAKALSKKPPKKVAVQIEPVYSGDSLRPSYFDVTYKIGSRKEISVSIKNQPGG
WP_129093284.1      WYEMEQEWAKALSKKPPKKVAVQIEPVYSGDSLRPSYFDVTYKIGSRKEISVSIKNQTGG
WP_153257314.1      WYEMEQEWAKALKEEPPETVSVQIENVYQGDSLRPTLFKVKYKVGDGRYKNMTIRNQAGG
WP_069703756.1      WYEMEQEWAKALKEEPPETVSVQIENVYQGDSLRPTLFKVKYKVGDGRYKNMTIRNQAGG
WP_063335639.1      WYEMEQEWAKALKEEPPETVSVQIENVYQGDSLRPTLFKVKYKVGDGRYKNMTIRNQAGG
WP_087992466.1      WYEMEQEWAKALKEEPPETVSVQIENVYQGDSLRPTLFKVKYKVGDGRYKNMTIRNQAGG
WP_123374322.1      WYEMEQEWAKALKEEPPKLVTIEIKPIYSGDSLRPNLFKVKYKIGNKRFKNVRIKNQAGG
WP_077671614.1      WYEMEQEWAKALKEEPPKLVTIEIKPIYSGDSLRPNLFKVKYKIGNKRFKNVRIKNQAGG
WP_044426967.1      WYEMEQEWAKALKEEPPKLVTIEIKPIYSGDSLRPNLFKVKYKIGNKRFKNVRIKNQAGG
                    **::*  *: **. .**. * : *.  * .*****  * : : :         :.*: **
```
