# Supplementary material for: Diverse LXG toxin and antitoxin systems specifically mediate intraspecies competition in Bacillus subtilis biofilms
Source: PLoS Genet. 2021 Jul 19;17(7):e1009682. doi: 10.1371/journal.pgen.1009682 (PMC8321402; doi:10.1371/journal.pgen.1009682)
Supplement: S2 Table — (DOCX) [file pgen.1009682.s013.docx]

**S2 Table. Primers used in this study.**

| Primers | Sequences (5’ to 3’) |
| --- | --- |
| **Gene disruptions** | |
| pUC-F | GTTTTCCCAGTCACGACG |
| pUC-R | GAATTGTGAGCGGATAAC |
| yeeFG-F1 | GGAGGATCCCAAGCGGAGGCAAGGTAAGG |
| yeeFG-R1 | GTTATCCGCTCACAATTCGTACTCTTTTGCACGGTCTG |
| yeeFG-F2 | CGTCGTGACTGGGAAAACTTTGCCTGAAGATGAGGATG |
| yeeFG-R2 | AGCATCGGATTCATGTAGTCG |
| yobLK-F1 | GGGTGATGTATGTGACTAAC |
| yobLK-R1 | GTTATCCGCTCACAATTCAATCAGCCTCAAATACCTTC |
| yobLK-F2 | CGTCGTGACTGGGAAAACACTGGGATGAGGATGAAGAC |
| yobLK-R2 | GCAAATGCTGAAGCACCGAC |
| yokIJ-F1 | TTCATAAGACGGTAAATGGC |
| yokIJ-R1 | GTTATCCGCTCACAATTCGAGAGCAATGAATCAGCTTC |
| yokIJ-F2 | CGTCGTGACTGGGAAAACCGATGCATATGATGGCATAG |
| yokIJ-R2 | GCTCGCAGTGTAAGATTCTC |
| yqcGF-F1 | AACTCTCGCACCTACTCATG |
| yqcGF-R1 | GTTATCCGCTCACAATTCGTGAGCAAGGTTTTGGCTTC |
| yqcGF-F2 | CGTCGTGACTGGGAAAACGCTTTACCTATATCCGAAGG |
| yqcGF-R2 | AGAAGAAAGGCTTGACAACC |
| yukED-F1 | TCGCTGATATGATCAGCCAC |
| yukED-R1 | GTTATCCGCTCACAATTCCTGCCATATTCCTCATTACC |
| yukED-F2 | CGTCGTGACTGGGAAAACCTGAGATGCAGGCCGTCATC |
| yukED-R2 | CTTGGTCGATTAATTGGTCC |
| wapA-F1 | ACAATCTCAGTCCTTACACG |
| wapA-R1 | GTTATCCGCTCACAATTCTTCTTCTGGATCATCGGCAG |
| wapI-F2 | CGTCGTGACTGGGAAAACGTCGAACTCCAACTTGTTCG |
| wapI-R2 | ACGGGTGTTGGCTGGTATTG |
| **In-frame deletion** | |
| yukE-D-F3 | GTACCCGGGAGCTCGAATTCTGTGTACTGTAGGCAAGCAG |
| yukE-D-R3 | TCGTATTGATCTGTAGCTCTTCGGGTGTGACAC |
| yukE-D-F4 | ACCCGAAGAGCTACAGATCAATACGAGCAGCTC |
| yukE-D-R4 | CGTCGGGCGATATCGGATCCGCCTCTGCGGAGAACGTCAG |
| yukC-D-F1 | GTACCCGGGAGCTCGAATTCGGCGAAGCAATACGGCGTTG |
| yukC-D-R1 | CTAACGATGTTTCCACGTTAGCTTCAAGACCGTC |
| yukC-D-F2 | TTGAAGCTAACGTGGAAACATCGTTAGTCGATAC |
| yukC-D-R2 | CGTCGGGCGATATCGGATCCTGGCGTTGATTTTCTCTCCG |
| ***gfp* reporters** | |
| yeeFG-F1 | GGAGGATCCCAAGCGGAGGCAAGGTAAGG |
| yeeF-P-R1 | AAGAAGCTTGTACTCTTTTGCACGGTCTG |
| yobL-P-F1 | GGAGGATCCCTTTGAGGCTAAACACCAGC |
| yobL-P-R1 | AAGAAGCTTAATCAGCCTCAAATACCTTC |
| yokI-P-F1 | GGAGGATCCGCTTTGAGGCTAAACACCAG |
| yokI-P-R1 | AAGAAGCTTGAGAGCAATGAATCAGCTTC |
| yqcG-P-F1 | GGAGGATCCAATGCTTAGAAGCTCTAGGG |
| ywqH-P-F2 | GGAGGATCCCAGACATGGCCGACCAGATG |
| ywqH-P-R2 | AAGAAGCTTCGATCTTGTCTTCCACGTCTG |
| yxiB-P-F1 | GGAGGATCCAATACCAGCGGTGAGGCTGT |
| yxiB-P-R1 | AAGAAGCTTATTGATGGATCTGTTCCTGC |
| wapA-P-F1 | GGAGGATCCTACATCGGCTGGCACTAATG |
| wapA-P-R1 | AAGAAGCTTGATCATATGGGCAGTTCTCG |
| spac-hy-P-F1 | GGAGGATCCCCCTTGCCTACCTAGCTTCC |
| pMUT-R | AGTGTATCAACAAGCTGG |
| **P*_spac_*_-hy_-antitoxin genes** | |
| yezG-F1 | AAGAAGCTTAACCTACAAAATTGGGAGC |
| yezG-R1 | GGAGGATCCTAATTATAGACCGAGGTAAC |
| yobK-F1 | AAGAAGCTTATTGGAAATGGAGAGCGAAAC |
| yobK-R1 | GGAGGATCCTATTGCGCTATAGCAATAC |
| yokJ-F1 | AAGAAGCTTCCTAAAGAAGCTAGACAGC |
| yokJ-R1 | GGAGGATCCGTTCGATCTTAGTGAAGCTC |
| yqcF-F1 | AAGAAGCTTATGACGGATGATTACTTTGGAG |
| yqcF-R1 | GGAGGATCCTCTTCTATTATTTGCAGCTG |
| ywqK-F1 | AAGAAGCTTCCTAGATGTCCACATTGTG |
| ywqK-R1 | GGAGGATCCACGATTGCTCACTCTCGACC |
| yxxD-F1 | AAGAAGCTTCATGGTACAGGCTCACCAGC |
| yxxD-R1 | AAAGAAGATCTGTCATCTTGCTCCTTTAAGG |
| **P*_spac_*_-hy_-toxin genes** | |
| yeeF-F4 | AGCGGATAACAATTAAGCTTCACCAAAACACGTTCTCACC |
| yeeF-R4 | ATCAACAAGCTGGGGATCCCCTTGCATCTTCCTAGTTTCC |
| yobL-F4 | AGCGGATAACAATTAAGCTTTTACATAGAAGTAGTCTAGG |
| yobL-R4 | ATCAACAAGCTGGGGATCCCTTCATGACTTGCACCTTCTG |
| yokI-F4 | AGCGGATAACAATTAAGCTTTAGAGTAGGCTTGAAAGGAG |
| yokI-R4 | ATCAACAAGCTGGGGATCCCACATGTCAATGCTCAAGTCC |
| yqcG-F4 | AGCGGATAACAATTAAGCTTGTTTCATCATTCTTCCATGG |
| yqcG-R4 | ATCAACAAGCTGGGGATCCCTAGACAATCTCTCCTTAATC |
| ywqJ-F6 | AGCGGATAACAATTAAGCTTGAGCAGGACGAGGCGATAGC |
| ywqJ-R4 | ATCAACAAGCTGGGGATCCCAAATCCTCAAAGTCCACTCC |
| yxiD-F4 | AGCGGATAACAATTAAGCTTACGAAAACATATCATCTTCC |
| yxiD-R4 | ATCAACAAGCTGGGGATCCCATCTCACTTTGATTCACTGG |
| **P*_spac_*_-hy_-toxin-antitoxin genes** | |
| yeeF-F4 | AGCGGATAACAATTAAGCTTCACCAAAACACGTTCTCACC |
| yezG-R4 | ATCAACAAGCTGGGGATCCCTAATTATAGACCGAGGTAAC |
| yobL-F4 | AGCGGATAACAATTAAGCTTTTACATAGAAGTAGTCTAGG |
| yobK-R4 | ATCAACAAGCTGGGGATCCCCTATTGCGCTATAGCAATAC |
| yokI-F4 | AGCGGATAACAATTAAGCTTTAGAGTAGGCTTGAAAGGAG |
| yokJ-R4 | ATCAACAAGCTGGGGATCCCATTGCACATCTTTAGCAATG |
| yqcG-F4 | AGCGGATAACAATTAAGCTTGTTTCATCATTCTTCCATGG |
| yqcF-R4 | ATCAACAAGCTGGGGATCCCTCATTAAACAACAGATTCTC |
| ywqJ-F6 | AGCGGATAACAATTAAGCTTGAGCAGGACGAGGCGATAGC |
| ywqK-R4 | ATCAACAAGCTGGGGATCCCACGATTGCTCACTCTCGACC |
| yxiD-F4 | AGCGGATAACAATTAAGCTTACGAAAACATATCATCTTCC |
| yxxD-R4 | ATCAACAAGCTGGGGATCCCGTCATCTTGCTCCTTTAAGG |
| **P*_spac_*_-hy_-*yukE* and P*_spac_*_-hy_-*yukC* genes** | |
| yukE-F7 | AGCGGATAACAATTAAGCTTTAGGCTGATGGCACAGGAGG |
| yukE-R7 | ATCAACAAGCTGGGGATCCCTGTTCACCTGACATTCAACC |
| yukC-F1 | AGCGGATAACAATTAAGCTTTACAAACGGAGACCGGCTTG |
| yukC-R1 | ATCAACAAGCTGGGGATCCCACCTGCGCTTCATGTCTCAC |
