# Supplementary material for: Diverse LXG toxin and antitoxin systems specifically mediate intraspecies competition in Bacillus subtilis biofilms
Source: PLoS Genet. 2021 Jul 19;17(7):e1009682. doi: 10.1371/journal.pgen.1009682 (PMC8321402; doi:10.1371/journal.pgen.1009682)
Supplement: S1 Text — (DOCX) [file pgen.1009682.s014.docx]

**S1 TEXT**

**Construction of *B. subtilis* strains.** Since strain NCIB 3610 has low competence ability, mutant alleles were first introduced into the domesticated strain 168 and then transferred to strain NCIB 3610 via transformation with genomic DNA [1]. Strain 168 has several mutations that affect biofilm formation. The possibility of introducing these unwanted biofilm defects into strain NCIB 3610 was eliminated by examining the colony morphology of transformants.

***gfp* transcriptional reporter strains.** The *yeeF* promoter region was amplified by PCR with primers *yeeF*-P-F1 and *yeeF*-P-R1 (S2 Table). The PCR products were digested with *Eco*RI and *Hin*dIII, and then ligated with the *Eco*RI- and *Hin*dIII-digested plasmid pDCG3, which is an *amyE* integration vector containing promoterless *gfp mut2* and *cat*. Likewise, promoter regions of *yobL, yokI, yqcG, ywqH, yxiB,* and *wapA* were amplified by PCR and cloned into pDCG3. Primers used are listed in S2 Table. To construct the constitutively expressed *gfp*-reporter strain, the *spac*-hy promoter region was amplified from plasmid pMutinT3-hy [2] using primers spac-hy-P-F1 and pMUT-R. The PCR products were digested with *Eco*RI and *Hin*dIII, and then ligated with the *Eco*RI- and *Hin*dIII-digested plasmid pDCG3. The resultant plasmids were used to transform strain 3610 to obtain *gfp*-reporter strains. Note that the P*_spac_*_-hy_-*gfp*-reporter strain does not contain *lacI* and therefore constitutively expresses *gfp*.

**Deletion mutants.** Deletion of *yeeF-yeeG* was carried out using an overlap-extension PCR technique. A *cat* cassette was amplified from pCBB31 [1] by PCR using pUC-R and pUC-F primers (S2 Table). Upstream and downstream regions of *yeeF-yeeG* were amplified by PCR using the primer pairs *yeeF*-F1/*yeeF*-R1 and *yeeF*-F2/*yeeF*-R2, respectively. The 5′ sequences of *yeeF*-R1 and *yeeF*-F2 were complementary to the sequences of pUC-R and pUC-F, respectively. To fuse the three PCR fragments, all three were mixed and used as template for a second round of PCR with primers *yeeF*-F1 and *yeeF*-R2. The resultant PCR products were used to transform strain 168, generating the 168 Δ*yeeF*–*G*::*cat* strain. The Δ*yeeF*–*G*::*cat* mutation was then transferred to NCIB 3610. Δ*yobL*–*K*::*cat*, Δ*yokI*–*J*::*cat*, Δ*yqcG*–*F*::*cat*, and Δ*wapAI*::*cat* mutants were constructed via the same procedure using different primer sets (S2 Table). If necessary, the *cat* marker was replaced with other antibiotic resistance makers using plasmids described by Steinmetz and Richter [3].

**Markerless in-frame deletion mutants of *yukE* and *yukC*.** To delete *yukE*, upstream and downstream regions of *yukE* were amplified using primer sets, *yukE*-D-F3/*yukE*-D-R3 and *yukE*-D-F4/*yukE*-D-R4, respectively (S2 Table). 5’ parts of these primers contain sequences for the restriction enzyme-free cloning strategy. Two PCR products were cloned into the *Eco*RI and *Bam*HI-digested pMAD vector [4] using Gibson assembly master mix (New England Biolabs, Massachusetts, USA) to obtain plasmid pMAD*yukE*. This plasmid was introduced into NCIB3610 by transformation. Insertion and excision of pMAD*yukE* into and out of the chromosome of NCIB3610 was performed as previously described [4]. Erythromycin sensitive white colonies were tested by PCR using primers *yukE*-D-F3 and *yukE*-D-R4, and the *yukE* deletion mutant was isolated. The deletion mutant of *yukC* was constructed via the same procedure using different primer sets (S2 Table).

**Antitoxin-expression strains.** The entire *yezG* region including the Shine–Dalgarno sequence was amplified by PCR using primers yezG-F1 and yezG-R1. The PCR products were digested with *Hin*dIII and *Bam*HI, and then ligated with the *Hin*dIII- and *Bam*HI-digested plasmid pDLT3-hy [2], which is an *amyE* integrative vector that contains *cat*, the *spac*-hy promoter, and *lacI* between the *amyE* upstream and downstream sequences. The entire regions of *yobK*, *yokJ*, *yqcF*, *ywqK*, and *yxxE* were also amplified by PCR and cloned into pDLT3-hy. The resultant plasmids were used to transform strain 3610 and obtain antitoxin-expression strains. The *cat* marker in these mutants was replaced with the *erm* marker using the pCM::EM plasmid [3].

**Toxin-, toxin-antitoxin-, *yukE*-, and *yukC*-expression strains.** These genes were amplified by PCR using primers listed in S2 Table. 5’ 20 nt sequences of these primers are complementary to the sequences of pDLT3-hy, upstream of the *Hin*dIII site or downstream of the *Bam*HI site. PCR products were mixed with *Hin*dIII and *Bam*HI-digested pDLT3-hy, and two DNA fragments were ligated at 50ºC for 2 h using Gibson assembly master mix (New England Biolabs, Massachusetts, USA). The ligation mixtures were used to transform strain 168. Among Cm^r^ transformants, toxin- and toxin-antitoxin-expression strains were identified by PCR. Expression constructs in the resultant strains were then transferred to NCIB 3610.

**References**

1. Kobayashi K. *Bacillus subtilis* pellicle formation proceeds through genetically defined morphological changes. J Bacteriol. 2007 Jul;189(13):4920-31. pmid: 17468240
2. Kobayashi K, Ikemoto Y. Biofilm-associated toxin and extracellular protease cooperatively suppress competitors in *Bacillus subtilis* biofilms. PLoS Genet. 2019 Oct 17;15(10):e1008232. pmid: 31622331
3. Steinmetz M, Richter R. Plasmids designed to alter the antibiotic resistance expressed by insertion mutations in *Bacillus subtilis*, through *in vivo* recombination. Gene. 1994 May 3;142(1):79-83. pmid: 8181761
4. Arnaud M, Chastanet A, Débarbouillé M. New vector for efficient allelic replacement in naturally nontransformable, low-GC-content, gram-positive bacteria. Appl Environ Microbiol. 2004 Nov;70(11):6887-91. pmid: 15528558
